# Genome-wide identification of *Xenopus *matrix metalloproteinases: conservation and unique duplications in amphibians

**DOI:** 10.1186/1471-2164-10-81

**Published:** 2009-02-17

**Authors:** Liezhen Fu, Biswajit Das, Smita Mathew, Yun-Bo Shi

**Affiliations:** 1 Section on Molecular Morphogenesis, PCRM, NICHD, NIH, Bldg. 18T, Rm. 106, Bethesda, MD 20892, USA

## Abstract

**Background:**

Matrix metalloproteinases (MMPs) are members of the superfamily of Zn^2+ ^dependent extracellular or membrane-bound endopeptidases which have been implicated to play critical roles in vertebrate development and human pathogenesis. A number of MMP genes have been found to be upregulated in some or all organs during frog metamorphosis, suggesting that different MMPs may have different functions in various organs/tissues. The recent advances in EST (expressed sequence tag) sequencing and the completion of the genome of *Xenopus (X.) tropicalis *prompted us to systematically analyze the existence of MMPs in the *Xenopus *genome.

**Results:**

We examined *X. laevis *and *X. tropicalis *ESTs and genomic sequences for MMPs and obtained likely homologs for 20 out of the 25 MMPs known in higher vertebrates. Four of the five missing MMPs, i.e. MMPs 8, 10, 12 and 27, were all encoded on human Chromosome 11 and the other missing MMP, MMP22 (a chicken MMP), was also absent in human genome. In addition, we identified several novel MMPs which appears to be derived from unique duplications over evolution, are present in the genomes of both *Xenopus *species.

**Conclusion:**

We identified the homologs of most of the mammalian MMPs in *Xenopus *and discovered a number of novel MMPs. Our results suggest that MMP genes undergo dynamic changes over evolution. It will be of interest in the future to investigate whether MMP expression and functions during vertebrate development are conserved. The sequence information reported here should facilitate such an endeavor in the near future.

## Background

Matrix metalloproteinases (MMPs) are Zn^2+ ^dependent extracellular or membrane-bound proteinases with overlapping substrate specificities [1-6]. They are capable of cleaving proteinaceous components of the extracellular matrix (ECM) as well as non-ECM proteins [2-5,7,8], thus affecting cell fate through modifications of cell's microenvironment. MMPs have a similar domain structure that includes a prepeptide for secretion, a propeptide to maintain latency, and a catalytic domain, featured by the signature sequence HEFGHXXH, for substrate cleavage. The catalytic domain binds to a Zn^2+ ^ion through the three-histidine residues within the signature sequence to form the catalytic center [5,9,10]. The propeptide contains a highly conserved sequence, PRCGXPD, the so called "cysteine switch", within which the cysteine residue interacts with the catalytic Zn^2+ ^to maintain enzyme latency [11]. Most MMPs are secreted as latent enzymes and processed to the active forms upon the removal of the propeptide domain through various mechanisms. Other MMPs, such as stromelysin 3 (ST3, also known as MMP11), MMP21, MMP23, MMP28, and membrane type MMPs (MT-MMPs) are activated intracellularly through the removal of the propeptide domain by furin, a Golgi enzyme [3,12,13].

MMP expression and distribution have long implicated that MMPs play important roles in many physiological processes including embryonic development, angiogenesis, tissue resorption and remodeling, and pathological events such as tumor invasion and arthritis [8,14-22]. *In vitro *and cell culture studies have provided strong evidence to show that MMPs can regulate cell fate and behavior by remodeling the ECM. On the other hand, increasing evidences indicate that MMPs are capable of cleaving non-ECM extracellular or membrane-bound proteins, suggesting the existence of multiple pathways for MMPs to regulate cells. Despite the extensive *in vitro *and cell culture studies, the *in vivo *functions of MMPs are poorly understood. Surprisingly, with a few exceptions, transgenic overexpression of MMPs and MMP knockouts in mouse have little or weak phenotypes on mouse development [23,24]. This appears to be at least in part due to the redundancy in MMP expression and function. These findings emphasize the need for further *in vivo *studies by using different model systems.

Frog metamorphosis offers a unique opportunity to study MMP function during postembryonic development in vertebrates. This process is totally dependent on the presence of thyroid hormone (TH) and mimics the postembryonic period from a few months before to several months after birth in humans [25-27]. During metamorphosis, dramatic tissue-specific remodeling occurs through TH-regulated cell fate changes. These include complete absorption of the gill and the tail, *de novo *generation of the limbs, and remodeling of most other organs such as the intestine. For example, in the intestine, the larval epithelial cells die through apoptosis and adult epithelial progenitor cells, which may be derived from de-differentiated larval epithelial cells, proliferate and eventually differentiate to form a multiply folded adult epithelium [28-31]. Numerous studies have shown that the metamorphic effects of TH are mediated by thyroid hormone receptors, which control a gene regulation cascade by regulating the transcription of the so-called direct TH-response genes. These direct response genes in turn affect the expression of indirect TH-response genes to eventually regulate cell fate and behavior during metamorphosis. Initial isolation and characterization of TH-response genes revealed that *Xenopus (X.) laevis *ST3 (MMP11) and collagenase 3 (MMP13), and *Rana catesbeiana *collagenase 1 (MMP1) are regulated by TH during metamorphosis. Subsequent studies have found that essentially all MMPs analyzed so far are regulated by TH in at least some organs/tissues during metamorphosis [32-48]. Among them, ST3 and MMP9-TH in *X. laevis *and collagenase 1 in *Rana catesbeiana *have been shown to be direct response genes with thyroid hormone response elements present in their promoters [43,49,50]. Furthermore, *in vitro *organ culture analysis and *in vivo *analyses have provided strong evidence for the participation of MMPs in metamorphosis [40,41,51-54]. For example, we have demonstrated that ST3 is required for TH-induced ECM remodeling, intestinal larval epithelial apoptosis as well as adult epithelial cell migration in organ cultures and that transgenic overexpression of ST3 alone at premetamorphic stages, e.g., stage 54, can induce larval epithelial apoptosis and ECM remodeling in the intestine in the absence of TH [52,53]. These functional studies directly proved the function of ST3 as first suggested based on expression analyses. Since all MMPs analyzed so far are regulated by TH during metamorphosis, it is pertinent to ask whether the rest of the MMPs are also regulated by TH and whether different MMPs have different functions during metamorphosis in different organs/tissues.

As an initiative to begin to address these important issues, we have carried out a genome-wide analysis of MMP genes in both *X. laevis *and *X. tropicalis *through a bioinformatic approach by making use of the genome sequence information for *X. tropicalis *and cDNA sequences available for *X. laevis *and *tropicalis *genes from the NIH Frog Initiatives Program. We demonstrate that essentially all mammalian MMPs have homologs in *Xenopus*, although the homologs for some MMPs cannot be assigned with certainty. Furthermore, we have discovered a number of novel MMPs and duplications that are uniquely present in the amphibian genome.

## Results and discussion

### Bioinformatic search for *Xenopus *MMPs

Many *X. laevis *MMPs were previously cloned [32,33,39,41,43,44,46,47,55-58]. These cDNA sequences were used to search for other MMPs in the public EST database at the NCBI  and the Gene Index Project in Computational Biology and Function Genomics Laboratory . Putative MMP protein sequences that were derived from the retrieved cDNA sequences were pooled and analyzed on a phylogenetic tree. Closely related entries were compared pair-wise by using MacVector (Accelrys Inc., San Diego, CA) and redundant sequences were removed. The resulting *X. laevis *MMPs were listed in Table 1 (see Additional file 1 for their nucleotide sequences). Compared to human MMPs, some *X. laevis *MMPs were missing from the list and some others had highly homologous duplicates, likely due to the pseudotetraploid *X. laevis *genome. The missing ones could be due to either the absence of the genes in *Xenopus *genome or incomplete sequence data available. Thus, we also searched and analyzed the cDNA sequences for MMPs in the highly related species, *X. tropicalis*. We aligned the cDNA sequences of *X. laevis *or *tropicalis *MMPs to the JGI *X. tropicalis *genomic scaffolds  (Table 1). When needed, we also used human MMP sequences to search the *X. tropicalis *genome database to ensure a complete search of the genome.

**Table 1:** The amino acid identities of Xl-MMPs compared to their counterpart Xt-MMPs#.

*Xenopus laevis*		*Xenopus tropicalis*			
Name	Amino Acids	Name	Amino Acids	Scaffold	Identity (%)
Xl-MMP1A	466	Xt-MMP1	466	119	87
Xl-MMP1B	466				87
Xl-MMP2	656	Xt-MMP2	655	458	94
Xl-MMP3	458	Xt-MMP3	497	119	84
Xl-MMP7A	252	Xt-MMP7	259	119	87
Xl-MMP7B	259				81
Xl-MMP9	671	Xt-MMP9	670	29	87
Xl-MMP9TH	683	Xt-MMP9TH	683	29	91
Xl-MMP11	477	Xt-MMP11	477	12	93
Xl-MMP13	469*	Xt-MMP13	472	119	93
Xl-MMP13A	472				93
Xl-MMP14A	575	Xt-MMP14	578	792	94
Xl-MMP14B	576				93
Xl-MMP15	262*	Xt-MMP15	648	6	97
Xl-MMP16	592	Xt-MMP16	607	452	94
Xl-MMP17	159*	Xt-MMP17	588	12	95
Xl-MMP18	467	Xt-MMP18	467	119	86
Xl-MMP19	123*	Xt-MMP19	476	101	95
Xl-MMP20	478	Xt-MMP20	458	119	89
Xl-MMP21	604	Xt-MMP21	604	32	92
Xl-MMP23	381	Xt-MMP23	335*	414	80
Xl-MMP24A	361*	Xt-MMP24	603	954	99
Xl-MMP24B	247*				97
Xl-MMP25	546	Xt-MMP25	545	1214	89
Xl-MMP26	258	Xt-MMP26	261	119	88
Xl-MMP28A	496	Xt-MMP28	499	72	92
Xl-MMP28B	497				91
Xl-MMP N1	562*	Xt-MMP N1	573	501	90
Xl-MMP N3	519*	Xt-MMP N3	627	508	91
		Xt-MMP N2	260	119	N/A
		Xt-MMP N4	455	119	N/A
		Xt-MMP N5	422	119	N/A
		Xt-MMP N6	364	132	N/A

#Comparison of *X. laevis *and *X. tropicalis *MMPs. Pair-wise comparisons were done to obtain the percent of identities between the MMPs from the two species (*: incomplete sequences).

**Figure 1:**
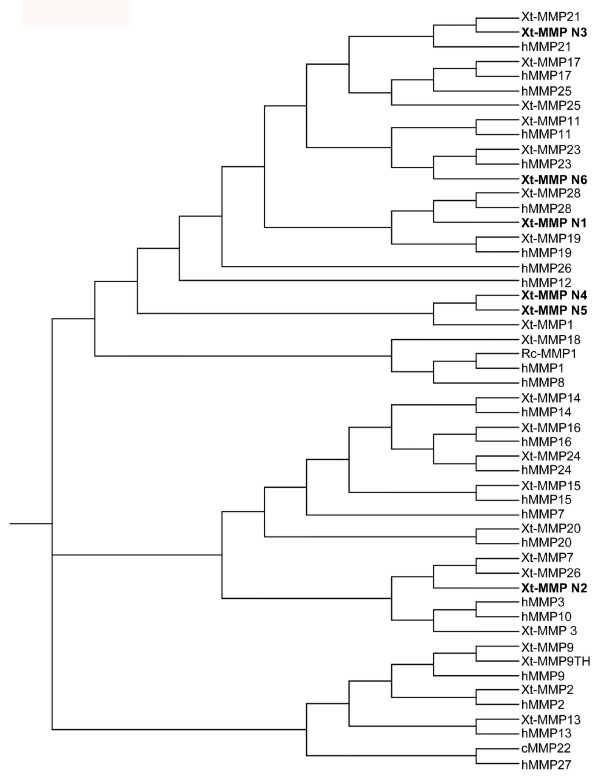
**Phylogenetic tree of *X. tropicalis *(Xt) and human MMPs**. Also included are chicken MMP22 (cMMP22) and *Rana catesbeiana *MMP1 (Rc-MMP1) as MMP22 was not found in human and *Xenopus *and Rc-MMP1 has a unique sequence organization (see description on MMP1). Novel *X. tropicalis *MMPs are highlighted in bold.

Pair-wise sequence comparison (not shown) and phylogenetic analysis (Fig. 1) allowed us to assign most of the *Xenopus *MMPs to the corresponding human homologs (except that chicken MMP22 was also used since MMP22 is absent in human [57]) (where an MMP name had been previously assigned in the databases, we kept the same name for consistency. As we discussed below, some of these MMPs may not be the true homologs of the human MMPs as currently assigned). As shown in table 1, all *X. laevis *MMPs have a corresponding homolog in *X. tropicalis *and the homologs are highly conserved with over 80% amino acid sequence identities between *X. laevis *and *X. tropicalis*. These include a duplicated gelatinase B (MMP9TH) that is absent in mouse and human genome. As *X. laevis *is a pseudotetraploid organism, it is not surprising that some MMPs (MMP1, MMP7, MMP13, MMP14, MMP24, MMP28) have duplicate copies in *X. laevis *but only one in *X. tropicalis*. Only four *X. tropicalis *MMPs (MMP N2, MMP N4, MMP N5, and MMP N6, see below for more on these MMPs) have no available homologs in *X. laevis *yet, likely due to incomplete cDNA sequence information available for *X. laevis*.

### *Xenopus *MMPs with known human homologs

Phylogenetic (Fig. 1) and pair-wise sequence (table 2) analyses suggest that the following human MMPs have true homologs in *Xenopus *genome: MMP2, MMP9, MMP11, MMP13, MMP14, MMP15, MMP16, MMP17, MMP19, MMP20, MMP21, MMP23, MMP24, MMP25, MMP28. The homologous human and *X. tropicalis *MMPs cluster together in the phylogenetic tree (Fig. 1) and share highest sequence identities with each other than with any other MMPs (one exception to this is *X. tropicalis *MMP25, which shares similar homologies with human MMP17 and MMP25. Since the structurally related *X. tropicalis *MMP17 share 70% homology with the human MMP17 but only 47% homology with the human MMP25, we assigned this MMP as *X. tropicalis *MMP25) (table 2). In addition, the homologous MMPs have similar lengths and domain organizations (data not shown).

**Table 2:** Percent homology between *Xenopus tropicalis *and human MMP proteins#.

**MMP**	**H1**	**H2**	**H3**	**H7**	**H8**	**H9**	**H10**	**H11**	**H12**	**H13**	**H14**	**H15**	**H16**	**H17**	**H19**	**H20**	**H21**	**C22**	**H23**	**H24**	**H25**	**H26**	**H27**	**H28**
***X*1**	51	42	54	48	52	36	51	32	51	53	38	38	40	36	33	46	28	50	25	41	36	39	49	33
***X*2**	44	**80**	44	49	43	48	44	38	42	48	32	24	32	30	30	40	27	44	27	32	32	39	38	29
***X*3**	48	38	50	50	47	33	47	33	46	46	36	36	37	31	30	43	27	45	26	37	31	41	42	31
***X*7**	54	54	55	51	53	49	53	35	55	55	44	45	46	38	36	51	36	53	29	47	39	43	54	41
***X*9**	37	50	40	41	38	**57**	40	34	37	41	25	24	26	26	32	34	24	38	32	23	30	37	34	27
***X*9TH**	38	49	40	38	40	**59**	41	36	37	42	25	25	31	24	32	35	24	40	31	24	30	38	35	30
***X*11**	34	34	37	36	33	34	35	**62**	31	32	39	38	39	39	33	33	31	33	32	40	40	36	33	30
***X*13**	46	45	49	46	49	38	51	34	43	**67**	38	42	38	35	33	45	29	48	26	39	34	40	49	31
***X*14**	38	35	38	41	36	33	37	38	39	38	**77**	60	57	36	34	37	27	38	26	56	38	35	35	34
***X*15**	41	22	42	43	38	25	41	38	40	40	59	**69**	55	38	37	38	27	38	26	53	42	37	35	36
***X*16**	41	34	38	41	39	31	40	36	39	40	56	57	**87**	36	35	38	26	39	30	67	38	36	35	32
***X*17**	35	32	37	38	35	24	36	37	36	36	37	38	35	**70**	34	36	27	34	25	37	47	37	33	31
***X*18**	55	47	53	48	52	36	50	34	48	51	38	40	39	36	35	43	29	50	28	41	34	41	50	33
***X*19**	36	37	36	37	33	34	37	33	35	37	38	38	38	34	**60**	35	34	35	25	40	35	37	37	35
***X*20**	45	41	47	46	44	36	47	33	44	45	39	39	38	36	32	**70**	30	44	25	39	36	35	42	35
***X*21**	30	23	30	38	30	19	31	29	32	29	28	26	24	26	28	29	**59**	29	25	25	27	31	27	28
***X*23**	28	29	24	24	23	33	22	28	24	24	26	27	32	25	22	24	27	25	**60**	32	26	24	22	25
***X*24**	40	34	41	42	40	27	41	39	41	39	54	56	69	36	35	38	26	38	30	**84**	38	35	37	34
***X*25**	36	33	34	37	38	31	35	37	36	34	39	42	40	50	35	32	30	34	29	41	**49**	35	30	31
***X*26**	53	54	55	48	53	51	53	36	53	59	46	44	46	38	38	54	36	53	28	48	38	40	55	39
***X*28**	34	34	34	35	31	30	33	31	33	33	35	37	35	32	35	34	30	31	29	36	32	36	29	**56**
***X*N1**	34	29	35	40	34	27	36	31	35	32	30	31	30	26	33	33	24	35	27	29	27	38	31	30
***X*N2**	56	56	56	54	57	48	56	37	54	57	44	44	47	38	42	54	36	53	33	46	40	41	53	40
***X*N3**	30	21	30	36	28	18	31	29	29	30	26	27	27	23	28	28	52	29	26	25	27	31	29	30
***X*N4**	49	40	51	47	48	35	51	34	49	48	38	39	40	35	32	46	28	50	27	42	34	39	49	31
***X*N5**	47	40	51	44	45	35	50	33	47	48	36	38	39	34	31	45	29	48	27	41	32	40	46	32
***X*N6**	28	30	28	27	29	34	29	30	28	29	28	31	30	25	30	29	26	28	44	32	29	30	27	32

#Comparison of *X. tropicalis *and human MMP protein sequences. Pair-wise comparisons were done to obtain the percent of identities between the MMPs from the two species. H1 ~ 28: human MMP1 ~ 28; C22: chicken MMP22; *X*1 ~ 28, *X*9TH, and *X*N1 ~ N6: *Xenopus tropicalis *MMP1 ~ 28, 9TH, and N1 ~ N6, respectively. The highlighted numbers with bold letters indicate homologies suggesting that *X. tropicalis *and human MMPs are homologs.

### Likely *Xenopus *homologs of human MMPs

The homologs of the rest of human MMPs could not be easily identified based on sequence comparison and phylogenetic analysis. These MMPs may have corresponding homologs in *Xenopus *but their sequences have diverged significantly that it is difficult to match the human and *Xenopus *counterparts. For these MMPs, we kept the putative names for any *Xenopus *MMPs with previously assigned names in the public databases or assigned the names as described below.

#### MMP1

There were two entries for *X. laevis *MMP1 (GenBank accession # BC054233 and BC084836), encoding two closely related MMPs of 466 amino acids (aa) that are 90% identical (data not shown). Alignment of these *X. laevis *MMP1s (MMP1A and MMP1B) to the *X. tropicalis *genomic scaffolds showed significant homology at three different loci on the Scaffold_119 (Note that the *X. tropicalis *genomic sequence is not complete and the individual sequences are assembled into scaffolds instead of individual chromosomes). The putative cDNA sequences were derived from these loci and used to deduce the protein sequences of three related MMPs. Among these three putative MMP genes, the best-matched one has 87% identities with *X. laevis *MMP1s and has the same length; it was therefore named as *X. tropicalis *MMP1 (Table 1). The other two were tentatively named as *X. tropicalis *MMP N4 and N5 (Table 1).

**Figure 2:**
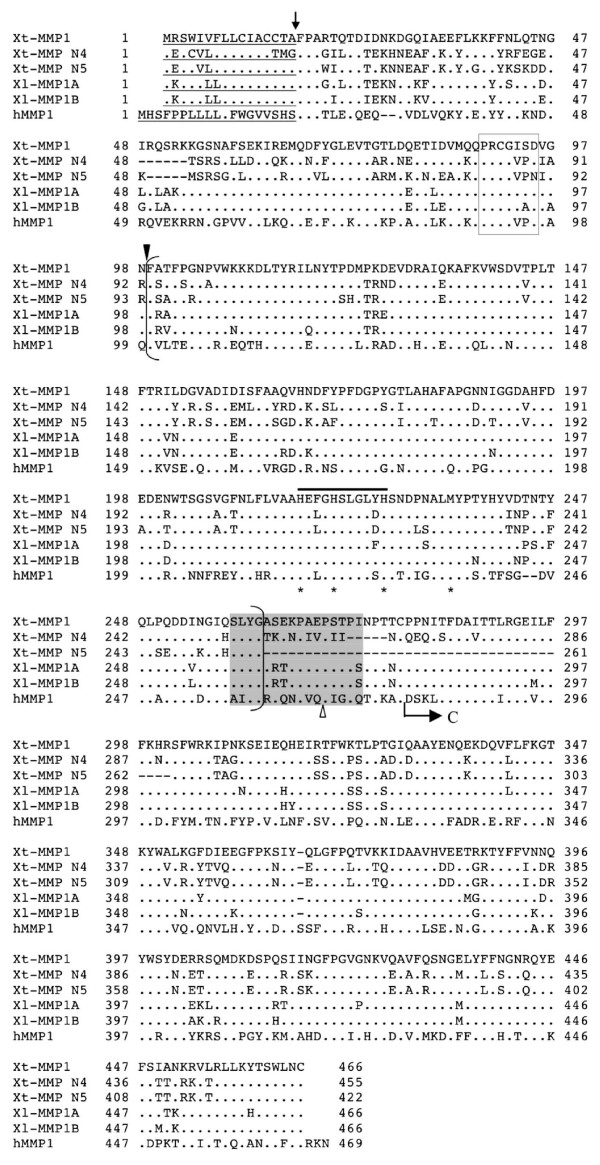
**Sequence comparison of MMP1 with MMP N4 and MMP N5**. *X. tropicalis *(Xt) MMP1, N4 and N5, and *X. laevis *(Xl) MMP1A and 1B were aligned with human (h) MMP1 for comparison. The sequences of the putative signal peptide are underlined. The predicted cleavage site between the signal peptide and the propeptide is indicated by an arrow, and the predicted cleavage site between the propeptide and the catalytic domain is indicated by solid arrowhead. The conserved sequence in the propeptide involved in the "cysteine-switch" is boxed, and the zinc-binding motif within the bracketed catalytic domain is indicated by a solid line on top. The three conserved histidine residues in the zinc binding motif and the conserved methionine residue of the nearby "Met-turn" are indicated by stars below. The 16 aa sequence (shadowed) at the end of the catalytic domain (bracketed) indicates the region whose integrity is important for collagenase specificity for collagen. An insertion of 8 or more aa within this region at the site indicated by an arrowhead is characteristics of stromelysins. The arrow marked "C" shows the beginning of the C-terminal hemopexin-like domain. A dot indicates an identical amino acid as the corresponding one in Xt-MMP1. Gaps (dashes) are introduced to optimize the alignment among proteins. Note that MMP N4 and N5 contain internal deletions in the linker region between the catalytic domain and C-terminal hemopexin-like domain.

Phylogenetic analysis revealed that *X. tropicalis *MMP1, MMP N4 and N5 cluster together with MMP N4 and N5 more closely related to each other than to MMP1 (Fig. 1). These MMPs are related to several human MMP subfamilies including collagenases (MMP1 and 8) and stromelysins (MMP3 and 10), etc. The MMP that is most closely related to these three MMPs is *X. tropicalis *MMP18, a homolog of *X. laevis *MMP18 (Table 1). *X. laevis *MMP18 is a known collagenase [42], suggesting that these three MMPs are collagenases. Apart from the typical MMP domains (i.e., the signal peptide, the conserved zinc binding motif characteristic of MMPs, and the conserved cysteine-switch domain within the propeptide), *X. tropicalis *MMP1, as well as *X. laevis *MMP1A and 1B, contains a 16 aa proline-rich motif after the catalytic domain that distinguishes collagenases from stromelysins, although the *X. tropicalis *MMP N4 and N5 have deletions within the region (*X. tropicalis *MMP N5 lacks the entire hinge domain but has the intact catalytic and hemopexin domain, a characteristic similar to that of the MMP21s) (Fig. 2). These MMPs share less than 60% identity with *X. laevis *collagenases MMP13 and MMP18. In addition, they are also three amino acids shorter than the human MMP1 at the C- terminus (Fig. 2), just like the *X. laevis *MMP13 and MMP18. Taken together, these MMP genes are likely *X. tropicalis *collagenases, but it is possible that the *X. tropicalis *MMP1 is not the true homolog of human MMP1, especially considering that the MMP1 from another amphibian species, *Rana catesbeiana*, is much more homologous to human MMP1, although much shorter, compared to *Xenopus *MMP1 (Fig. 1) [42].

#### MMP3

**Figure 3:**
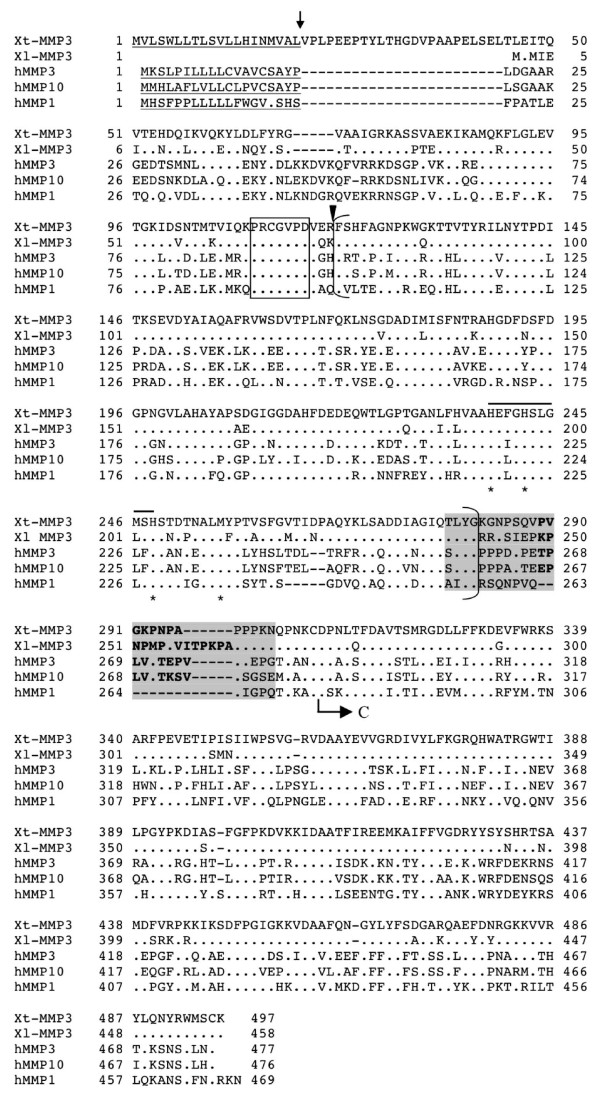
**Comparison of *Xenopus *MMP3 with human MMP1, 3, and 10**. Note that the shadowed region at the end of the catalytic domain corresponds to the same region in Fig. 2 except the insertion of 8–14 aa in stromelysins (MMP3, 10) compared to collagenases (e.g., the MMP1 shown here). See Fig. 2 for other information.

MMP3 and MMP10, also referred to as stromelysin 1 and 2, respectively, are MMPs that have quite broad substrate specificities and were originally described as proteoglycanases [59-62]. Neither *Xenopus *MMP3 nor MMP10 has been characterized. A putative MMP deduced from a cDNA entry for each *Xenopus *species (GenBank accession number: BC077966 of *X. laevis *clone and NM_001030331 of *X. tropicalis *clone) structurally resembles MMP3 and MMP10 (Fig. 3). Similar to human MMP3 and MMP10, the *Xenopus *MMP has an insertion (8 aa for the *X. tropicalis *MMP and 14 aa for the *X. laevis *MMP) in the 16 aa proline-rich motif after the catalytic domain whose integrity is important for collagenase activity (Fig. 3). This suggests that the *Xenopus *MMP is likely a stromelysin. The two *Xenopus *homologs share 84% identity, although the *X. laevis *one is 45 aa shorter than *X. tropicalis *MMP3 at the N-terminus, likely due to incomplete 5'-end cDNA sequence. Thus, the *Xenopus *MMPs are homologs of each other and are tentatively named as MMP3 since they are slightly more similar to human MMP3 than MMP10 (Table 2).

#### MMP23

**Figure 4:**
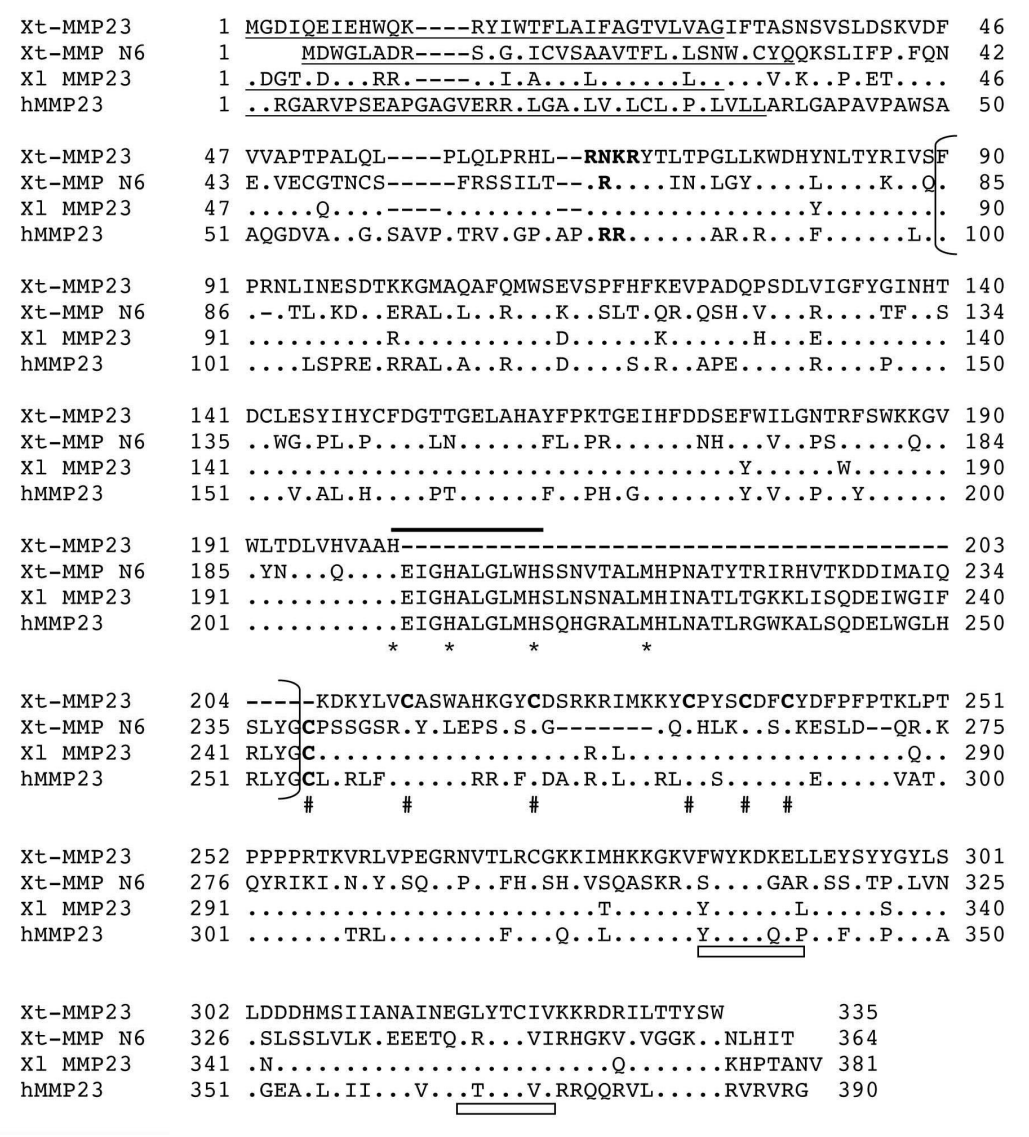
**Comparison of frog and human MMP23 with MMP N6**. The predicted signal anchor (transmembrane domain) sequences are underlined and the putative furin recognition sequences are in bold. The cysteine residues in the "cysteine-array" unique to MMP23 are in bold and indicated with # below. The amino acid residues characteristic of an Ig (immunoglobulin)-fold are indicated with rectangle boxes below. See Fig. 2 for other information.

MMP23 is characterized by the presence of a furin activation site, a type II transmembrane domain at the N-terminus, and a unique truncated C-terminal domain unrelated to the hemopexin domain found in other MMPs, and it lacks a typical prepeptide [63-66]. There are two reported MMP23, MMP23A and MMP23B, in human that are encoded by two genes, likely due to a very recent, partial duplication at Chromosome 1p36.3 [65]. Human MMP23A and B are identical in amino acid sequences and thus both are referred to as MMP23 here. Two overlapping EST entries (CD302225 and CD302813) encode a putative *X. laevis *MMP23 of 381aa. Sequence search of the *X. tropicalis *genome identified a putative *X. tropicalis *MMP23 on Scaffold_414. There was no EST entry representing the *X. tropicalis *MMP23. However, two *X. tropicalis *EST entries (CX344815 and CX344816) composed of cDNA sequences that together encode another putative MMP related to MMP23 (tentatively named as MMP N6). These cDNA sequences aligned on to *X. tropicalis *genomic Scaffold_132. *X. tropicalis *MMP23 and MMP N6 differ from each other at both the cDNA and protein sequence levels, unlike the two human MMP23 genes (note that there is only one MMP23 in the mouse genome). Sequence comparison showed that *X. laevis *MMP23, *X. tropicalis *MMP23 and MMP N6 have the same features of human MMP23, although there is a sequence gap in *X. tropicalis *MMP23 (Fig. 4), possibly due to incomplete genomic sequence information (see Additional file 1). The *Xenopus *MMP 23, MMP N6, and human MMP23 cluster together and are away from all other MMPs (Fig. 1) with the MMP23 sharing 60% identity between *Xenopus *and human, similar to other homologous MMPs (table 2).

#### MMP21

**Figure 5:**
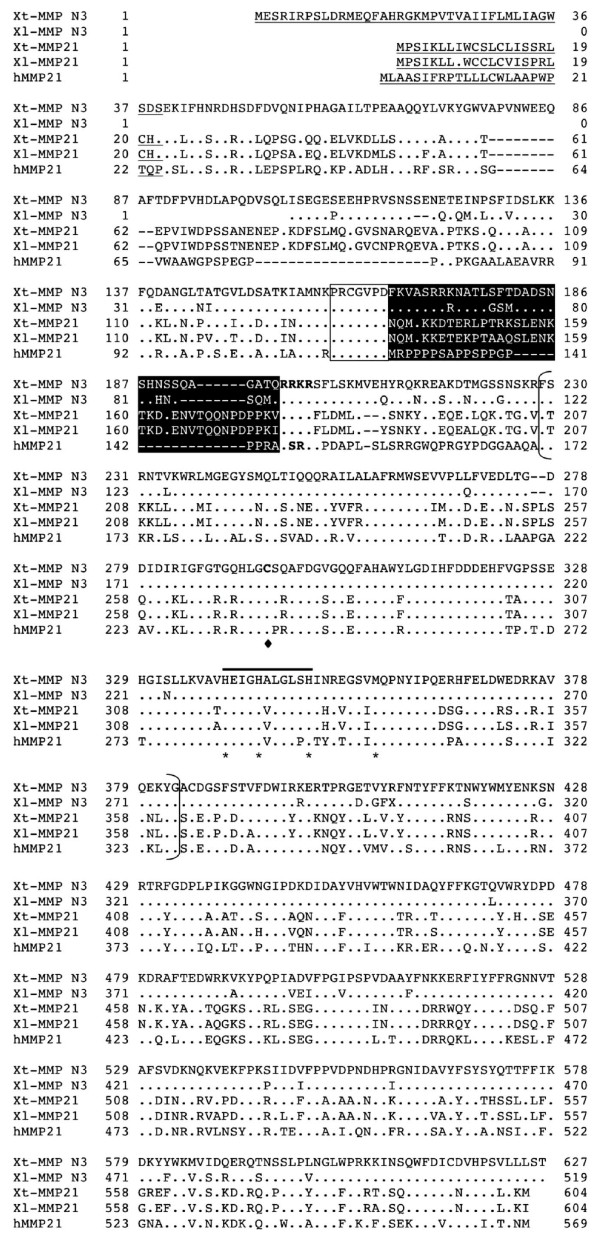
**Comparison of MMP21 with MMP N3**. The predicted signal peptide is underlined and the putative furin recognition sequences within the propeptide are in bold. The sequence in white on dark background indicates the unique insertion in the propeptide in MMP21. A unique cysteine residue in the catalytic domain is in bold and indicated with a black diamond below. Note that the sequence for Xl-MMP N3 is incomplete at the N-terminus. See Fig. 2 for other information.

MMP21 was first cloned in *X. laevis *[67]. It has since been found to be present in other vertebrates including human. The common features of this MMP across different species, in addition to those characteristics of MMPs, are a putative furin cleavage site between the propetide and the catalytic domain, a relative long insertion (20~44 aa) between the PRCGXPD cysteine switch motif and the furin cleavage site (RXKR), and a unique cysteine residue in the catalytic domain (Fig. 5). The putative *X. tropicalis *MMP21 composes of 604 aa and shares 92% and 59% identities with the *X. laevis *and human MMP21, respectively (Tables 1 and 2).

Interestingly, sequence search also revealed a cDNA entry (TC84199), composed of *X. tropicalis *ESTs DR836290 and CX386748, that encodes a putative MMP of 524 aa. This MMP is highly homologous to *X. tropicalis *MMP21 and is tentatively named as *X. tropicalis *MMP N3 (Fig. 5). In addition, 4 highly homologous *X. laevis *ESTs, BG234242, BU905338, CB558404, and CF547511, were also found to constitute a putative cDNA sequence encoding a homolog of *X. tropicalis *MMP N3 (see Additional file 1, Table 1, and Fig. 5). The *X. tropicalis *MMP N3 is located on the *X. tropicalis *genomic Scaffold_508, from which the rest 5'-end cDNA sequence was predicted. The putative *X. tropicalis *MMP N3 encodes a predicted protein of 627 aa. The N-terminal sequence of *X. laevis *MMP N3 is still missing in the databases. Sequence comparison clearly showed that MMP N3 is highly homologous to MMP21 and is likely derived from a gene duplication event (Fig. 5). Furthermore, phylogenetic analysis indicated that *Xenopus *MMP21 clusters with human MMP21 and is closely related to MMP N3.

#### MMP7 and 26

**Figure 6:**
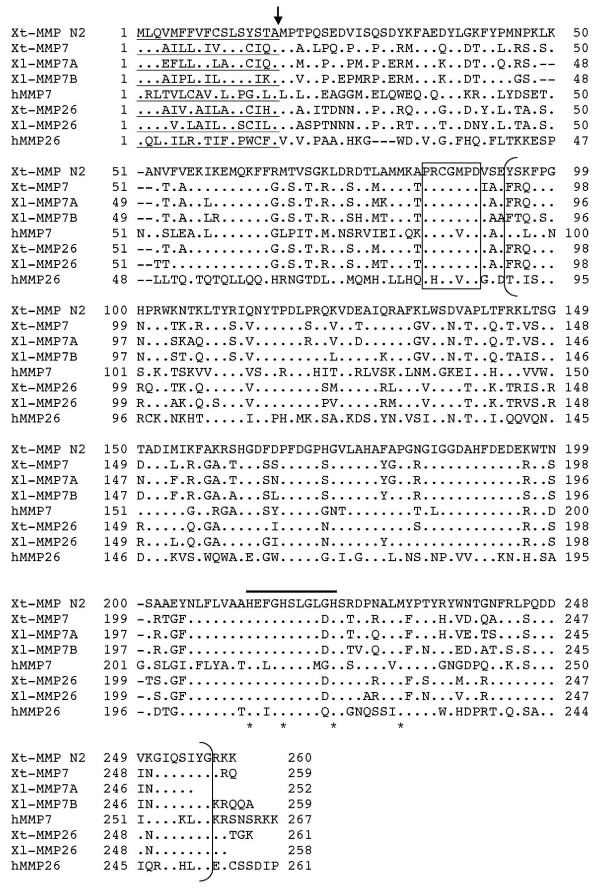
**Comparison of MMP N2 with MMP7 and MMP26**. Note that like human and *Xenopus *MMP7 and MMP26, MMP N2 lacks the linker peptide and hemopexin-like domain at the C-terminal. See Fig. 2 for other information.

MMP7 and MMP26 are the smallest MMPs known and have no hemopexin domain at their C-terminus [62,68-70]. *X. laevis *MMP7 was previously reported (GenBank accession # AF573380) and found to be expressed specifically in tissue resident macrophages [56]. Bioinformatic search revealed a closely related *X. laevis *cDNA clone (GenBank accession # BC056040) encoding a protein of 259 aa vs. 252 aa for *X. laevis *MMP7. These two *X. laevis *MMPs shared 85% identities in amino acid sequences and 82% identities in nucleotide sequences, and therefore, are likely duplicated MMP7 genes in the pseudotetraploid genome. We designated them as MMP7A (GenBank accession # AF573380) and 7B (GenBank accession # BC056040), respectively. Both *X. laevis *MMP7A and 7B aligned to *X. tropicalis *Scaffold_119 at the location that encodes a *X. tropicalis *cDNA (GenBank accession # NM_001005043). Thus, this *X. tropicalis c*DNA represents *X. tropicalis *MMP7. *X. tropicalis *MMP7 is 259 aa in length and highly homologous to *X. laevis *MMP7A/B (Tables 1 and Fig. 6).

In addition to the MMP7 genes, the *X. laevis *cDNA clone MGC69070 (GenBank accession # BC056080) and *X. tropicalis *cDNA clone MGC108008 (GenBank accession # NM_001032335) also encode a small MMP each (258 aa and 261 aa, respectively). Like MMP7, both proteins lacked the hemopexin domain (Fig. 6). They share 88% identities with each other and the *X. tropicalis *gene is also located on *X. tropicalis *genomic Scaffold_119. These two proteins are closely related to *X. laevis *MMP7A/B and *X. tropicalis *MMP7 on the phylogenetic tree and thus are tentatively named as *X. laevis *MMP26 and *X. tropicalis *MMP26, respectively. (It should be pointed out that it is difficult to assign with certainty which of the *Xenopus *gene is the homolog of human MMP7 and which is that of human MMP26. For consistency, we kept *Xenopus *MMP7 for the previously published sequence [56]).

Surprisingly, an additional *X. tropicalis *clone (IMAGE7719439, EST# CX982585 and CX982586) was also found to encode a similar MMP lacking the hemopexin domain. The cDNA sequence had two in-frame stop codons after the 3'-end of the coding sequence (see Additional file 1) and another independent EST sequence (EST #CX979196) overlapped with this region with 100% identity (data not shown). Thus, this gene represents a novel MMP that is structurally similar to MMP7 and MMP26. It is tentatively named as *X. tropicalis *MMP N2 (Fig. 6). *X tropicalis *MMP N2 is also located on *X. tropicalis *Scaffold_119 in between MMP7 and MMP26, suggesting that it was derived from a gene duplication event.

### Novel *Xenopus *MMPs

#### Gelatinases

**Figure 7:**
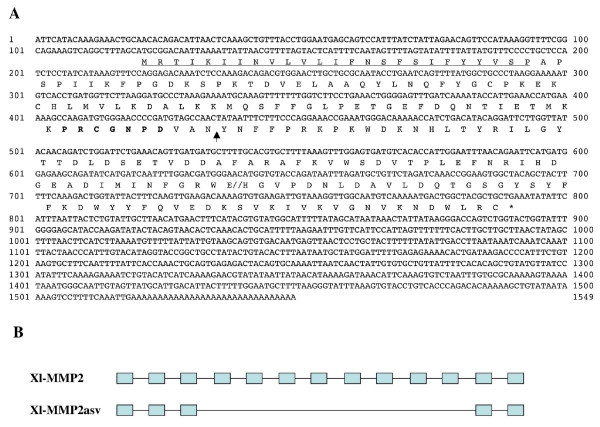
**Putative alternative splicing variant of *X. laevis *MMP2 (MMP2asv)**. A) Nucleotide and deduced amino acid sequences of MMP2asv. The protein contains, from the N-terminus to C-terminus, a signal peptide (underlined), the conserved sequence in the propeptide involved in the "cysteine-switch" (in bold letters), a truncated catalytic domain linked to a truncated hemopexin domain (separated by double slash lines). The predicted cleavage site between the propeptide and the catalytic domain is indicated by an arrow. B) Comparison of the full length and alternatively spliced *X. laevis *MMP2 exon/intron organization. Solid blocks stand for exons present in the mRNAs and lines are introns.

Sequence analysis revealed the existence of an alternatively spliced form of *X. laevis *gelatinase A (MMP2) (MMP2asv, Fig. 7). This alternatively spliced MMP2 transcript encodes a MMP that lacks most of the catalytic domain, including the zinc binding motif, and part of the C-terminal hemopexin domain. To date, no such spliced form of MMP2 has been reported for other vertebrate species, including *X. tropicalis*.

In addition, there are two MMPs in both *X. laevis *and *X. tropicalis *that are highly homologous to MMP9 or gelatinase B, MMP9 and MMP9TH, respectively [43] (Tables 1 and 2), and both cluster with human MMP9 in the phylogenetic tree (Fig. 1). *Xenopus *MMP9 and MMP9TH have all the features characteristic of a gelatinase (data not shown). Since only one MMP9 genes have been found in other vertebrates, MMP9TH and MMP9 represent a unique duplication in *Xenopus*.

#### MMP N1

**Figure 8:**
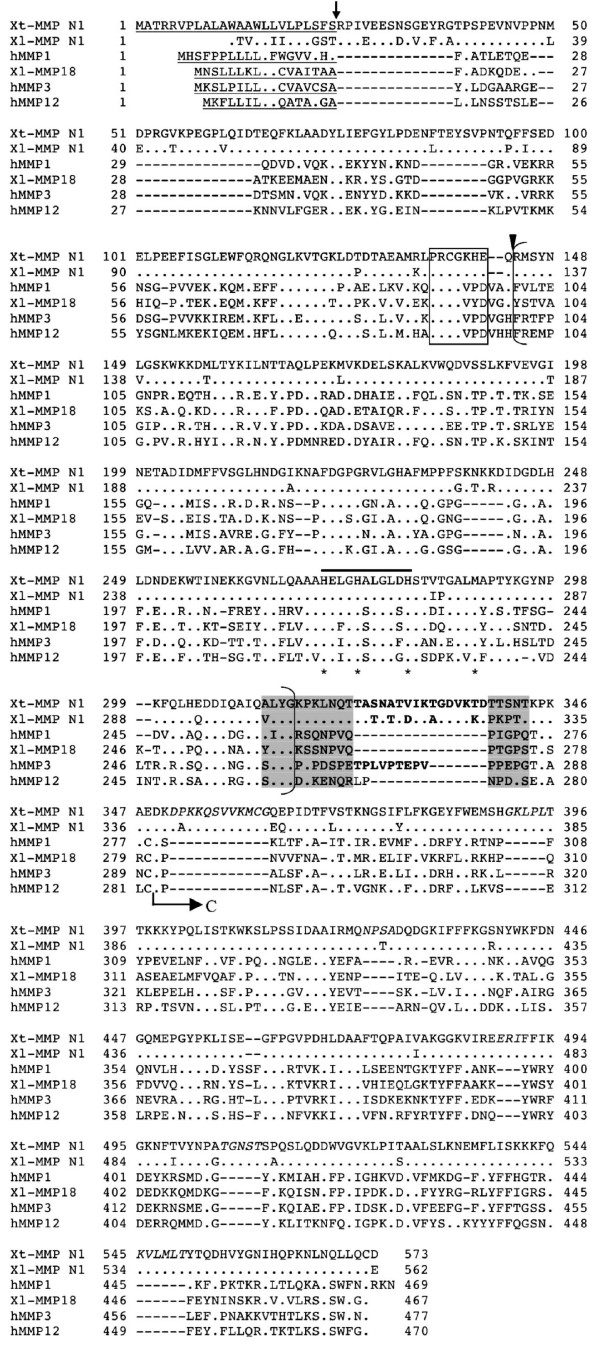
**Comparison of *Xenopus *MMP N1 with human MMP1, 3, 12, and *X. laevis *MMP18**. The amino acid sequence in shadowed letters corresponds to the region equivalent of the proline-rich sequences (16 aa) at the end of the catalytic domain in human MMP1 whose integrity is important for the collagenase specificity for collagen. A short peptide insertion (in bold letters) within this region is characteristics of stromelysins as shown here for MMP3. The *Xenopus *MMP N1 has a 16 aa-insertion within the same region (in bold letters) as well as some additional insertions within the C-terminal hemopexin-like domain (in italics). See Fig. 2 for other information.

The assembly of a group of overlapping *X. laevis *ESTs (EST # BE509380, BJ032306, BG813136, EB469763, EC276067, BX852582, BJ047339, and BG578455) led to a cDNA (see Additional file 1) encoding a protein of 562 aa that lacks the N-terminus and shares low levels of homologies with others MMPs but has all the characteristics of an MMP (Fig. 8 and data not shown). Similarly, the homologous *X. tropicalis *ESTs could be assembled into a cDNA encoding a full length MMP of 573 aa that is 90% homologous to the *X. laevis *counterpart (Fig. 8 and table 1). This *X. tropicalis *MMP has less than 40% homology to any of human MMPs and represents a novel frog MMP tentatively named as *X. tropicalis *MMP N1 (Fig. 8 and Table 2). Its counterpart in *X. laevis *is therefore named as *X. laevis *MMP N1 (Fig. 8 and Table 1).

While the *Xenopus *MMP N1 has all the domains typical of an MMP, there are some differences distinguishing it from other MMPs. First, both *X. laevis *and *X. tropicalis *MMP N1 had a "cysteine-switch" made of "PRCGKHE" instead of the conserved "PRCGXXD" sequence and a deletion of two amino acid residues around the predicted cleavage site for the mature MMPs (Fig. 8). Second, these two MMPs are most similar to collagenases and stromelysins in their domain organization but have a 16 aa insertion in the 16 aa collagen-binding motif of collagenases at the exact position where an insertion is found for stromelysins (Fig. 8). Finally, there are a number of insertions throughout *Xenopus *MMP N1 compared to human collagenases and stromelysins (Fig. 8). These unique features suggest that *Xenopus *MMP N1 is a novel MMP distinct from other known MMPs.

#### MMP N2

**Figure 9:**
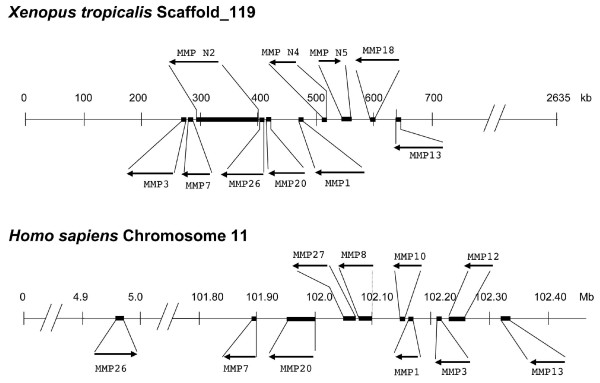
**Comparison of the MMPs cluster on *X. tropicalis *Scaffold_119 to that on human Chromosome 11**. *X. tropicalis *MMP cDNA sequences were used to do BLAST search against the *X. tropicalis *genomic sequences to locate the genes on the assembly scaffolds. MMP 1, 3, 7, 13, 18, 20, 26, as well as the novel ones MMP N2, N4 and N5 are found on Scaffold_119. They were arranged on the scaffold according to their location and orientation. The human MMPs on Chromosome 11 were arranged according to the annotations for their locations and orientations in Human Genome Build 36.3 on the NCBI website. The MMPs shown above the line for the chromosome/scaffold are MMPs specific to *X. tropicalis *or human while those shown below are the MMPs present in both species. Mb, mega base pair; kb, kilo base pairs. Note the gene size was not drawn to scale for clarity.

As described above, there are three *X. tropicalis *MMPs homologous to human MMP7 and MMP26. Sequence analyses allowed us to assign two of them as *X. tropicalis *MMP7 and MMP26, respectively. The third one thus represents a novel gene of this subfamily (Figs. 1 and 6). Interestingly, all three *X. tropicalis *MMP genes are located consecutively on a single chromosome with MMPN2 in between MMP7 and MMP26 (Fig. 9), suggesting that a duplication event in amphibians led to the extra MMP in the subfamily.

#### MMP N3

MMP N3 is one of the two genes in both *X. laevis *and *X. tropicalis *that have similar levels of homology to human MMP21 (Figs. 1 and 5). Although MMP N3 and MMP21 are located in different scaffolds in *X. tropicalis *genomic sequence, it is possible that they are located adjacent to each other in a chromosome as the genomic sequence annotation is incomplete in *X. tropicalis*. Thus, these two genes might have derived from a unique gene duplication event in amphibians.

#### Collagenases

Based on sequence features and its enzymatic activity, *X. laevis *MMP18 was proposed to be a novel collagenase [42]. Indeed, potential homologs of mammalian collagenases MMP1 and MMP13 have been reported for *Xenopus*. Interestingly, while the amphibian MMP13 and human MMP13 are 67% identical, the corresponding MMP1s are only 51% identical (Table 2), similar to the levels of homology that MMP18 has with human collagenases (MMP1, 8, and 13). Furthermore, human MMP1 and MMP8 have 3 extra amino acids (RKN) at the C-terminus that are lacking in *Xenopus *MMP1 and MMP18 as well as the putative, novel *Xenopus *collagenases (MMP N4 and N5) (Fig. 2). In addition, a most likely homolog of human MMP1 has been reported for another amphibian species, *Rana catesbeiana *[40]. The *Rana *MMP1 is much shorter but much more homologous to human MMP1 (about 80%), compared to these *Xenopus *collagenases (data not shown and [42]). Furthermore, unlike the *Xenopus *MMP1, the *Rana *MMP1 clusters with human MMP1 on the phylogenetic tree (Fig. 1). Thus, it is possible that MMP1 has diverged extensively between amphibians and mammals, leading to a very different size in *Rana *or its loss in *Xenopus*. On the other hand, all known and putative collagenases in *Xenopus *are located consecutively in a single chromosome in the order of MMP1, MMP N4, MMP N5, MMP18, and MMP13 (Fig. 9). This suggests that multiple duplication events might be responsible for the generation of these MMPs. It is interesting to note that human MMP 1, 3, 7, 8, 10, 12, 13, 20, 26 and 27 are all encoded by Chromosome 11 (Fig. 9). With the exception of four MMPs (MMP8, 10, 12 and 27) that have no apparent homologs in *X. tropicalis*, the other six MMPs found on human Chromosome 11 are clustered on the Scaffold_119 together with four frog-specific ones (MMP18, MMP N2, N4, and N5) in *X. tropicalis *(Fig. 9). Thus, *X. tropicalis *Scaffold_119 appears to contain a large region syntenic to human Chromosome 11 [71]. On the other hand, with the exception of MMP13 and MMP20, it is difficult to determine which MMP in this cluster of 10 *X. tropicalis *MMPs is the homolog of an MMP in the human cluster based on sequence homology and syntenic analyses. *X. tropicalis *MMP13 and MMP20 share high degrees of homology (about 70%) with their human counterparts, supporting that they are true homologs of the human MMPs. Consistently, phylogenetic analysis of *X. tropicalis *and human MMPs showed that these two MMPs evolved earlier than the other MMPs in the cluster (see Additional file 2). In addition, the drastic differences in the distances between MMP7 and MMP26 on the chromosome (100 mega bp in human and 100 kb in *Xenopus*) (Fig. 9) and on the phylogenetic trees (Supplemental Fig. 2), suggest that this duplication occurred after the separation of amphibians from mammals. The other six MMPs in the cluster appear to have diverged rapidly during evolution and/or evolved through duplications and/or losses independently in amphibians and mammals.

## Conclusion

Through a bioinformatic approach, we have identified *Xenopus *homologs for most human MMPs. By comparing the MMPs in the two highly related species, *X. tropicalis *and *X. laevis*, we have been able to discover several unique duplications of MMPs genes in amphibians that are absent in mammals. On the other hand, several human MMPs have no apparent homologs in *Xenopus *and were possibly evolved *de novo *in mammals. Among the likely duplicated genes, genes in the following two pairs, MMP9 and MMP9TH, MMP21 and MMP N3, MMP 7 (or MMP26) and MMP N2, are more homologous to each other than to their human homologs (Fig. 1), suggesting that the duplications occurred after the separation of amphibians from mammals. On the other hand, MMP23 clusters closer to its human homolog than to the putative duplicate MMP N6 (Fig. 1), suggesting the possibility that MMP23 might have duplicated before the separation of amphibians and mammals and one copy was lost subsequently in mammals. Duplications and loss in MMP genes are also evident when comparing the largest MMP cluster located on human Chromosome 11 with the MMP cluster on *X. tropicalis *Scaffold_119, where a number of novel MMPs were found and several MMPs were lost in *X. tropicalis*. Our findings thus demonstrate a dynamic process for MMP gene evolution. It will be of interest in the future to investigate whether MMP expression and function are conserved during vertebrate development. The sequence information reported here and the advantage of the amphibian metamorphosis for functional studies *in vivo *should facilitate such an endeavor in the near future.

## Methods

We first searched the public EST database at the National Center for Biotechnology Information (NCBI, ) and the Gene Index Project in Computational Biology and Function Genomics Laboratory  with known *Xenopus *MMP genes for other possible MMP sequences based on sequence similarities. The identities of putative *Xenopus *MMPs were tentatively determined by building a phylogenetic tree with human MMPs. This was done through Multiple Sequence Alignment by CLUSTALW . Human MMPs used were: MMP1 (NP_002412), MMP2 (NP_004521), MMP3 (NP_002413), MMP7 (NP_002414), MMP8 (NP_002415), MMP9 (NP_004985), MMP10 (NP_002416), MMP11 (NP_005931), MMP12 (NP_002417), MMP13 (NP_002418), MMP14 (NP_004986), MMP15 (NP_002419), MMP16 (NP_005932), MMP17 (NP_057239), MMP19 (NP_002420), MMP20 (NP_004762), MMP21 (NP_671724), MMP22 (NP_990331), MMP23 (NP_008914), MMP24 (NP_006681), MMP25 (NP_071913), MMP26 (NP_068573), MMP27 (NP_071405), and MMP28 (NP_077278). The *X. laevis *MMPs were: MMP1A (BC054233), MMP1B (BC084836), MMP2 (AY037943), MMP3 (BC077966), MMP7A (AY573380), MMP7B (BC056040), MMP9 (AF072455), MMP9TH (AB288054), MMP11 (Z27093), MMP13 (L49412), MMP13A (U41824), MMP14A (AY633953), MMP14B (BC077870), MMP15 (AY573378), MMP16 (AY310397), MMP17 (CK806816), MMP18 (L76275), MMP19 (BX847184), MMP20 (DQ885892), MMP21 (U82541), MMP23 (CD302225 &CD302813), MMP24A (CA791076 &EB480268), MMP24B (EB483310), MMP25 (BC078136), MMP26 (BC056080), MMP28A (EF187277), MMP28B (BC061659), MMP N1 (Assembly of BJ032306, BE509380, EC276067, BX852582, BJ047339 and BG578455) and MMP N3 (Assembly of BG234242, BU905338, CB558404, and CF547511) (See Supplemental Fig. 1). Pair-wise comparison of protein sequences was conducted by using MacVector (Accelrys Inc., San Diego, CA) to further confirm the identity assignment. X. *laevis *or *tropicalis *cDNA sequences were used to do BLAST search against the *X. tropicalis *genome assembly 4.1  to determine the corresponding gene structures and predict cDNA sequences if necessary. The *X. tropicalis *MMPs thus obtained from the GenBank database were: MMP2 (NM_001015789), MMP3 (NM_001030331), MMP7 (NM_001005043), MMP9 (NM_001006842), MMP14 (NM_001030388), MMP15 (NM_001015921), MMP16 (NM_001015992), MMP17 (NM_001102999), MMP18 (NM_001030330), MMP19 (BC153750), MMP25 (CU075461), MMP26 (NM_001032335), MMP N1 (BC155487, DN028798, DN034177 and DN076875), MMP N2 (CX982585, CX982586 and CX979196), MMP N6 (CX344816 and CX344815). Other *X. tropicalis *MMP sequences were derived from predicted exons of the genomic sequences (see Additional file 1 and Table 1 for their sequences and locations in the genome).

## Abbreviations

MMP: matrix metalloproteinase; ECM: extracellular matrix; TH: thyroid hormone; ST3: stromelysin 3; EST: expressed sequence tags; aa: amino acid; bp: base pair.

## Authors' contributions

LF collected sequence information, performed bioinformatic analysis and wrote the first draft; BD and SM performed bioinformatic analysis and edited the manuscript; and YS supervised the research and finalized the paper. All the authors critically revised and approved the final version of the paper.

## Supplementary Material

Additional file 1 **The nucleotide sequences of the *X. tropicalis *and *X. laevis *MMPs.** The data presented all the all the nucleotide sequences of the *X. tropicalis *and *X. laevis *MMPs that were used for deducing Xenopus MMPs in the study. GenBank accession numbers or the scaffold of the *X. tropicalis *genome on which the *Xenopus *MMP locates were included if applicable. Click here for file

Additional file 2 **Phylogenetic trees of *X. tropicalis *and human MMPs. ***X. tropicalis *MMPs along with *Rana catesbeiana *MMP1 (RcMMP1) or human MMPs along with chicken MMP22 (CMMP22) were analyzed using the multiple sequence alignment program CLUSTALW to generate the corresponding phylogenetic trees with defined ancestral nodes marked by purple square. The MMPs located on human Chromosome 11 and those located on *X. tropicalis *Scaffold_119 are in red. Click here for file
