# Supplementary material for: Genome-wide identification of Xenopus matrix metalloproteinases: conservation and unique duplications in amphibians
Source: BMC Genomics. 2009 Feb 17;10:81. doi: 10.1186/1471-2164-10-81 (PMC2656525; doi:10.1186/1471-2164-10-81)
Supplement: Additional file 1 — The nucleotide sequences of the X. tropicalis and X. laevis MMPs. The data presented all the all the nucleotide sequences of the X. tropicalis and X. laevis MMPs that were used for deducing Xenopus MMPs in the study. GenBank accession numbers or the scaffold of the X. tropicalis genome on which the Xenopus MMP locates were included if applicable. [file 1471-2164-10-81-S1.doc]

***X. tropicalis* MMPs cDNA sequences:**

**1. Xt-MMP1 cDNA (Scaffold_119):** AGAGCAAGGTTGGAAGCTTAACCTTTGAGGCAATAAGCTCCAAGAAACAGCTACTTAGCCACGAGCAGCA**ATG**AGGTCCTGGATTGTGTTCCTGCTGTGTATAGCATGTTGCACGGCATTCCCTGCTAGGACCCAGACAGACATAGATAACAAAGATGGACAGATTGCTGAGGAGTTCTTGAAAAAATTCTTCAACCTTCAGACAAATGGGATCCGCCAGTCAAGAAAGAAGGGCAGCAATGCATTCTCCGAGAAAATCCGTGAGATGCAGGACTTCTATGGTTTGGAGGTCACTGGAACACTGGATCAGGAAACCATAGATGTGATGCAACAACCTCGGTGTGGGATTTCTGACGTCGGCAATTTTGCGACCTTCCCTGGAAACCCTGTTTGGAAGAAGAAAGATCTGACATACAGAATACTAAACTACACCCCAGATATGCCCAAAGATGAGGTGGACCGGGCCATTCAGAAGGCTTTCAAGGTCTGGAGTGATGTTACCCCACTTACCTTTACTAGAATCCTTGATGGTGTCGCTGATATTGATATCTCATTTGCAGCTCAAGTTCATAATGATTTTTATCCTTTTGATGGCCCATATGGTACCCTGGCCCATGCATTCGCTCCTGGAAACAACATTGGAGGGGATGCACATTTTGACGAAGATGAGAACTGGACTAGTGGATCAGTTGGTTTCAACTTGTTCCTTGTGGCTGCTCACGAGTTTGGCCATTCCCTTGGGCTCTACCATTCTAATGACCCTAATGCCCTTATGTACCCAACCTACCATTACGTCGATACCAATACATACCAGCTGCCTCAGGATGACATCAATGGAATCCAGTCACTGTATGGAGCAAGTGAAAAGCCCGCTGAACCATCAACACCAATCAACCCAACAACATGCCCCCCAAACATAACATTTGATGCTATAACAACACTGCGTGGAGAAATCTTGTTCTTTAAACACAGATCCTTCTGGCGCAAGATCCCCAATAAATCAGAAATTGAGCAGCATGAGATCAGAACATTCTGGAAAACTCTTCCCACTGGAATTCAGGCAGCCTATGAAAATCAGGAAAAGGATCAGGTTTTTCTCTTTAAAGGAACCAAATACTGGGCTCTTAAAGGCTTCGACATTGAGGAGGGATTCCCTAAGAGCATCTACCAACTTGGGTTTCCCCAGACTGTAAAGAAAATCGATGCAGCTGTTCATGTTGAAGAAACAAGGAAAACCTACTTCTTTGTGAATAATCAGTATTGGAGCTATGATGAAAGAAGATCACAAATGGATAAAGACTCTCCTCAGAGCATCATTAATGGATTCCCAGGTGTCGGAAACAAGGTGCAAGCAGTTTTCCAATCAAATGGTGAGCTCTATTTCTTCAATGGCAATCGGCAGTATGAATTCAGCATAGCCAATAAGAGGGTCTTGCGCCTGCTTAAATACACCAGTTGGTTGAACTGCTAATC

**2. Xt-MMP2 cDNA (NM_001015789):**

GTTTTCGGCAGATACTCAGGCTGCATC**ATG**CGGACTATTAATGTTCATGTTTTAGTCTTCATTTTCAAAA

GTCTCAGTACATTTTATTATGTTTCTTCTGCACCATCTCCTATTATAAAGTTTCCAGGAGACAAATCTCC

AAAGTCAGACGTTGAATTTGCTGCGCAATACCTAAATCAATTTTATGGCTGCCCTAAGGATAAATGTCAC

ATGATGGTTCTTAAGGATGCCCTTAAGAAAATGCAAAAATTCTTTGGTCTTACTGAAACTGGGGAGTTTG

ATCAAAATACCATTGAAACCATGAAAAAGCCAAGATGTGGAAACCCTGATGTAGCCAACTACAACTTCTT

CCCCAGAAAACCCAAATGGGACAAAAATCAGCTGACATACAGGATTATTGGATACACAACAGATTTGGAT

TCTGAAACAGTTGATGATGCTTTTGCACGTGCTTTTAAAGTTTGGAGTGATGTCACACCACTGGAGTTTA

CCCGAATTCATGATGGAGAAGCAGATATCATGATCAATTTTGGACGATGGGAACATGGTGATGGATATCC

ATTTGATGGCAAAGATGGACTTCTGGCTCATGCATTTGCACCTGGATCAGGTGTTGGAGGAGACTCGCAT

TTTGACGATGATGAGCTTTGGACGTTAGGAGAAGGCCAAGTTGTGAAAGTAAAGTATGGGAACGCAGATG

GGGAGTTCTGTAAGTTCCCTTTCATGTTTAATGATAAGGAATACAACAGCTGTACTGATTCAGGTCGGTC

AGATGGCTTCCTTTGGTGCTCTACTACATATGATTTTGACAAGGATGGGAAATACGGCTTCTGTCCACAT

GAGTTGCTATTTACCTTAGCTGGTAATGCAGAAGGGAAGCCCTGCAAATTCCCTTTTAAGTTTCAAGGCA

GCACATTTGACAGCTGCACCACTGAAGGGCGAACAGATGGCTACAGGTGGTGTGCTACTACAGAAGACTA

CGACAAAGATAAGCTGTATGGCTTCTGTCCAGAAACAGCTTTATCCACTGTTGGAGGAAATTCTGAGGGT

TCACCATGTGTCTTTCCTTTCACCTTTCTGGGAAACAAGTACGACTCATGCACTAGCTCTGGCCGCAGTG

ATGGGAAACTATGGTGTGCTTCAAGCAGTAACTATGATGATGATCGCAAGTGGGGGTTCTGTCCTGATCA

AGGTTACAGTCTTTTCCTAGTAGCTGCTCATGAATTTGGGCATGCTCTAGGGCTAGAGCACTCTCAAGAT

CCTGGAGCATTAATGGCTCCAATTTATACATTCATAAAAAACTTTCGGCTTTCACAAGATGATATTACGG

GGATTCAAGAACTTTATGGTCAGGGATCCAAGGAAAAACCACGTCCTGGACCTGTCCCTACAATGGGCCC

TGTCACACCAGATCTGTGCTCTAAAGATGTTGTACTTGATGCCATGTCTCAAATAAGAGGGGAAACATTC

TTTTTCAAAGACAGATTTATATGGCGCACTCCAAACCCAAGAAATAAACCAACAGGACCTCTACTTGTTG

CTACTTTCTGGCCTGAGCTACCTGATAAGATTGATGCTGTTTATGAAGAACCTCAAGAAGAGAAGACTGT

TTTCTTTGCAGGTAATGAGTATTGGGTCTACTTATCAAGCACTTTGGAAAGAGGATACCCGAAAAAGTTG

ACCAGTTTAGGACTACCTCCTGATGTTGATCGTGTAGATGCTGCATTCAACTGGAGCAAAAACAAAAAGA

CATACTTCTTTACTGGGGACAAGTTCTGGAGATATAATGAAGTAAAAAAGAAAATGGACATTGGTTTCCC

CAAATTAATTGCAGATGCATGGAATGGTGTACCAGATAATTTAGATGCTGTGCTGGATCAAACCGGAAGT

GGGTACAGCTACTTTTTCAAAGACTGGTATTACTTTCAAGTTGAAGACAAAAGTGTGAAGATTGTAAAGG

TTGGAAATGTCAAAAATGACTGGCTACGCTGCTGAAATGTATTCATTTAGCTACGCTGTATTCCTTAACA

TGACCTTTCATACTTGTATGGCTTTTTTTGTTATAGCATAATAAAGTATTATAATGGACCAGTCTGGTAC

TGGTATCTGAGGAGCTTAGGAAGATATGCTACAGTAACACTCAAACACTGCAATATTAAGAATTTGTACA

TTCCGCTAGAATTTTTCACTTGCTCCAATTAACTATATTAACTTCAGCTTAAAATGTTTTGTATTATAGT

AAGCAGCACTGCCATACGTTTTTGGATCAGTTTAATGTTCAATAACCAGGGAAGGAACATGCTGCTTAAT

CCTTTTCCTTAATGAATCATCCACTAACCCATTTGTATATAGGCACCGGCTGCCTATACATAATTGTAAC

ATTATGGATTTTTGATGATATGGCCCTTTTGCTGTAACTGCTTTTGAAATCTTTGTTGTTTTGTTAAATT

CTCCTTGTTCACCAAACTGCAGTGAGAAAGAACAGTGTAAACGTTATCAGCTATTGTGTGCTGTTATTTT

GTTCACACAGCTGTATTTTTATCCATATTCCAAATGAAAATCTGTACAATATTTTGATAACAAAAACTAA

TTTTAATTATAAAATAAAAATAAAAATATTAAAAAAAAAAAAAAAAAAAAAAAAA

**3. Xt-MMP3 cDNA (NM_001030331):**

AAG**ATG**GTTCTCTCCTGGCTCCTCACCCTGAGTGTCCTGCTGCACATTAATATGGTGGCTCTGGTACCGC

TGCCGGAAGAACCCACATACCTGACCCACGGGGACGTACCAGCAGCTCCAGAGCTTTCTGAACTCACCCT

GGAGATTACACAAGTTACAGAACACGACCAGATAAAGGTTCAGAAATATCTAGATCTGTTCTACAGAGGG

GTTGCAGCCATCGGAAGAAAGGCATCTTCAGTTGCAGAAAAGATAAAGGCCATGCAAAAGTTCCTTGGCT

TGGAAGTCACTGGGAAAATTGATTCCAATACCATGACGGTTATACAGAAACCCAGATGTGGGGTCCCAGA

CGTTGAGAGATTTAGCCATTTTGCTGGAAATCCAAAGTGGGGGAAAACAACAGTAACCTACAGAATTCTC

AACTACACGCCGGATATCACAAAATCAGAAGTAGACTATGCCATTGCCCAAGCGTTCAGAGTCTGGAGCG

ACGTTACCCCTTTAAATTTCCAGAAGCTGAATAGTGGGGATGCTGATATCATGATCTCTTTTAACACTAG

AGCTCATGGGGATTTTGATTCATTCGACGGCCCAAATGGAGTCCTGGCCCACGCCTATGCCCCCAGCGAT

GGCATTGGGGGAGATGCCCATTTTGATGAGGATGAACAATGGACATTGGGACCTACAGGTGCTAATCTTT

TTCACGTTGCTGCTCATGAGTTCGGCCATTCGCTTGGGATGTCTCATTCCACTGACACCAACGCTCTGAT

GTATCCAACGGTCTCATTCGGTGTAACCATTGACCCGGCTCAGTATAAGCTTTCTGCGGATGACATCGCA

GGCATCCAGACTTTATATGGAAAAGGAAATCCCAGTCAAGTACCCGTGGGCAAGCCCAATCCAGCGCCGC

CCCCAAAGAACCAGCCGAACAAGTGTGACCCCAACCTAACGTTTGATGCCGTTACAAGCATGAGAGGAGA

TCTGCTCTTTTTCAAAGACGAGGTGTTCTGGAGAAAAAGCGCACGGTTTCCTGAGGTGGAAACTATCCCC

ATTAGTATTATCTGGCCGAGTGTGGGGAGAGTGGATGCGGCTTATGAAGTTGTAGGGAGAGACATAGTAT

ATCTATTCAAAGGTCGGCAACACTGGGCCACAAGAGGATGGACGATTCTTCCAGGATATCCAAAGGACAT

CGCCTCATTTGGGTTTCCAAAGGATGTAAAAAAAATAGATGCCGCAACCTTCATCAGAGAAGAAATGAAA

GCCATTTTCTTTGTCGGAGATAGATATTACAGCTACAGCCACAGAACGTCCGCCATGGATTTCGTGAGAC

CTAAAAAAATTAAATCAGATTTTCCTGGAATTGGAAAGAAAGTTGATGCAGCATTTCAAAACGGATATCT

CTACTTCTCTGATGGAGCCAGACAAGCTGAATTTGATAACAGAGGGAAAAAGGTGGTGCGTTACCTGCAG

AACTACAGATGGATGAGCTGCAAATGAAACGGATCGAGTCTCGCTGTAACATAATGTTTTGTAGGAACTG

ACTCATAACCGTTCCTATTATAGAGCACATATACATGTTTCTGAGGGGCCACAGAATCCCACCCTCTCAA

CTGATTCTGCCAAACCCAACTGGGCGCATTAAATAAAAAGACAAGGTGCCTCCATAGCACTTGGCAGCAG

TGGGATTTGCCCGCAGACATTGTGCCAACGCACATCATAGATACATTTCCATAATAGCACAGTGTTGGGA

TGGTTAATTATCATGTAATTTGGATTTTCCATCATAAATTAAGCCGCAAAATCTTATATAAACATAGCAG

TATCTGGTATGAAACTCAGATGCAATCCTTTTACCATCATAAGACGCGATAGAAATCTATATAACAGGAA

CCAAAATGCCTAAAAGCATTGATGATTCCTTATTATCTGCCTCCTCGGATCTGTGTGGCCCTGGAAGTGA

CAGTCTCTGTATTTGCACAGTTATTAAAGGAGAGCTATGCTAATCGGATGCTGAATATAATCACAATGTA

TGGCTTCTACTGCTGAATAAACTTCTGTGTCTGAAAAAAAAAAAAAAAAAAAA

**4. Xt-MMP7 cDNA (NM_001005043):**

CTAATTTCCCACTGCCACC**ATG**CTTCAAGCAATCCTGTTAGTTATCGTATCACTGTCCTGTATCCAGGCC

ATGCCCGCGCCCCTACCTCAAGATCCTATCAGCCCTTCGGACCGTATGTTTGCAGAGCAATATTTAGACA

CGTTCTATCTGATGAGATCCAAATCAAAGAACACATTTGCTGAAAAAATCAAAGAGATGCAGAAGTTCTT

TGGGATGTCGGTGACAGGGAGGTTGGACTCGGACACCATGGCGATGATGAAGACCCCCCGGTGCGGAATG

CCCGATATTGCAGAGTTCAGACAGTTCCCTGGGAATCCCAAATGGACAAAAACCCGGCTGACATACAGCA

TTGTGAACTACACCCCCGATCTGTCTCGCCAAGTGGTAGACACCGCAATACAGAGGGCGTTTGGGGTGTG

GAGTAACGTCACCCCGCTGCAGTTCACAAAGGTTTCCTCTGGAGATGCTGACATATTGATCCGATTTGGA

GCTCGCACGCATGGAGATTCCAGCCCGTTTGATGGCCCCAGTGGAGTTCTAGCTCATGCCTATGGTCCAG

GGCGCGGCATTGGAGGTGATGCCCATTTTGATGAAGATGAAAGATGGACAAGTTCTAGAACAGGTTTTAA

TCTGTTCCTAGTGGCTGCGCATGAATTTGGCCATTCTCTGGGACTCGATCATTCTACTGACCCACGAGCC

TTGATGTTCCCGACCTACCATTATGTGGACACGCAGGCCTTTCGTTTGTCCCAAGATGACATCAACGGAA

TACAGTCAATATATGGAAGAAGGCAATAGGTGCAAAGTCACATGCTTTAGCTTGGTCATCATTTAATTGC

TACATTGTAAGCTCTTGTATGCAGGACCTTCTAATCCTTCTGTTTTATTTTGGCGCTATATTATTGTAAA

GTGCTGTATAGCTTGCTGGTACCGTGTAAATAAATAAATGATGATTAAAAGCTTAAAAAAAAAAAAAAAA

AAAAAAAAAAAAAA

**5. Xt-MMP9 cDNA (NM_001006842):**

GACCAATAGTCACACATAGTGAAATTTAGGGTTTGTCTTGGTGCAAAA**ATG**GGAGAGTTGGGAGTTTTAG

CCATAGTGGCCATCCTTTGTTCTAGAGGTCACTCTGTGCCTACCAACAGCAAGACCCCTCTGTCAATTAC

ATTTCCAGGGGAAATTCAGAGCAGCATGACTAATGCAGAACTGGCTGAGAGGTACCTTCTACAGTTTGGG

TATATGACCCAAGAGCAAGGCTCAAATGTCACTTTAAAAAATGCGCTCACCCTTATGCAACAAAAGCTGG

GACTTACAAGGACTGGAGTCCTAGATACCGAAACACTGGAGGCCATGAGAAGACCTCGCTGTGGGTTTCC

AGATATTGGGAAATTCAACACATTTGACGGAGATTTAAAGTGGGATCACAATGATATCACATACCGTATC

CTGAGCTACTCCCCTGACCTGGACCCCGAAGTGATTGATGATGCTTTTGCCCGCGCATTTAAAGTCTGGA

GCGATGTCACACCTCTGACCTTCACCCGTATATACAGCGGGGAACCTGATATCAACATTCTGTTTGGACC

TGAGGACCATGGGGATCCTTACCCCTTCGATGGAAAGGATGGCCTCTTGGCCCATGCTTATCCTCCTGGC

CCTGGGGTTCAGGGTGATGCTCATTTTGATGAAGATGAATTTTGGACACTTGGTACCGGAACTGTGGTAA

AGACTCGCTTTGGGAATGCTGAAGGGGCCCTGTGTCACTTCCCCTTCATATTTGATGGGCAGTCATACTC

TTCCTGTACAACCAGTGGAAGATCAGATGGGCTGCCTTGGTGCAGCACTACCCCCAGTTATGATCAGGAT

AAAAAATATGGCTTCTGTCCCAGTGAGTTGCTCTACACCTATGGAGGAAACAGTGAAGGCCAGCCCTGTG

TTTTCCCTTTTATCTTTGATGGTGTGTCATATAATGGTTGTACCAAGGAAGGCCGCCAAGATGGGTATCG

CTGGTGCAGCACAACTGCCGATTATGACCAAGACCAGAAATATGGATTTTGCCCTAACCGAGATACAGCT

GTGATTGGTGGAAACTCCCAGGGAGAACCTTGTGTCTTTCCATTCACATTTCAGGGGAAGAGATTCAATT

CCTGCACTACAGATGGACGGGATGACAGAAAGTTGTGGTGTGCCACTACGTCCAGCTATGACCAAGACCG

CAAGTGGGGCTTCTGCCCTGATCAAGGGTACAGTCTTTTTCTGGTGGCAGCCCATGAGTTTGGCCATGCC

CTTGGTCTGGAGCATAGTGAAGTTCAAGATGCTCTCATGTACCCCATGTACAGCTACGTTAAAGATTTCC

AACTTCATGAAGATGATGTGCGTGGGATCCAATATCTTTATGGTACAGGTTCTCGTCCAAATCCTAATCC

ACCAAAGTCTACTAAGAAGCCATTAGCTACCAAAAGTCCCAGGAGGACTACCACCCGACCTGTTCGTCCA

GTGGACCCTGCACAAGATGCCTGTAAGGTGGAGAAGTTTGATGCCATTGCAGACCTACAGGGAGCTCTTC

ACTTCTTCAAGGATGGGTTGTACTGGACTCTGACTCCCAGAAGTAAGAATGCTCCTCAGAACCCTCAGCG

CATCTCAGATACATGGCCTGGCCTTCCAAGCAGAATTGACACTGCATTCCAGGACCCCACCAGCAAAAGC

ATTTTTTTCTTCTCAGGGCTCAAGTTCTGGCAGTACACAGCTACAAGTGCTTTAGGCCCACGCAGCATTG

AGAAGTTGGGTCTGAACAAAGATGTGGAAAGCATCATGGGATCTTTTGCTCGTGATAATGGAAAGGCTCT

CTTATTCAATGGGGAACGATACTGGAGGCTTAACCTAAAGACATTGACAATTGATAATGGGTACCCACGG

CAAACAGCTGCAGACTATCCCGGTGTTCCTGGTGACTCCCATGATGTTTTTCTCTATCAAGGGAACTACT

ACTTCTGCCAGGATCAATACTTCTGGCGGGTGACATCACGCAAGCAACAAGACATGGTTGGATATGTCAG

CTATGACCTCCTTCATTGTCCACAGAATTAAATGGGGGGGGGGCTGGGTTGAAATGGTACACACTGAAAA

TACTTATATATATTGAAAATCTCTTCCTCTTGGCCTTAAGACTGCCCCCCATTTATTGCTTTGAGGCTTC

CCAAGAGCACACTTCAGGCTGGGATCCCCCCCATACAGTGGCATGGAAATGGAAGCAATGTACAGGTGTT

GTGCTTATAAAGACTGCAACTATTGTTTTACTAACTGTATTTTGTAGGAATAAGGAACAGGTGAATGTCT

GTATGTGATATTAGAGTCTACTGTCTGTATTTTTATACAAAGTACTTTACCCAACAGCAGCTCTTTTCTT

TTTTTCAAACAATTTTAATTATTGTGGTGCATCTAGAAGTGGACAAGAGAGCATGATTATTACACACAGG

GATGGCCGCCATTCTTATGAGAACATCTGGGCTAAATGGCTCCAGTTTCCTATAGCAACCAGTGGGAAAC

TGACTGATACAGAACAAATTAGAAAATAAAAAAGCAAAGACCTGATTATTTTATATGGGCAACTGCAGTG

TGGAATTTAGCATCTACACTTTTCTAGGGTTGAATGGGTGTCTTATGATTTGCCCATCACGTGTATTCTG

AAGTGAGCAATGCAAATATTAATCTATAATATAAATAAACATTCTCTTTGGCCTAAAAAAAAAAAAAAAA

AAAAAAAAAAAAAAAA

**6. Xt-MMP9TH cDNA (Scaffold_29):**

AGAGTAAAAGGCAGAGCATCTGTAGGTATCACT**ATG**GGTTGGCTGGGTATTTTAATACTAGTTGCAATACTTTGTTCGATGGGTCACTCTGCTCCTACAGCCAGTAAGAGCCCCGTGTCAGTTATATTTCCAGGGGAAATCCGTAGCAACATGACTAGCGTGGAGGTTGCAGAGTGGTACCTGGTGAAGTTTGGGTATCTCCCCCTCCAGCAGGGCCCATCCAACCATCATGTTTCCATTAAAAAAGCTCTCAGCCAAATGCAACGCAAGCTGGGACTAAAGGTTACGGGCAACCTGGATGCAGAAACGTTGGAAGCCATGAAAAGCCCTCGCTGCGGAGTACCCGATATTGGCAATTTCAATACATTCGATGGAGAATTAAAGTGGGATCACCATGATATTACATATCGTATCCTGAACTATTCCCCTGACCTGGACCCTGACGTGATCGATGATGCCTTCGCTCGCGCATTTAAAGTCTGGAGTGATGTCACTCCACTGACCTTCACTCGTATATACAATGGGGAACCTGATATCAACATTCTTTTTGGAACTGAGGACCATGGAGATCCTTACCCCTTCGATGGGAAGGATGGCCTCTTGGCCCATGCATATCCACCAGGTCCTGGAATGCAAGGAGATGCTCACTTTGATGATGATGAGTTCTGGACACTTGGTACTGGAACTGTGGTAAAGACTCGATTTGGGAATGCTGGAGGGGCCCAGTGCCACTTCCCATTTGTATTCGACGGTCAGTCTTACAACTCCTGCACAAGTGATGGACGCTCTGATGGGCTGCCTTGGTGTAGTACCACTCCAAATTTTGATCAGGATAAAAAATATGGTTTCTGCCCCAGCGAGTTACTCTACACATATGGAGGCAACAGTGATGGAGAGCCTTGTACCTTCCCTTTCATCTTTGATGGAGTGTCATATGATGGCTGTACCAAAGATGGGCGCCAAGATGGATACCGATGGTGCGGCACCACTGCCAACTATGATCAAGACCACAAATATGGATTTTGTCCTAACAGAGGTATAGCTGTGATTGGTGGAAACTCACAGGGAGATCCTTGTGTCTTCCCATTCACATTCCTGGGGAAGAGATACAACTCCTGCACTAGTGAGGGGCGCAGTGACAGGAAGTTGTGGTGTGCTACAACCTCCAGCTATGACCGAGACAAGAAGTGGGGCTTCTGCCCTGATCAAGGGTACAGTCTTTTTCTGGTGGCAGCCCATGAGTTTGGACATGCCCTTGGTCTGGAGCATAGTGATGTGAAAGATGCTCTCATGTACCCAATGTATAGCTATGTGAAAGATTTCCAGCTCCATGAAGATGATGTGCGTGGGATCCAATATCTATATGGGTCTGGTCCACATCCCGCTCCACCAAAACCCAGTGACAAGCCAATACCAACCACCACCCCTTCCACCAGGACTCCTACTACCACCCCTTCCACCAGGGCTCCTACTACCACCCCACTGACACCATCCGTAAACCCTGCCTTAGATGCTTGTAAAGTGAAAATGTTTGATGCCATTACAGAACTACAAGGAGCTCTGCACTTTTTCAAAGATGGGCTGTACTGGAGTGTGACCTCCAAAAATAAGAATGCTCTACAGTCTCCACGCAATATCTCAGACACATGGCCAGCCCTTCCCACCAAAATTGACACTGCATTCCAGGACCCCACCAGCAAGAACATTTTTTTCTTCTCAGGTCGCAAGTTCTGGCAGTACACAGGAAAGTCTGTTTTAGGCCCTCGCAGCATTGAGAAGTTGGGTCTGAGTAAGGATGTGGAAGGCATCATGGGATCTTTTGCTCGAGATAATGGAAAGGCCCTGTTATTCAATGGGGAGCGATACTGGAGGCTTAATGTAAAGACACTGACTGTTGACAAGGGATACCCACGACTAACAGATGTAGACTATGCTGGTGTTCCAAGCGACTCCCATGATGTTTTTCTGTACCAAGGGAAATATTACTTCTGTCAGGATCGCTTCTTTTGGCGCATGACATCACGCAAGCAAGTGGACAGAGTTGGTTATGTCAAATATGATCTCTTGCACTGCCCGGAGCATTAAATGGAGAGCCAAAGAAGAGTAGGTGGCTGTGTCCATGGCAGTTACCATATTTTTCGGGGAAAAGAGCACATTATTATTCAGTATTTGAGACTGAAGGTTTTATGCTGTACTAGTTGTTGTTGTATCAATGAGCGTCAAGCAAATTTGGTTAACTTGGTTGCCAACTATGCTATAATTCCACTTAATTTTGTACAGATAAGGAACTGC

**7. Xt-MMP 11 cDNA (Scaffold_12):**

A**ATG**CATCTCCTCACCCTTCTACCTGCCCTGTGTGTGCTGGCGGCACACTCAGCCCCTCTGTCACTCATGTACCCCCAGCTCAGAACCCAGGAAAGGCCTCATAAAGATCATGGTTTGTTGCAGACACACTCATTACATTACCCTCATACAAACGGTCTGCTCAATGCTCGCAGTTCAAGGAACCCCCCTCGTTGTGGGGTACCAGATATCCCTGTCCCTCTAGATTCCTCCAGCGGGCGAAATCGTCAGAAACGTTTTGTCCTGTCAGGAGGACGCTGGGACAAGACAAACCTGACGTACAAGATCATCCGTTTCCCTTGGCAGCTAAGTAAGGTGAAAGTGAGACGTACTATTGCAGAAGCCCTAATGGTATGGAGTGAGGTCACCCCTTTGACTTTCACTGAGGTGCATGAAGGACGTTCTGATATCATAATTGACTTCACACGGTACTGGCATGGAGATAACCTCCCATTTGATGGTCCAGGAGGTATCTTGGCACATGCTTTCTTCCCAAAAACCCATCGGGAGGGGGATGTACATTTCGATTACGATGAAGCTTGGACCATTGGAAACAATATAGGTACAGACCTACTTCAAGTAGCTGCTCATGAGTTTGGTCATATGCTTGGCCTGCAGCACTCTTCTGTCTCCAAATCGCTCATGTCACCATTCTACACATTCCGTTACCCACTAAGCCTTAGTGCAGATGACAAGCATGGCATACAGTTTCTGTATGGGGCTCCGCATCCTCCGACCCCTTCCCCAACTCCTAGGGTGGAGGTCAACCAGGTGGAAAATGAGAGTAATGAGATTCCTGCTGCAGAGCCTGATGCATGCTACACTAATTTTGATGCAGTGTCTACAATTCGTGGAGAGTTATTCTTCTTCAAGTCTGGTTATGTGTGGCGCCTTCGTGGTGGGAAACTGCAGAATGGCTACCCAGCCCTGGCATCACGCCACTGGCGAGGCATCCCTGACACTGTTGATGCAGCATTTGAAGATTCTGTGGGAAACATCTGGTTCTTCTATGGCTCACAGTTTTGGGTTTTTGACGGAAAGCTGCAGGCCTCTGGGCCCTTTCCCATTACTGACATTGGTATATCAGTGCCCCAGATTCAGGCCGCCTTTGTGTGGGGCACTGAGAAGAACAAGAAAACCTATCTGTTTAGGGGTGGAGAGTACTGGCGATTCAACCCAGAAACTAGACGAGTGGAAAGCTGGCACTCACGCAACATTGGAGACTGGAGAGGGATACCAAAAGGCATTGACGCTGCCTTCCAGGATGAACAAGGTTATGCCTATTTTGTAAAAGGCCGACAGTACTGGAAGTTTGACCCATTTAAAGTTCGTGTCATGGAGGGGTATCCTCACCTGATCAGTCGGGATTTCTTCAACTGCCATGCAAGCTCTATATCTGTAAACTCCTTAAGATGAGCTGGCTCNNNNNTCTTACCAAGGATTGCAGGGAGTACTCTGTAATACAGACTGGCCAGGCAGGACCACAGGGGCCAT

**8. Xt-MMP 13 cDNA (Scaffold_119):**

AAGCCAAGC**ATG**GCACCTTCACTCTTGTCAGTACTTGTCCTATTTCTAAGCTTTGCTTATTGCCTCTCAGCCCCTGTTCCACAGGATGAAGACTCTGAGATGACACCAGGAGACCTACAGTTGGCTGAGCATTACCTAAACCGGCTTTACAGTTCCTCGTCTAACCTTGCCGGCATGCTGAGGATGAGGAATGTGAAGAGCATAGAGACCAAGCTGAAGGAGATGCAGTCGTTCTTTGGTTTGGAGGTGACTGGCAAACTCAACGAAGATACTCTGGACATCATGAAACAGCCAAGATGTGGTGTCCCTGATATTGGGCAATACAATTTCTTCCCAAGAAAACTGAAATGGCCAAGAAATAACCTGACATACAGGATTGTGAACTACACCCCAGATTTATCCACCAGTGAAGTTGATAGAGCCATCAAAAAAGCACTGAAGGTATGGAGTGATGTCACGCCGCTGAACTTCACTAGGCTCCGCACTGGCACCGCTGACATCATGGTCTCTTTTGGCAAAAAAGNNCATGGAGACTACTATCCATTTGATGGTCCAGACGGCTTGCTAGCTCATGCCTTTCCACCTGGGGAAAAGATTGGGGGTGACACTCACTTTGACGATGATGAGATGTTCTCAACGGACAATAAAGGNTACAATCTCTTTGTTGTTGCTGCTCATGAGTTTGGCCATGCGTTGGGGTTGGATCATTCTAGGGATCCTGGTTCTCTGATGTTTCCAGTTTACACCTACACAGAAACAAGTCAATTCTTACTTCCCGACGATGACGTGCAAGGGATTCAGGCTCTGTATGGCTCTGGTAATAGAGATCCACATCCAAAACACCCTAAAACTCCAGAGAAATGTGATCCTGAGCTAAGCATTGATGCCATCACGGAACTAAGAGGGGAAAAGATGATCTTTAAAGACAGGTTCTTCTGGCGGGTTCACCCCCAGATGACAGATGCAGAACTTGTACTGATCAAGTCCTTTTGGCCTGAACTTCCCAATAAGATTGATGCTGCCTATGAACACGCAGCCAAGGATGTGATCTACATATTTAGAGGTAAAAAGTTTTGGGCTCTCAATGGATATGATATAGTGGAAGATTATCCCAAAAAACTCCATGAGCTTGGCTTTCCAAAGACACTAAAGGCTATCGATGCAGCTGTGTATAATAAAGCCATTGGGAAAACTCTCTTCTTCGCTGGCGACAGTTACTGGAGTTTTAATGAGGAGACCAGGACCATGGATAAGGGCTTCCCAAGACTCATCTCAGAGGACTTCCCAGGAATCGGCGAGAAAGTAGACGCTGCTTATCAGAGAAATGGTTACATCTATTTCTTCAGCGGAGCACTGCAGTTTGAATACAGCACCTGGAGCCAGAGAGTAACACGCATCTTGAAAACCAATTTTGTCCTGATGTGCTGATCTTGAAATGAATAATTNTATACATATATATATATCTACAGATATCAGGCTGCTGAGCGGCCTCCTNTTTTATGCAGTTGACTACCGCCTTGGTCTTCAGCAAAGCTATGGGTTGGATATTAAGGGTAAATATAGCGCAGGTTAGCTACAGAGAACAGTAACACTTTCTTTCATACGTTTCCAGATTCCATACAAATGTTAATATTTATATATAACACTACAAGTACCTGCAGGTATTTATACCTACAATGCAAT

**9. Xt-MMP14 cDNA (NM_001030388):**

AAGAGGGAGAGAAGGATAGTGAGGGGAACAGCGTAGTGGGGGCAGTAAGAGCAAGGATACAAGGAAAAAA

AAAACTTGATATAACATGCACTGTGAATAACCTTTATAAATGACTCACTTTAAAAAGAGTTAACACATAG

TTTGAGCTGTGAGGGGGGTTAAGGGGACTGAACAGAGATTAGTGGATAAGAAACCTATTCTTGCTCTACA

GACAACCACCCTTCAGAGGAGTTTTTACTCGCACAGG**ATG**GAGCCTGTGAGAGCAGCCTGGATTTGCCTT

TTTCTGTGCTCTGTATGCAGCTCTAGCTCCGCCAAATTCAGCCCAGAGGCCTGGCTTCAGCAGTATGGAT

ATCTGCCCCCTGGAGATTTGCGGACACACACCTTGCGCTCCCCACAGTCCATGAATGCTGCAATATCTGC

CATGCAAAAATTCTATGGATTAAAAGTGACAGGATCATTCGACAGTGAAACCGAGAGAGCAATGAAGAGG

CCTCGATGTGGAGTCCCTGACAAATTCGGTGCTGAAATAAAGGCAAATGTGAGACGGAAGAGATATGCCA

TCCAGGGTTTAAAGTGGCAGCACAAGGACATCACATTTTGCATACAGAATTACACTCCCAAAATTGGCGA

GTATTCTACTTATGAGGCAATTCGGAGAGCCTTTAAAGTTTGGGAGAGCGTGACACCACTGCGTTTCCGG

GAGGTTCGATATGTGGATATCAAAGATGGATACACTAAACATGCTGATATCATGCTTTTTTTTGCTGAGG

GTTTCCATGGAGACAGCACTCCATTTGATGGAGAGGGTGGCTTTTTGGCACATGCATATTTCCCTGGGCC

AGGCATTGGAGGCGATACACACTTTGACTCTGCAGAACCTTGGACAGCCAGGAATGATGATCTGGATGGT

AATGATCTGTTTCTGGTGGCTGTACATGAACTTGGTCATGCTCTCGGTTTGGAACATTCAAATGATCCTT

CAGCAATTATGGCTCCATTTTATCAGTGGATGGACACACAGAATTTTCAGCTACCAGATGATGACCGCAG

AGGAATCCAGCAGCTCTATGGACCCAATCACGGAGAAGGTCTTCCAACTCGTGCACCTCGTCCCACACGA

GCTCCACGACCCACACAAACCCAGAGACCAGATGATACCCCACACGATCCCAACCCACCCACTTATGGAC

CTGACATCTGTCAGGGCAACTTTGACACAATTGCTATGTTAAGGGGAGAGATGTTTGTTTTCAAGGAACG

CTGGTTCTGGCGTGTTCGTCATAAACGTGTAATGGATGGATACCCCATGCCCATTGGGCAATTCTGGCGA

GGTCTCCCTAGCTCCATTAATTCAGCTTATGAACGAAAGGATGGCAAATTTGTATTTTTCAAAGGGGATA

AGCACTGGGTGTTTGATGAAGCTATTTTAGAGCCAGGCTATCCCAAAACCCTGAAGGAGCTGGGCCGAGG

ACTTCCTAGCGACAGGATTGATGCTGCCCTCTATTGGATGCCAAATGGAAAGACCTACTTCTTCAGGGGG

ACCAAGTACTACCGGTTTAATGAGGAGATGAGAGCAGTGGATCCAGATTACCCCAAGCCTGTCAATGTGT

GGGAAGGCATCCCAGACTCTATCAAAGGAGCATTCATGGGCAGTGATGGAGCCTTCACTTACTTTTACAA

AGGTAACAAGTATTGGAAGTTTAACAACCAGCTGCTAAAGACAGAGTCTGGCTACCCTAAATCTGTTTTG

GTCGACTGGATGGGCTGCAGTACTGCCCGACAGCCTGATGATGATGTTGACAGGGAGGTGGTTATTATTG

AAGTGGACGAGGCAAGTGGGGGAGTGAGTGCTGCAGCTATTGTAGTTCCTGTCCTTCTCCTGCTTTGTGT

TCTTGCCCTTGGACTGGCTGTCGTGCTCTTCAGGCAATGTGGAACACCGAAGAGGCTCCTATATTGGCAG

CGGTCCCTTCTGGATAAAGTGTGAACACCTACACCAAGAAATTTGCACCCTCCCCCTGAATTACTGCCTC

TCCAGCCCTCCTCTGTCTGCCTCACACAAATAATACAGGGAGCAGTATCAGCTTCCAGGAAAGAGAGGAG

TTCTAATGCAATTTGCACCATTTTTGCCTTACTATTTTTTTGTGGCACGTGTCGTGTTTAACCCCTTCAC

TGCCAGACCGGGGCCACGATGTTTGAACGAGTACAGATTATGATATATGATTGGTCACTGGGGCCTGTTG

GCTCAAACAACAGCAAGTTACAAAGGTCATGACAGAGAGGCACATGAACTTGAGATTCAAAAGATCTAAA

ACTGTTAAGTTTTTGAAATTCAAAATAAATATGGGCCTTCTTTCTAGTCCTTCCCACAACCACCCAACAT

TTTAACACCAGCCAAGAGTAAAGAACCCTGTTTCTAGCTGGGTATGAATGTGCTGGTCAGAAGAATTTTT

TTTTGTCCAGGAATATGGAGGTGTACACTCAGGAACATACATTTTGAACAGCAGGGAATTATATGACACA

GTATTATGAAAATCTAGAAGGAGGTCCGTGCCATGCTGGTGAGATACTAAAGGCAGGATATGAATAGCAT

AACTACTGGAGAGCACACTAAACTGGGTATTACGCACAAGTCTTGTTTACGAGGCTTCTATATAGGCCAA

AGAGATCTGTCAGTTTTATGGAAAAGTTGTACTTCATGTAGAAGTGTCTTTAGGGGAAGATATGCAAAAC

TTGCTAAAATATAAAAGTATTCTCCACAGTACATCTGCAGTGTAGCATATACATTAATAGCACTGGTGCC

TAAAAACAAAAGACAAAGCAGTTTATTTTAATTGTCCAAGCTTGCAACACAAGGGATTTGTTTCTTTACA

TTATGAGCTTCTCAGTCTTTTCTCCCTCTGAGTATTGCTGTAATTCAGTGGTGGCAGTACTTTGGTGGAT

AGTATGTTGAATTGTGATCCCTTCATATTCCTGGACATTCTGTCTTTACTATATGTGTACCCTAAAAAAA

ATACCGCCCATCCCCATTTAAGTAATAATTTGGTGGTAACTAAAATCATAAGAAAATTTAGTAATGGAAT

TGTTCTGTAAGGTGTGACATGGGGATTGATGAGGAGTTAGATTTTTTTTTTTTCTTTTTTTTTTTGCGGA

GCAGGAATCTGTGTATAGAAGCAAGGGGGACAAGTCCAAGATAAGCATTGCCCAATTCCTATAGTGCCTT

GTTAGGTGTCCCTCAGCCACCCATTTTCTTCTATGACGCCTCCTCTATGCCCTCCACCTATTTAGTGATG

ACTCTGGAGGAGAAAGGGTTAATGTGCATCTCCCTGGTACCTACAGATCATTGCTGGGGGTTGGACACTG

CTCTGTAGATACTTTGGAGGGAATTAGTTATTTTCCGTTTTATACAATCTGGCAGCAAAGGGGTTATTCA

CTGCTCGTTGGGTCTAGCAGGAAGGCATTACATCATGCTTTGCCAGGGAAAGTGCCCATTCCAGTGGTAA

GTCTAAGAAAAGATTCACCATTTCTGTTATTCACTGCTATGGGTGTCTGTGGAAGGAAAGGGGTGAAGAA

GGGCAAATCACATGGTACGGTTAAGGGGGTGGGATCACATTCGCTTCTTTCCCTTCTCTTATTCTTTGGT

TTATATTTTTATTATTATAATTAATATTATTTGGTAATGAGGCTTCGTGCATGCACACAGCCCCTGCAGG

TCAGGTGGCTTTGTGTTTTACACCTGTCTACTGCAAGAGGGGTCTATGGAGCAACTCAGGTCTTGGTATA

AAGAGCAGCGCTCTGCTTGGCCCTTCTGCAGTGAATGGGTGACTTCTTTTCCCCTACCCTTAACAGCCAC

ACTGAGGCCAGTGCTTGCACATTGATTGGGGGAGTTGCAGTTTGCCCCTGCTAACCTCCAACCCCAACCC

CCAAGTGCATTATAACATCTATAAGAACACGGATACAAACATAGACTCTAAAAGGGCAAACTAACTACAG

CATTCCCTCGAATTAATGTATTTGGGTATCCAGGGGCCCCGTTGTTGGGTTAATTAAAGCTTGTCAAATG

GATGAATTTCAGTTTTTTTAATTGTTTTTATGTGTCCTTTTTTTATCTAAAGAAAGCTATTTGTAATGTC

CAAAAACATTTGTAACATTAGATCTATAAAATGCCAAAATGTGAAAAAAAAAAAAAAAAAAAAAAAAAAA

AAAAAAAAAAAAAAAAAAAAAAAAAAA

**10. Xt-MMP15 cDNA (NM_001015921):**

TTCGGCGCTTTGCTCTCTGCTATAAGCCATGGCCACAGTGACTATACGAGCTGAGTGTGCGGCCTCCCTA

ATGGCTACTGAGAGTGGACTGATCCATGTGCAAGGCGACTGAGGGCTTGGAGCGCTGAGCAGTGCCTGGC

TGAGAGCTACCCTGAGAGCAGCTCTGGTTGTGCTCAGTCACTTCTGGCTTAGGGAGTGTCACCCAAGTAG

CAGTTTCAACACGGGGCGAAGAAAAACAAAAGGGGAGAAATTGAGCCCCCAAAAAT**ATG**GCAGATGGTTT

GTTTTGGGGGAGACCTCGCTGGACAGAGCGGGGAGGGGCTGCAAGACTGCTGCTGGGATGGACCTTTCTA

TTAATCTGCTGTCAGGGATACGCCGCAACTAGTGATTGGGATTCAAAAGCCGAGGCCTGGCTGAAGCTCT

ATGGATATCTTCCTCAGACCAGTCGTCAAATGTCCACCATGCGGTCGTTACAGATCCTCTCCTCAGCTAT

CTCAGAAATGCAACGATTCTATGGAATCCCAGAGACTGGAGAACTGGACCATACAACTACAGAATGGATG

CAGAAACCTCGTTGTGGGGTGCCTGATCAGTTTGGGACCCGAGTCAAATCAAATATGAGAAGAAAACGCT

ATGCTCACACAGGACGCAAATGGAACCAACAACATCTCACATATAGCATCCAGAATTACTCAGATAAACT

GGGAATGCACAACTCTATTGATGCCATACGAAAAGCCTTTGACGTGTGGTCAAAGGCAACCTCGCTGACT

TTCAGAGAAGTGCCCTATGAAGCAGTGCGCCAGCGTCACTCCAGTGCAGACATCCTGATCCTTTTTGCCT

CTGGTTTCCATGGTGACAGTTCCCCATTTGATGGCCCTGGTGGCTTTCTGGCACATGCTTATTTTCCTGG

CCCTGGCATGGGAGGAGATGCTCATTTTGACTCAGAGGAGCCATGGACTGTAGAAAACATGGATCTGGCA

GGGAATCATCTGTTTCTAGTAGCTGTGCATGAGCTGGGGCACTCTCTGGGGCTAGAGCACTCAAATAACC

CTTCAGCCATCATGGCTCCATTCTACCAGTGGATGGATACTGAAGATTTCCAGTTGCCTGAGGATGACAG

ACGAGGAATACAACAGTTGTATGGACCTCCAGTGGATCAAATTCCCAGTACCCAGTCTCCCTTACCAACC

CCAGGCAAACCTGAAGTGCCTGACAAAAGACCCCCTAAACCACCTCCTAGAGGTAAACCAGAACGGCCTG

GTAATGCACCTCCTCCTCGCAGTCCCTCCAATCCTGACCAATACGGACCCAACATTTGTGAGGGCAACTT

TGATGCTGTCAGTGTTCTTCGAGGAGAGATGTTTGTGTTTAAGGGTGCCTGGTTTTGGAGGGTACGACAC

AACCGAGTCCTGGATAACTACCCCATGCCAATTGGTCACTTCTGGCGTGGGCTTCCACCTAACATAACTG

CTGCCTATGAGCGACATGATGGAAAATTTGTCTTCTTTAAAGGAGAGAAGTACTGGCTGTTCCGTGAGGC

CAATCTGGAAGCTGGTTACCCACAGCCGCTGACCAGCTTCGGATACGGGATCCCTTATGACCGAATTGAT

ACAGCCATATTTTGGGAACCTACAGGACACACATACCTCTTCCGAGGAGACAAATACTGGCGATTTAATG

AGGAATCCCGTTCTGCAGATGTTGGTTACCCAAAACCCATTACAGTCTGGGCTGGTATCCCTGACACCCC

CAAAGGGGCCTTCTTGAGCTCTGACTCAACTTACACCTACTTCTACAAAGGTGCCAAATACTGGAAGTTT

GATAACCAGCAGCTGAAGACAGAGCCGGGATACCCTAAATCCATTCTGCGAGACTTTATGGGCTGCCAGG

AAGAAGTTTTTCAGGACCCGGATGTTGTTCCACGATGGCCAGATGTTGAACACCCCCCATTTAATCCTGA

TGTTGAGTCAGAGGATAAAGATGACAAAGACAGTGACAGTGCTGGACGGGAGGATCCAAACACTGGACGT

GATGTGGATGTTGTGGTCCATATTGATGAGTACACACGGGCTGTTAGTGTTGCCATGGTAATTGTGCCAC

TACTACTACTACTGTGCATTCTGGGACTTATTTATGTCATTGTGCAGATGCACCGGAAAGGACCCCCTAA

AGCCTTACGATACTGTAAACGTTCTTTACAGGAATGGGTCTGATTTTCTTTTTTTTTTTTTTTTATTGCT

GCCATTTTTACCTCTCACCATCCACCTATTTCTTTTGTTCCCACTTCATTTTTATATTCACAACTCATTT

TGTGTGGTGCTAACATGGGGGAGCTGGGATATTTAATGTTAATATTTGTTCAGACCCCTCCTAAGTTTTC

ATTCCACTGTTTCTTGACTATGGGGGCAAAAGAAGAAATACTGCATTTTCTGACACTTTAAAGGGTGCTT

CATATTTCCTGAGGTTAGTTTCCGATTCAGTGGTCCACCTATGTGGTGCAGAGGTGATAGCAAATTACAT

CTGGGATTTCATGGTGTATGCGCAGTGTCTATATTCCTGATGATGTAACACATAAACCTCTGCATTGTTT

GGTTAAGCCTTCCAAGGCTGCATTTCTGTGGGCCATCCTAATGTCAATGCTCACTGGCAGGAAATGGATG

ACCCTGTTAATTGTAATTATTCTGTAGCCCTAGCACTGTATGGATACAGTTGAATGTTTCTCTAGCATTT

AGAATGTTGAATATTTATGCCATGCATTTTGCACTGGAGATGTCCTAGGGTGGGAGCAGATCAGCAAAGT

GGGGAATAAACAGGCCTGAATGGGTACAAAATAAAAATAGTAGGGATGCACCAAATCCATGATTTGGCTT

GGGATTCGGCCAAGATTCATCCCTTTTTAGCAGGATTTGGGTAAATCCATGCTTCTGGCCGAACTGAATC

CTTAAACTCATTTGATGTCACATAAAAACGAAAGGTGAAAATTTGCGCTTCGCACATAGATCCTTAAATG

CAAAATAAGATTTTGGTTCAGTATTCGGCCAGATCTTTCATGAAGGATTCAGGGTTCAGCTGGATCCTAA

AATTGTGTATTCGATGCATCCCTGAAATATAGTGATTCTACCTGCACTCTGGCTGCTCAAATAATATTAA

AGGTACCTCAGAACTGGGGAATCTCTAAGCCTGGCATGGAAGGGTCGTGTTGCCTTAATCGCCTATGATG

ATCCCTGTGCCATTCACATGAGAGTCTGGACGGGGATATTTTCCTTTGGGTATTGGAGGAAGAGCGTTGT

TCCTGTGGAATGCCGGTATTAGTAAGTTAATAAGGAGTTGCTTTCTTGCACTCTCTGATATTTCTCATAT

CGGGCTGTGCCCATGAAGTCTATGAACAACGATCTTAGTTTCTTCAGTGTCTGTACGGTAGATGTTCACC

CAGATATTTTAAAAATGTGTTTGTTGTTTATTAAAGATTTTTGTAATGGTAAAAAAAAAAAAAAAAAAA

**11. Xt-MMP16 cDNA (NM_001015992):**

CCCCGGGGGGAGGCAGACTGGGCACACAGGCCTCTGTTAGGGCAGGGAACAAGGCACACAGACCCGGGGA

ACTGGTTCCACTTGGCCGCTGGTTATGAATGAGATCTTCCCCCTGTGTGTGCCACCGGAGCCCTCCATCG

CTCGCCTATCGCAGCGCAGCCTCCCCTATGCTTGGATAATCCCGCACAGCTGCTCTCCTCCAGCACCGGG

GACTTTGTGAAGGAAGCGGCCAGCCTCCCGGAGCAGATAGCGCAGCTCACC**ATG**GTCTGGCTCCCCCCAG

GCACTAGGAGCCCGTTACAGTTGTGCCGTTCCGTGGGGCTTATTGTGCTCTCCTTGCTCTGGATTGTGTG

TGCGGTGGGCGCAGGGGAGCAGGGTTTCAGCGCAGAGATGTGGTTACAAAAATATGGCTACCTTCCACCA

ACTGACCCCAGAATGTCGGTACTGCGATCTGCAGAGACCATGCAATCCGCTATAGCTGCCATGCAGCAAT

TCTATGGGATCAATGCTACTGGGAAGATTGACAAAAACACAATTGATTGGATGAAAAAACCTCGGTGTGG

AGTGCGTGATCAAGCAGGACCTATTTCCAGATTTAATGTCCGACGGAAACGATATGCCTTAACGGGACAG

AAGTGGCATCACAAACATATCACCTACAGTATAAAGAACGTCACTCCAAAAGTAGGAGATTCGGAAACCC

GTAAAGCCATTCGCCGTGCCTTTGATGTCTGGCAGAATGTAACTCCGCTGACATTTGAAGAAGTTCCATA

CTGTGAATTAGAAAATGGCAAACGGGACGTAGACATCACTATTATTTTTGCATCAGGTTTTCACGGGGAC

AGTTCTCCATTTGATGGAGAGGGGGGATTTCTGGCACATGCTTACTTCCCAGGACCTGGAATTGGGGGAG

ACACACATTTTGACTCGGATGAACCATGGACCTTAGGAAATCCTAATCACGATGGAAATGACCTGTTTCT

AGTTGCAGTCCATGAATTGGGTCACGCATTAGGCCTAGAACATTCCAATGACCCCACGGCGATTATGGCT

CCATTTTATCAGTACATGGAAACAGACAACTTCAAGCTACCTACTGATGACTTACAAGGAATTCAGAAAA

TTTACGGTCCACCAGAAAAGGCCCCAGCACCAACAAAACCCCTTCCTACTGTGCCACCACACCGCTCTGT

TCCTCCAGTAGACCCTCGCAAGAATGATAGACAACCTAAACCACCCAGGCCTCCTACTGGAGACAAGCCA

TCTTACCCCGGAGCCAAGCCCAACATCTGCGATGGGAATTTTAATACACTCGCAATCCTACGCCGAGAGA

TGTTTGTTTTTAAGGATCAATGGTTCTGGCGGGTAAGAAACAACAAGGTTATGGATGGCTATCCTATGCA

GATCACCTATTTCTGGAGGGGACTGCCTCCTGGCATTGATGCAGTGTATGAGAACCGCGAGGGGAACTTT

GTCTTTTTTAAAGGTAATAAATATTGGATTTTTAAAGATACAACCCTTCAGCCTGGGTACCCTTATGATT

TGATGCACTTGGGCCACGGCATTCCGCCTCATGGCATTGATACTGCTGTTTGGTGGGAAGATGTTGGGAA

GACATACTTCTTCAAAGGTGACCGGTATTGGCGATACAATGAGGAAATGAGAGTAATGGATCCTGGCTAT

CCAAAGCTAATCACAGTATGGAAAGGCATTCCAGAGTCACCACAAGGAGCCTTTGTTGACAAAGAAAATG

GCTTTACATATTTTTATAAAGGAAAGGAATACTGGAAATTCCAGAATCTGAATCTCCGGGTAGAACCTGG

TTACCCGAGATCGATCCTTAAGGATTTCATGGGATGTGATGGCTCCACTGATGGAGACAAAGAAAGAACC

AGTCCCCAAGATGATGTAGACATTGTCATCAAGCTGGACAACACAGCCAGCACTGTGAAAGCCATAGCCA

TTGTAATCCCATGTATATTGGCCTTGTGCCTTCTTGTATTGGTTTACACTGTGTTTCAGATCAAAAGGAA

AGGAACACCCCGCCACATACTTTACTGTAAACGGTCTATGCAAGAGTGGGTGTGATGTAGGGTTTCTTTC

ATTGAAGTTACTTGAGGTTCCACATGAGAGCTATTATGCTGTTCCCTAGCTAGAAGCAGGCATCTGTGAT

CCAGGCTCATGGTCGATCTTAAAAACCACAAGCGGTTTGGTGTCCTGCACTCGAGTGGGGATTCAATCAT

CTGGGAAGCTTCCATGAAATACAGTTTCTGCTGTTCCTCCAGTCCTTTGTATTTCTTTGTCATTCACTTT

TAGGCCTTTCCTCTGCACGTCGAATGCTCAGTTTACTCTCAGTAGTAAACGCAAAGAGGAGAATAAATTT

AACAGCGATAATAAAGTTTGATTTTCGTTCGGGAAAAAAAAAAAAAAAAAA

**12. Xt-MMP17 cDNA (NM_001102999):**

TGTCTTTGAACTTCCAGCTGGCTGCCACTGCTGGCTGCGCAGGTCACATCTTAACCCCAGATCCCTTGAA

GCATCGGGCTGCTTTCCCCTGTGCCTGTTACCGTGCTTCCCATGTGATCTGAGGAGCTGCCCTTGGTGCT

GACAGTTCGATGGTACCTTGACCTGTTGTGCTGAATTCTTCAAACTTCCTTGGATGTTGGCAGAGGTTAT

GGGATGTGATGTGATGGCATGCAGATATTCTTGCAGCAGGGTCCGGCATGTGGAATTGTGCTGATGCTGC

AGAGGAGATGTGAAGAAGAGGGCTTGGGTGTACACATGAATGTCGCCTTCATCTACCCAGGGCTGAGGAG

GAAGACAAGGG**ATG**CTGTTGATCCCCTCCAAAGAGAGTTTTTGGCACCAGGAGATGTTTCTGATCGGCCT

TTTGATTGCTTTACGGGAATCCATGGCAGCTCCAACCCCTACAGCTGAGGATATAAATCGGGGAGTTGAT

TGGTTGACTAAATTTGGCTATCTGCCCCCACCCGATCCTGTTACAGGGCAGCTGCAGACACAGGAAGAAC

TGTCTAAAGCCATCAAGGCAATGCAAGAGTTTGGTGGCCTGAAAGCTACTGGGATTTTAGATGAACCCAC

TTTGGAGCTGATGAAAACCCCTCGTTGTTCTCTGCCTGATCTGTCCCATTCACAAGCTTCAAGAAAGAAA

CGAAGTGCCCAGCCCGTGACAAAGTGGAGTAAAAGGAATTTGTCCTGGCGGGTTCGAAATTTTCCTAAAG

AGTCTTCACTGGGACACGACACAGTGCGGGCACTAATGTATTATGCCCTTAAAGTATGGAGTGATATTAC

CCCACTGAACTTTCATGAGGTAGCTGGGAATAATGCAGACATTCAAATCGATTTCTCAAGGGCTGACCAC

AATGATGGATACCCATTTGATGGCCCCGGAGGGACAGTAGCTCATGCCTTCTTTCCAGGAGAACATCTTA

CATCTGGAGACACTCATTTTGATGATGAAGAATCTTGGACGTTCCGATCTTCAGATATTCATGGCATGGA

TCTATTTGCTGTGGCAGTGCATGAATTTGGCCATGCCATTGGACTGACTCACATTTCAGACATGGAATCC

ATCATGAGACCTTATTATCAAGGACCTGTGGGTGATCCCTTAAAGTATGATCTGCCATATGACGACAAAG

TTAGAATCTGGCAGCTATATGGAGTCCGAGAGTCTGTGTCCCCCACAGCAAAACCAGATGTTTCAAAAAC

AGAGGATCATCCTTTTCTGCCTGATCTGCCAGAAAACCGCTCTACCATTCAGCCAAGGAAGGATGAGCCT

GACCGTTGTAGCACACATTTTGATGCAGTTGCTCAGATCAGAGGAGAAGCATTCTTCTTTAAGGGCAAGT

ATTTCTGGAGGCTCACACGGACAAAGCATTTGGTATCCCTCCAGCCGGCTCAGATCCATCGCTTCTGGAG

AGGTCTCCCACTGAACATGGATAGTGTGGATGCTGTGTTCGAAAAAACTACTGACCATAAGATAGTTTTC

TTTAAAGGGGACCGGTACTGGGTTTTTAAAGATAATAATGTGGAGGAAGGATACCCAAGACCAATCACAG

ACTTTGGACTGCCACTAGGAGGTATAGATGCTGCCTTCTCCTGGTCTCACAATGACAAGACATACTTTTT

TAAAGACAATCTTTTCTGGTCATATGATAACAAAGAACATAGGATGGATGAAGGATATCCTTTAGAGACC

ATGCTGTGGAAGGGCATTCCGGCTACTTTGGATGATGCCATGGGATGGTCTGATGGAGCTACATATTTCT

TCAGAGGTAAAGAGTACTGGAAGACTGTGAACAGCAATATGGAGGCAGAACAAGGCTATCCACAATCCAT

TGCAAAGGACTGGCTTGTTTGCAGTGACATGCTTTCAGATGGACCTAGTTCTGAAAGTACCCAGACTGGT

GCTCATTCTGGGCAAGGAAAGCAACATGAAAGCAGGTCCGAAAATGGTTATGAGGTCTGTTCCTGTACTT

CCTCAGGGGGATCTCTTTTTGGAAGTGCATATGATGACCTTTTGTGGTTCTTGTCTATAATCCACTTGTT

CACAGCAGCCTTGATCTCAATCCTATGATGCCCTAACCTAATGGACTCCACCACACAAAAGACTTGAGGA

TCAATAAATGGGATCCCATCAACGCCGCACTTATGTCGGCCAAACATTCCGAGGAGCTTGGAAGTGAAAG

ATACCAAATTCTCCATGAGAAATGATCTTTAATTTTTAAGATAATATTCTGTAAAACAGTGCTCAAATTA

TTGGAACATTGGCAAGGCAATATCACTGCGGAATAGATGAGCAAAATTCTATGGTAGCCATGTGCCTCAT

AACTGTCTTAAATGGGTCTCTGCGATAAGTACTTATCAAACAGATCTGAAAGACGTGAAACATCAGAAGC

GTTCTCTCTTTACCAAAGGTTTTGCTAATTAATTCCAAAGTGTCAAAGTTTAAAAAGTAATAACATGTAA

TAAGCTCTTTATTCTAGTCTGTCATTTGAAACAAGTACATTTTTAAATCTCAAAACTTTAAGGAAAGCGG

CTTAGCCTTCCCAGTGCTTATACAAGATAGAGCGAGAGAGGATGACCCAGTAATCTGAGCACTGCCAAGT

GGATTCTGTAATAGTGCTTCTTTGGATATCTGAAAACTGGATGTAAAATAAAGAGGCACATCTTGTTTAC

ACCAAAAAAAAAAAAAAAAAAAAAAAAAAAA

**13. Xt-MMP18 cDNA (NM_001030330):**

CGGCAAATCCTAGAAAGCCTTAGAAGG**ATG**AAGAGCCTCCTGCTGAAGCTGCTACTGTGTGTAGCCCTCG

CCGCCGCCTTCCCTACAGATAAACAAGATGCACCCACAGGAACCAGTGAGGAGATGGCTGAGAACTACTT

GAAGAAATTCTACAGTCTCGGCACCGAGGGGGGACCAGTTGGGAGAAAGAAAAACAACCGTCCTTTCACT

GAAAAGCTCCAGCAGATGCAGAAGTTTTTTGGCTTAAAGGTGACGGGGATACTGGACAGTAAGACAATAG

AAGTCATGCAGAAACCCAGGTGTGGAGTCTATGATGTTGGGCAGTACAGCACCGTCCCAAAAAGTTCTGC

ATGGCAGAAAACTGATCTGACCTACAGAATCATAAACTTCACTCCTGACCTGCCCCAGGCCGACGTGGAG

GCTGCCATTCAAAGAGCTTTTAAAGTCTGGAGTGATGTGACACCTTTGACCTTCACCAGAATCTACAATG

AAGTATCAGATATAGAAATCTCATTTTCAGCTGGAGATCACAAAGACAATTCTCCTTTTGATGGACCTGG

TGGCATTTTAGCCCACGCCTTTCAGCCCGGCAACGGCATTGGTGGAGATGCCCATTTTGACGAAGATGAA

TCCTGGACCAAGACCAGCCAATTGTACAATCTGTTTCTTGTTGCTGCCCATGAATTCGGACACTCCTTGG

GGCTTTCTCATTCCACTGATCCAGGTGCCTTGATGTATCCATCTTACTCAAGTACCGACCCCAATGCATT

TCAGCTTCCTCAGGATGATATAAATGCTATACAGTATCTATATGGAAAGTCTTCCAATCCAGTCCAACCA

ACCGGCCCAACCACTCCTACCATATGTGATCCCAACGTGGTTTTCGATGCTGTCACCACCTTGAGAGGAG

AACTGATTTTCTTTCTTAACAGGTTTATATGGAGAAAGCATCCCCAAGCATCCGAGGCTGAACTCATGTT

CGTGCAAACATTCTGGCCATCACTGCCCAACGATATCGATGCTGCCTATGAAAATCCTATAACGGAGCAG

ATCCTTGTGTTTAAAGGAGCAAAATATACAGCTCTCAACGGCTTCGATGTATTGCCAGGCTACCCCAGGA

ACATCTACAGCCTGGGATTCCCAAAGACCGTGAAGAGAATCGACGCAGCTGTTCATATTGAGCACCTCGC

GAAAACATATTTCTTTGTGGCTAATAAATATTGGAGTTATGATGAAGATAAACAGCAAATGGACAAAGGC

TTTCCAAAACTAATAAGAGACGATTTCCCAGGAATCCCTGAGAAAATTAATGCAGCCCTTTATTATAGAG

GTCGCCTGTATTTCTTCATTGGACGGGTCCAGTTTGAATACAACCTCAACACTAAGAAAATTGTACAGGT

TTTGAGAAGCAACAGCTGGTTGGGCTGCTAACACCACAGACTCCCTGCTCTCTCACCACAGACTCCCTGC

TCTCTAATAGAGGACAATATTGACAGCGACTGTTGCTCATTTCTGCAGCTGTCAGTAGTTTCCATATCAT

TTCAATTCAATGCAACTTTTGGGTTTATTTAATCATTATTAATTAATATTTTATACTACTTTTTCTTTAA

TAAATATTTTTTGTTT

**14. Xt-MMP19 cDNA (BC153750):**

CAGCTGGGATATCCTACATACAATGAGCTACAGGATGCTGCTGTTTCTACTGCTTGGGACTTTGCCATTC

TTTGTCTATGCAATGCACATTGAACCCAGCTCTGGGGAGGAGGCCAAGAGATATCTACAGCAGTTTGGGT

ACCTACAGAAGCCCCTGGAGAGCGACACTGAGGACTTCAGCTCAGAGGAAGTACAGGAAGCGCTACGTAT

TTTCCAGCTGTCTACACACTTACCTGGAACTGGCGTCTTGGATGAGGACACCATAGATAAGATGAGGCAG

CCTCGATGTGGAGTAGAAGATCCATTCAACCAGAAGACTCTGAGATATCTGCTCTTAGGTCGCTGGAGGA

AGAAGAACCTCACTTACAGGATATATAATTACACGCCAGATATGAGCCTGGCTGCGGCCAGGTCTGCCAT

CCTAGCTGGCTTTAAGTATTGGAGCGACGTCACACCCTTGACTTTCAAGGAGGTTACCCGAGGAAGGGCA

GATATACGCATTTCTTTTCACCGCCGGGGTAATGGCTGTTCACGCCCCTTTGATGGTCCTGGTAAGGTGC

TGGCCCACGCCGATATCCCTGAATTGGGCACTGTGCATTTTGATGAGGATGAGTTGTGGACAGAAGGGAC

CTATGAAGGGGTGAACTTGCGCATCATTGCAGCACATGAGCTGGGACATGCACTGGGTCTGGGACACTCA

CGTTTCAGAAGTGCTCTGATGGCTCCCATATATTTTGGCTACAAACCAAACTTCCGACTCCATGATGATG

ATGTGAAGGGGATTCAGGCCTTGTATGGTAAGAAAGATAAAATAGAAGAAGAAGAAGTTGTAACCACTGA

GGAGCCTGCCATCTCTGCAGAGCCAACACCCACCAGCCTAACCCCTGATCCTTGTAATGACAACCTTGAT

GCCATCATCCTAGGTCCCTATGGAAAGACTTATGCCTTTAAAGGGGATTATGTTTGGACAATCACAGATT

TCGGAATTAGCCCCCTTATTCGCATCCAGTCTCTGTGGAAAGGCCTGCCCGGAAACATAGATGCAGCCGT

GCACTCTCCGCGCACACAGAGAACCTATTTCTTTAAAGGAGACAAACTGTGGGTGTACACAAATTTCAAG

CTGAATCCTGGATACCCCAAGTTGATAACAAGGGTTCCACCAAATATCAATGCTGCTTTGTACTGGGAAG

TAAACAAAAAGATCTTCTTGTTCAAGGGGGATGGGTACTGGCAATGGGACGAGTTGGGATGGAGCAACCT

TTCCTCAAAGAAGATCTCCAGCCTGTTCACTGGCATTCCCTCTCAGCTGGATGCCGCTGTGACGTGGAAG

AACGGCAAGATTTATTTCTTCAAGGGAGACAAGTACTGGAGAGTGAACAAGCAGCTACGGGTGGAGCGGG

GTTATCCTCTGAGCAAGGCTGAACGCTGGATGCAGTGCTACTATTTTGATTAATAACGGGCTAAAGAAAT

ATTTTTAAGGTGCCTCACTGGCTGCAAGGAGAAGTCGCTGGGTGGGACACGGGGGAGTCTGAGGCCTGAT

ATTGTTTCTCATCCATCAGAAAGTCCTGTATTTTCCCTTTATTCTAAATTGGGACACTGGCACAGTAACT

AGGCCAGAAGCCGGGATAATTGTGCGCAGTGGTTACAAGTGAACTGAATGAAGTCTCTGCCATCTGAAGG

ATATTGGCTAGAGAGTCGCACATTGTAGTCTTTACCTTCTGTTATAGAGGCCTGGCTCTGGTCTGTTCTG

GGCAAATGTACAGTGCGGCCTAGAGAGGGGAAGCCCAGTCTGGGGAGAGGGGTCATTTATAAATCAGCAA

TGTCCTACCTCTGCTTGTCTGGTGAACAGTTTGTGGGAGATTGAAAATATTATTTGTGGGAGTTCATGAA

AATATCTTACAGCTGTTACACAATATGATATGTCCTTTGTACTTTTAGCAATGTCTCTTTCTCACAGGCG

CCACCATAGTGCAGGGAGCCCAGGGTAAATGCCATTTCTGTTGTATCCTAAACCTGGTTGTGGTCTTATG

TAACTATAAAAGTTCCATAGCAAAAAAAAAAAAAAA

**15. Xt-MMP 20 cDNA (Scaffold_119):**

CGGTATGGCCATACCAGTTTAATGCAAGATGAGGTATGGCCGCCATGTTTGAAACACTGACCCACTGGGAATACAGTTCTATGGGGTCACATCTCTGGTTAGGGCTGCTGTCATTGAGGGGAATAAGGGGATAGAGAAGGGCCTGATTT**ATG**AACTATTTCAGAGAAACAGACATTCAGCAAGTTTTATTTGGAGGACTAAATATACTGGTTAAGGAATATCTTGACAAGTATTACAGTGACAGAGGAACCATGCGAGTGGCTGAGATGGTAGCTGATGATGTCTCTATGTCAAGAAAGATTAGAAAAATGCAAAAGTTTTTTGGCCTTCAAGTGACTGGAAAGTTGGATCATTCTACGCTCGCAGTCATGCAGAAGCCACGCTGTGGGATGCCAGATCTGGCTAATTATCATGTTTTTCCAGGAGAGCCTAAGTGGCAAAGAAGTTCTTTGACTTACAGGATTACAAAGTACACCTCTAGCCTTAGCGCACAAGATGTTGACAGAGCTGTGGACCTGGGGTTAAAAGCATGGAGTGATGCTGCCCCTCTGAATTTCATCAAAACAACTCAAGGAGAAGCTGACATCATGATTTCATTTGAATCTGGAGATCATGGAGATTCTTATCCCTTTGATGGACCCCGAGGGACTCTGGCTCACGCGTTTGCTCCAGGCGAAGGCTTAGGGGGCGATACTCACTTTGACAATGCGGAGAGGTGGACAACAGGAAAGAATGGGTTTAACCTTTTCACTGTAGCTGCTCATGAATTTGGCCACGCATTAGGCCTGGGGCATTCAAGTGATCCCTCAGCTTTGATGTACCCAACATATAGATACCAGCATCCCATTGGATTCCAGCTGCCAACAGATGATGTGAAAGGGATTCAAGCTTTGTATGGAACTAAGGGAATAGGGAAAGAAAAGCCCATGGCACCTCAACAACCAGCCAATAAACCAGATCAGTGTGACCCAAATCTATCTTTTGATGCTGTTACAGTCCTTGGAAATGAACTTTTGTTCTTCAAGGAGAGGTCTTTTTGGAGGAGACAGGCCCCACTTACCAACATCGGGCCAAGCCCAATCGCAAGTTCATTCCCACAGTTGATGTCGAACGTTGATGCTGCCTATGAAGTCGCAGAACAAGGGACAGCGTATTTTTTTAAAGGACCTCATTACTGGGCAACTAGAGGATTACAAATGCAAGGACACCCCAGGACCATTTATGAATTTGGTTTTCCAAGGCACGTACAGAAAATAGATGCTGCTGTCCACCTAAAGAACTCCAGGAAAACTCTGTTTTTTGTTGGTGATGATTATTACAGCTACGATGAAACAAAGAGGGAAATGGAGGATGATTACCCAAAGAGCATTGATGACGAATTTACTGGTGTGGAAGGAAATATTGATGCAGCAGTTGAAGTTAATGGATTTATTTACTTCTTCTCCGGTCCAAAGGCTTATAAATATGACACTGAAAAAGAGGACGTGGTTAATATTGTGAAATCCAGTTCCTGGATTGGATGCTAAAAACGTTCTGAGAAGATGGTTACAATCACTTCGCATTCATGGGGCAATCGGGTTTTGACATCAACATCAAAGAAGAGTTGGAAATTTTCAAAACACAAAACTATGATTTTAACTTAAAGCAAAATATTTCTTATTTATTAGTTTCGGTTCGACAATTTCTTTCCTTGTAAGGTCATGTTTGCATAGAATATCCATCTCCTGGTTATATTTACATTACTATATACGACTGGGGGTGTTACATTGTTTGCTGTTGTTGTGGTACATTCTCTAGAATGACTTTGTTGATGTTGCCAAGAATTATTACAGTAAATAATAATTGTCC

**16. Xt-MMP 21 cDNA (Scaffold_32):**

CCTCACAGAGTCAGCAGGACAGCAGCAGGACCATCAGCATCACATGAGGCCACC**ATG**CCTTCCATTAAGCTTCTGATCTGGTGCTCCTTGTGCCTGATATCATCCAGGCTGTGCCATTCGGAGAAGCTCTTCCACAGCCGGGATCGGTCAGACCTCCAGCCCTCAGGAATCCAACAGGCAGAACTGGTCAAGGATTTGCTCTCCGCCCAGCAATACCTGGCAAAATATGGTTGGACAGAACCAGTAATTTGGGATCCATCGAGCGCCAATGAAAATGAACCTCTGAAAGATTTCAGTCTCATGCAAGAGGGGGTTTCTAACGCAAGGCAAGAGGTGGCCGAGCCAACAAAAAGCCCTCAGTTCATCGATGCTCTCAAAAAGTTTCAGAAGCTGAACAACTTGCCAGCTACAGGAATCCTAGATGATGCCACCATCAATGCCATGAACAAGCCACGGTGCGGCGTACCAGACAACCAAATGGCAAAGAAAGACACAGAGAGATTGCCCACAAGAAAGTCTTTGGAAAACAAAACCAAAGATTCGGAGAACGTTACTCAACAAAACCCAGACCCCCCCAAGGTTCGGAGGAAGAGGTTCTTAGACATGTTAATGCACTCAAACAAGTACAGGGAAGAACAGGAGACACTACAGAAATCCACAGGGAAAGTCTTCACCAAAAAGCTGCTGAAATGGAGAATGATTGGAGAAGGCTACAGCAATCAACTTTCCATCAATGAGCAGAGATATGTCTTCAGGTTGGCTTTCCGCATGTGGAGTGAAATCATGCCACTGGACTTCGAAGAAGATAACACCTCCCCTCTATCCCAAATAGATATCAAACTCGGATTTGGACGAGGTCGGCATTTAGGCTGTAGCCGGGCGTTTGATGGCTCCGGGCAGGAGTTCGCCCACGCTTGGTTCCTAGGGGACATCCACTTTGATGATGATGAACATTTTACTGCTCCCAGCAGCGAGCACGGGATCAGCCTGCTGAAGGTGGCGACCCATGAAATTGGCCATGTTCTTGGATTATCTCACATCCACAGGGTGGGATCAATAATGCAGCCCAATTACATCCCGCAGGACTCTGGCTTTGAGCTGGACCGGTCTGACAGGAGAGCCATACAGAACCTATATGGTTCATGTGAAGGCCCCTTTGACACAGTTTTTGACTGGATCTATAAAGAAAAGAACCAATACGGGGAGCTTGTTGTTCGATACAATACCTACTTCTTCCGCAACAGCTGGTACTGGATGTATGAGAATCGGAGCAACAGGACACGCTATGGGGATCCATTGGCCATTGCTACTGGCTGGAGTGGAATTCCCGCACAGAATATTGATGCTTTCGTTCATGTCTGGACCTGGACAAGAGACGCCACCTATTTTTTCAAAGGTACTCAGTACTGGCACTATGACAGCGAAAACGACAAAGCCTACGCCGAAGATACGCAGGGGAAGAGCTACCCTCGCCTCATCTCAGAAGGGTTTCCTGGAATCCCAAGCCCCATCAATGCTGCCTATTTTGACCGGAGATGGCAGTACATTTACTTCTTCAGAGACTCACAGGTTTTTGCCTTTGATATCAACAAAAACAGAGTTGTGCCAGACTTTCCCAAAAGAATCATCGACTTTTTCCCAGCCGTGGCAGCCAACAATCACCCCAAGGGTAACATAGACGCGGCGTACTACTCCTACACACACAGCTCCTTATTTCTCTTCAAAGGAAGAGAGTTTTGGAAAGTCGTCAGCGACAAGGACAGAAGGCAAAACCCCTCTCTTCCATACAATGGATTGTTCCCCAGAAGAACAATATCACAGCAGTGGTTTGACATCTGTAATGTACACCCTTCATTGCTGAAAATGTGATTCGCCCAAAAAAGCC

**17. Xt-MMP 23 cDNA (Scaffold_414):**

AAGGTGCAAGAAAAGAAATCATCTTTTGGACTGGTCAGTATGCATATCATTGTAAGGCAGGCAAACTCAGACCAAGGGGCAGCACACAGAGAGAAGTGACTATAGGGAGCATGCAGTGCATTCCCCCAACAAGCTGACTGAGAGAAGGTGTCCGCTCAGCCTCTGCCAAGTGCATCGCCTATCACAGACTTCTGATAAAACC**ATG**GGTGACATCCAGGAAATTGAGCATTGGCAGAAAAGGTACATCTGGACATTTCTTGCCATTTTTGCTGGAACAGTGCTGGTAGCTGGAATTTTCACAGCATCTAATTCAGTGTCCTTGGACTCAAAGGTAGACTTTGTTGTGGCTCCAACCCCGGCTCTTCAACTGCCCCTTCAGCTCCCTCGCCACCTGCGGAACAAGCGTTACACATTGACTCCAGGTCTTCTGAAATGGGATCATTATAACCTGACATACAGGATTGTTTCATTTCCAAGGAATTTGATCAATGAAAGTGACACAAAGAAGGGGATGGCCCAGGCTTTCCAGATGTGGAGTGAAGTGTCTCCTTTTCACTTCAAGGAAGTCCCTGCAGACCAACCAAGTGACCTAGTAATAGGTTTCTATGGTATCAATCACACAGACTGCTTGGAGTCCTATATCCATTATTGCTTTGATGGAACAACGGGCGAGTTGGCACATGCGTACTTCCCTAAAACAGGAGAAATACATTTTGATGACAGTGAATTTTGGATTTTGGGTAACACGAGGTTCAGTTGGAAGAAAGGGGTGTGGCTCACAGACTTGGTTCACGTGGCAGCCCATGNNNNNNNNNNNNGCAAGGATAAATATTTAGTGTGTGCCTCCTGGGCCCACAAGGGTTACTGTGACTCTAGAAAGAGAATTATGAAGAAGTATTGCCCTTACAGCTGTGACTTCTGCTATGACTTCCCATTTCCAACCAAACTGCCTACACCTCCACCTCCTAGAACGAAAGTCCGATTGGTTCCTGAAGGCAGAAATGTTACTTTACGGTGTGGGAAAAAAATTATGCATAAAAAAGGAAAAGTTTTCTGGTACAAAGACAAAGAGTTGCTGGAGTACTCTTACTATGGTTACCTTAGTCTCGATGATGATCACATGAGTATAATTGCCAATGCCATAAATGAGGGACTGTACACCTGCATTGTGAAGAAGAGGGACCGCATTCTCACAACTTACTCATGGAA

**18. Xt-MMP 24 cDNA (Scaffold_954):**

GATGCTGATAGGTGGAACATGATCCCTGGCTGCTGTGCTCCAATTCCCCTTCCCCGTATCTAATATCCGTGTGCCCCCCCCGGAGACAGGAGCGGTGCTGCTGCAGGTGCCCGGGACCAGGCATGGCATTGGGGGTGCCCAGAGCGGCAGGTTTAGCCTCTCCTCCCAGCCCTGCGCATCACTCTGCCTGGCTCTGCCCCCCTCCTCCAATTGGCGCTCGGTGCGACTGTTACTAAGGCAGTTTCTCTGCTCCCCCAACAGTCGCAGCCGCCGCCGCCGACCCTGCCGCCGCAAGTGCGAAGCATCGCAGTGCGACCAGTCCTGCCCGACTCCCCGGCTATTGTTGTGTGGGTGCGGGGATGGGCACAGCCCTGGCACCACTCTCCCCTGGAATCCAGCCTCCTGAGACCGCACCCTCACTGCTCCCCCCCAGGCTGGGTGTAATGCCTCCCAAATAACCCCCCTACCCATCAGCACCACCACCTGGGGGGGCAGCCTGCCCTGCCTGCCGCTCTCTGCATGTGATGCCCATGTAGTGCCCCCTTCCAGCCCTCCGTG**ATG**GCACAGACTGGAGTCAAGCGACAGAAAGCGCATGTTCTGGGAATCTGTATCAAAACCTGCTTTTTGCCCTTTTTTGTGGTTTCAGTCTGTGGGGAAGAGAAGACCTTCATAGTGGAGCGCTGGTTAAAAACATATGGCTATTTGCTTCCGTACGACATCAGAATATCCGCCTTACAATCAGGGAAAGCTATGCAGTCGGCCGTCTCAGCAATGCAGCAGTTCTACGGGATTCCGGTGACCGGAGAGCTGGACCAAATGACCATTGAGTGGATGAAGAAGCCTCGCTGTGGAGTACCTGACCATCCTCATTTAAGCCATAGAAGAAGGAATAAGAGATATGCACTAACAGGACAGAAGTGGAGGCAAAAGCACATTACATACAGCATACACAACTACACCCCTAAAGTAGGAGAGCTGGACACCAGGAAAGCCATCCGCCAGGCGTTCGACGTGTGGCAGAAGGTGACTCCTCTAACATTTGAAGAAATCCCCTACTCAGAGATCAAAAATGAAAGGAAAGAGGCGGACATCATGATATTTTTTGCTTCTGGTTTCCACGGAGACAGTTCCCCGTTTGATGGAGAAGGCGGATTCCTGGCGCACGCTTACTTTCCCGGCCCGGGTATAGGGGGAGATACGCATTTCGACTCTGATGAACCTTGGACTCTTGGGAACTCCAATCACGATGGAAATGATCTGTTTCTAGTTGCAGTCCATGAGCTTGGCCATGCCTTAGGGTTGGAACATTCAAATGACCCCAGTGCAATCATGGCTCCTTTCTACCAGTACATGGAAACTCATAATTTCAAGCTTCCCCAGGATGACTTACAAGGAATCCAGAAAATCTATGGCCCCCCTGCAGAAACCATGGAGCCGACCAGACCTCTCCCCACTCTCCCACCACGACGGATCCATTCCACCTCTGAAAGGAAACATGAAAGGCAGCCAAGGCCACCACGACCGCCACTCGGAGACAAGCCACCCAGTACAGGCTCCAGGCCAAATATATGCGATGGAAATTTTAATACCGTGGCTCTCTTTAGAGGAGAAATGTTTGTTTTTAAGGATCGTTGGTTTTGGCGCCTTCGTAACAACAAGGTACAGGAAGGTTACCCGATGCAGATTGAACAGTTCTGGAAAGGACTTCCACCTAAGATTGACGCAGCTTACGAGCGGTCGGACGGCAAGTTTGTTTTTTTCAAAGGAGACAAGTACTGGGTGTTTAAGGAGGTCACCGCCGAGCCAGGTTACCCCCACAGCCTAGTGGAGCTGGGAAGCTGCCTCCCGAGGGAAGGTATCGACACGGCCTTGCGCTGGGAGACCTTTAGCAAAACCTATTTCTTCAAGGGAGACCGATACTGGAGATACAACGAAGAGAAAAGAGCAGCAGATCCGGGGTACCCCAAGCCAATTACAATATGGAAAGGAATTCCTGAATCACCTCAAGGGGCTTTTGTGAGCAAAGAAGGATCATACACATATTTCTATAAAGGGAGAGAGTATTGGAAATTCGATAACCAAAGGCTAACCGTGGAGCCGGGCTACCCCCGATCAATTGTTAAAGACTGGATGGGCTGTAACCAAAAAGAAGTCGACAACAGCAAAGACCGCCAACTGCCTCACGACGACGTGGATATTATGGTCACCATCAACGACGTTCCCAGTACTGTAAACGCTATAGCCGTGGTCATCCCATGTATATTGTCCCTGTGTATCCTCGTCTTGGTATATACTATATTCCAATTTAAGAACAAAGGGGTCCAACAGAACGTAACCTATTATAAACGCCCCGTGCAGGAGTGGGTATGACAACGACCCGGAACGTTTTTTTGAATTCTTTTTAGTTTTAATGAGGGCTAACAGGATGCTGGGATCAGAAGTCCATCGGTCAGACTCTACCGCAGATCAGTCATCAACGCAACGATCCCAGTGTTACCAGTAAGGAGGGACACCGCCAATACGGAGGTGGCCTTAAACAGTGGAACTTCTTCCATTCTTACTGCCTCATACCTTTTTCTAGTGTGCCATTTTTAAGGATCACGTTGTGAGTGGGAGATTCCAGGGCTGGGATTTCTTCGCTGAACTGGAAGGAATTATCAGGCCCGAGAACACATCTCCAAACCAGCTTTCTCCACCGACGTTAGATATTTCCTACTGAACTTGTTTTTACGGACTCGGGCGGAGGAACGAGACTCGACGCGTTTCTCCGCCAGCGCTCAGAGCGATCACTGCAATAGATCTGAGCAAGGAACAATGAGGCGCGGGTCCTTTTTACCCTGAGATAAGGCACAAAGACTGGCGCATCAGGGAACCAGATGTTTTTAAATGCACAAAAATAAAAAAGAAACTCCGATTTTTTTTTGTTTTTGTTTTGTTCAATCCCTGCTGGTCAGAATTATTACATACAACGGTTGTTAAATCTAGATTATTGAATTGGGAATGAACTGCTTGCCTAAAGAAATTGTCATGAGAGTCACCAAACGATGTCTGTCTTTCGGGGGGGGGTTTCCGTCTCTTGAGATCAGAGAATAGAGAAATTTAAAAAAAAAAAAATCATATTCAACCCTTTGACGATATCTGGTTCATGTTGTATTCATGTAGAAGCAAGAAAAGCGAAATAAAAACTGG

**19. Xt-MMP25 cDNA (CU075461):**

CACATGGGAGATAGCAGCGCTCGGAGCCTACAAGGAGCAGGCAAAGGAGAGGAGCAAAGCCACACACAAC

TCTGTGGATATTTTGGGAATTGCGCAACTTTCGCGCAACTGCAGACTGAGAGACCCGGTAAGGTTGGAGA

ATCCAGTAAAGATCGCAGTGTGGAACAGGCGCAGAGGGACCTGCCGAG**ATG**CTGGCGCTATGGGTGCTGT

GGGCGAGCCTGGGACTGGCCGGGGGGAACCCCAGCGCAAGGGACATCAGCAAAGGAGTGGACTGGCTGAC

AAGATATGGCTACCTTCCTCCTCCAGACCCTTTTTCAGCTCGGCAGCAAACGCTGGAGGGGCTCCGCGAG

GCAGTCAAGGCTATGCAACGAGTAGCGGGCCTGCCAGAAACTGGGGAACTGGATGATGCCACCGTGCGCA

TGATGAACAAGCCACGATGTTCCCTTCCTGATATCATCATGAGACCCGAGGGCCGCTCTCGTCGCAATAA

GAGATATGCTCTAAGCGGCTCTGTATGGGATAAAAAGTTGCTGACTTGGAGGTTGGAAAATTCCCCCAAT

ACTATGTCGCAAGATGTCTCCCGCACCCTGATAGGCACTGCTCTGGCAGTCTGGAGCAAAGAGACACAAC

TGCAATTTAGAGAAACGCGAGAGCAGCCCGACATTCGTGTAGAATTCGTAACTGGGTCCCATGGAGACGG

CTATCCCTTTGATGGTCAAGGTGGAACTCTCGGTCATGCTTTTTTCCCAGGTGTGGGGGAAAGAGCTGGA

GAAACTCATATGGATGCGGATGAATCTTGGTCATATAATACTGAGGAAGGTACAGACCTCTTTGCGGTGG

CAGTTCATGAATTCGGACACTCTCTGGGGCTGTATCACTCTTCCAGTGAAAACTCCATCATGAAACCTTA

CTATCAGGGCACTGTAGGGGATCCTAGCAGATACAGACTGCCTCCTGATGATGTGGATGGGATCCAGACA

CTTTATGGTCAGCCAGACTCTGGGCACAGACCTCCTGTTACACCAACCAGGCGTGCCCTTCCCCCACGTG

GGCCCTCTCCGGGACCCCGTCTCCCATTCCCGGATCGCTGCTCGACCAACTTCGACGCCATTGCCAATAT

CCGAGGAGAGGCATTCTTCTTTAAGAACAGGTATTTCTGGCGGGTGCAGTCCTCGAGGCAGTTGGTCTCA

CTCAACCCAGCTCACCTCAACCGCTTTTGGCTAGGGCTCCCTCCTGACCTGCCCAAATTGGATGCTGTGT

ACGAGAGAACCAATGACAGCAAGATTGTGTTTATTTCAGGAACCTCATACTGGGTTTTCAAGGACACCTT

GGTTGAGCCTGGATACCCTCGTCCCCTGGCCGACTTCGGACTGAACACGGACGGCGTGGATGCAGCTTTC

GTTTGGAAGCACAATGGGAAGACGTACTTCTTTCGCAAAAATCTATTCTGGAGGTTTGATGAAAAGAGAG

GACAGATGGACTCGGGGTATCCAAAGGACAGTAGCTTATGGCAAGGTGTGCCACCCAACATCGATGACAT

AATAAGTTGGGAGAATGGTGACACCTATTTCTTCAAGGGCACGCAGTACTGGAAATTCCAAGGTGGCAAT

GTGGAGGCAGATCCAGGATACCCACGGAGCACAGCACTGAACTGGATGTACTGTCCTTTGGAAGGCCCCG

CTGTTCCTACGGACGCCCCTGAAGGCAGGGACCAGAGAGGCTGCAGCTGTACTTGCACTGATGGGAGGAA

TTCGGCCTCGGAAATCGGCATCCTTGGGGCAGCATGGCTGGCTTTTGCCTCTCATTTGCTGCGGTTTCTG

TTCTGAAAAGAACAGGCAGGACAACTGCCCTAGTAATGGTGCCCGGTGGGGTTTGTTTTGTTTAAAGAGC

AGCCACACACGGGAATGGGCACAGCATGGAAGCCATGGTACAAATGTTGCCAGCAAGACAACTGCAGATG

ATGAACTGATGGGACTCTTATCTGTACTGAGCCCAGTGCATCTGCAGTGACTAACACAATCTCCTGGTTT

TGCCCATTCTTTTATTGACAGAAAGGATTTCTCTCGCTGTACAGATTACAAAAAAAAAAAAAATTAAACA

CAAAAAAAAAAAAAAAAAAAA

**20. Xt-MMP26 cDNA (NM_001032335):**

CC**ATG**CTACAGGCAATCGTTTTTGCTATCTTGGCCCTGTCCTGTATCCACGCTATGCCAGCACCTATCAC

TGACAATAATATCAGTCCATCGGACCGTCAGTTTGCCGAGGGTTACTTGGACAAATACTACTTGATGACA

GCAAAATCAAAGAACACCTTTGCGGAAAAAATCAAAGAGATGCAAAAGTTCTTTGGGATGTCGGTGACAG

GGAGGTTGGACTCGGACACCATGGCAATGATGAAGACCCCCCGGTGCGGAATGCCCGATGTTGCAGAGTT

CAGACAGTTCCCTGGGAGGCAGAGATGGACAAAAACCCAGCTGACATACAGAATTGTAAATTACACTCCT

GACCTTCCTCGCTCAATGGTAGATGAAGCAATACGGTTGGCATTTAAAGTGTGGAGTGATGTGACCCCAC

TAAAATTCACACGAATTTCCTCCAGACGGGCAGACATAATGATCCAGTTTGGAGCCCGCTCGCATGGAGA

TTTCATCCCATTTGATGGCCCTAATGGAGTTCTAGCTCATGCCTTTGCTCCAGGTAGTGGCATAGGAGGT

GATGCCCATTTTGATGAAGATGAAAGATGGACCAGTACTAGTGCAGGTTTCAATTTGTTCCTTGTAGCGG

CACATGAATTTGGGCACTCGCTGGGATTAGACCACTCTAGAGACCCACGTGCGTTGATGTTCCCAAGCTA

TCGTTATATGAACACCAGGAACTTCCGCTTGCCACAAGATGATGTCAATGGAATACAGTCAATATATGGG

AGAAAAACTGGCAAATAGTACCAGTTGGGTTTTTATGTGAAGGTGTTAGATCAACATAAACATATGTTCA

GTGCTTTAGCATGATGATCATTTTATTCTTATATCAAAGGAGAATCTAGATGCCCAAGCCTTAGCATATG

TTACCTGTGGCATTATGTTCCGCTTTATCTACTTGCAAACAGAACCTTACTGTTGAGATGAATAGAGGGA

TCTGTTACAGTTTGCAATATAATGTTACTGCAGGGGACACATTTGCTGTAGATAACTATAAAGTATTTTC

CTAGCAACCTGAAGGTTGGAGAGCATATACTGAAACATGCAAAAGCCTCAGTAGAAAGGTACTTAGGAAA

TACTCAAAAAAGCACAGATGCACTTTCATCTTGGCAATGTGGATAGAATGGCAGCTTATTAGTTTTAAAG

GGTGCACGTGGTTTATCAGATAAGGATTCTGGCATTGGTTCATCCAAGATTTGCCTGAAGTGAAGGTTCT

ACACCAAGACATTGGCCCAAAGTCTTCTCCTCACACTACTGCCAAGACCCCAGAACAGTATTAACTCAAA

TATTTTGCAGGACATTTGTACAAAACACATGCATATAGAATTCTCTGGGCAGTTACCCGAAAAGACAGCC

AATGATTGTAGCCATGAATACTTAGAAGTACTTGGTGGTTTGTCTACTGTGCTACTCAATAGGACATATT

TATTATAGTGTGTACGCCAACATCAACGGTGATGTTGCTCCAGGGTCGGACTGGGCTGTGAAGGGCCCAC

CGGGGCTCCCGTCCCAGGGGCCCTGCAGGTGCACTCAGCCAGGCGTGTCCTCAAATCCCCCCCCAGGGGC

CCCCTTAACCCCTCTTGCAGGGCCCCCACCCGGCGTCCTCCCCAAGCGCATAAATTTGACACGTTGGGGC

AGAAGCGTTCGGGAGAGGGAGCGCCGGCAAGGGTCGGGTCTGGGCCGCCGGGCCCACTAGAGCCGGGGCC

CACCAGGATTTTTCCCGGTGTCCCGGCGGCCCAGTCCGACCCTGTGTTGCTCATAGCAACTAATCAAATC

TTTGCTTTTGTTTTCTAACTAGGTTGGTGTCCATTTAAACCTAATAGCTGATTGGTTGCTATGAGCAACA

TCATCGTTGGCTAACACACTATAATAAATATACCCCAATATATACAATGATGTAACCAGCAATACTGACC

AAAGAAAAGCCATGTTCAACAGTCCAAGACGAAATTGCCGCATAATTAAATACAATAGCATGCAATATGA

AAAAGCTCCAGCAGGTTGTGTACCAATCACATGTTTTTTTTTTAAAGGTAGATTTTATTTGTTTTACAAA

ATCAAAACAAAACAACAAAAGTAGAACATGAATGTTGCTATACAGTTTGTTACAATAAAATAAGAAGTTG

TTTAAAAAAAAAAAAAAAAAAAAAAAAAAA

**21. Xt-MMP 28 cDNA (Scaffold_72):**

TGCCTGGCTGAAAAGAACAGCTGAAGTCTCTGATAACTCTGGCATGCCAAACTCTGCATGCAAACACTTGGGAATTAATATCACAACAGAAACAGGGCAGAGAAGCACACGAATTTAATTCAATTGTAGAAGATGGATTGGAGATAGAATATTGATGCAGAACGAATGACACCAAACTGCCGTTGCCTGGAAGTGTTGAAGAAGACACAATGATTTTGCTAAACCATTCAAATCAAACATTTGAAGAGCCTTGTTCTGACTATATGAAAATAACTTTACTTCTGACTTTTTAACTGGCTTGCGTGTAAGCTGTACTTTAGATGGCTGTGCATCATTACATTGTTTAATGGATGAGTAACTTCTCTAGTGCCTGCAATTTCCCCTTGAAA**ATG**GAAGCTAATATTCCACCCCTGGTCTTTCTGCTTGTGACAGCTGGTCTGTGTTTATGCAATGGCCACATATCTGAAGAGACCCTACAAACTGCACAGGTGTTTTTGGAAAAATATGGTTACCTTAAAGAAACAACTAAACATCATAGTGGCAAACAACTTGCATCAGCAGTCAGAGAGTTTCAGTGGCTGTCACACCTGCCTGTCAGTGGAGATCTGGACTTCTCTACAGTAAAACAGATGATACAGCCTCGATGTGGCATGAAAGATGATGAATCGCTAGAACTGGTCAAAAGTCACCGTCACCGTCAACAACGCAAGAAACGTTACATTTCCAAAAGTAAGAAATGGTACAAGCAACATTTAACTTATCAGATTGTCAACTGGCCCTGGTACTTATCCCAGCATCAGGTCAGGCAGGCGGTAAAAGCAGCGTTCCAGTTGTGGAGCAATGTTTCCTCTCTGACATTTTCTGAGGCTCTAAGAGACCCTGCAGACATCCGTTTGGCATTCTTTCATGGGGATCACAATGATGGTGCTGGAAACGCTTTTGATGGACCAGGTGGTGCCCTGGCACATGCTTTTTTCCCCAGAAGGGGGGAGGCCCATTTTGACAGTGCTGAGCACTGGTCATTGAATGGTAAAGGCCAAAACCTGTTTGTTGTATTGGCACATGAGATTGGACATACTTTGGGACTTCAGCATTCTCCCTTCAAAAATGCCCTGATGTCCCCTTACTACAAGAAACTGAATAAAGACTATGTACTGAACTTTGATGATGTCCTGGCCATCCAAAATTTGTACGGAGCGCCACCTAGTGGAAATCTTGTGCAACTCCCTGGAAAACAATTTGCCTTTTTTCAGGATTGGAGTCCAGAATCACATGAAGATACTAATTCAGGAATGAAACAGTCTTCCTACTGCCACTCTATCTTTGATGCCATCACATGGGATTTGAAACATACATTATACATCTTTAAAGGTCGTCACTTTTGGACAGTCTCATTGGATGGCAAAATCTCACCACCCCAAACTCTTCAGATGCGCTGGAAGAAACTCCCTTCTTATATTGAAGCTGCAGTTGTGTCTGGTTTTGATGGAAAATTCTACTTCTTTAAAGGTGGAAGGTGTTGGAGGTATAAAGATTCCATTTTAGAAGAAGGTTTTCCTCAAAAATGCAGCATGAGCGGTTTGCCTCGTCGTCCTGACACTGCACTGTACTTTCAGCCTCTTGGTCACTTAGTCATTTTCAAGGGCTCTAAATATTATGTTGTAAATGAAGAGTCCCTAACAGTGGAACCTTACTACCCACGGAGCTTGCAAGACTGGAAAGGTGTGCCAGCCAACAGTCACAGTGTTCTGACCCATCCTAATGGAGCTGTCTACTTTTTCAAAGGTGACAAATACTGGATATTTGACCAAAACAAGCTAAAAGTAACTACTTCTGGAAAGTGGGCTGAAGATTTGTCTTGGATTGGTTGTAAAAAGCATAAGACTTGATTTTTCTACAATGTTCAAATGGCTTCACTTGT

**22. Xt-MMP N1 cDNA (Scaffold_501):**

AGTTAGGTCAGTGTTTCAGTGTCCTTCTAGGGCATCGTAAAGTGGTAGTCCACCTTTAATTTCACTTTTTTTATTATTTAGACACTGATATTCTGAAATCATTTTCAATTGGTCTAAATTTTTGTGCATGTGTGTTGTTGAATGATTTGATTCAAGTCTGATCAGGTGGTGGAGTTGCTGGAATGTGGAGGCTTATCTAATTAGTGGCATTTATGTGCTGCTGCTGCTCCGTGGCCATGCATAAATGCACATTTGCAGGCCTGACTCTGCCCCGGGTCCATTTACCCTCCCCTCTGGTGACATCAGCAATGGGCAGGCCGGCTGTGGGTTGGCAGAGGGTGGGTTTGGGTCTGGTGTTGTTTCTTTATTGGCCCGTTCATCACTAGTAGGGAGCCAATAGCATCTGTAGTGGGATGAGACCTGTAAAGCTTTATCCCTTTTGCTCTGGACACGTTGGACATGCCTGAACTCTTACACAATAATATATATTATTTCTTCCATCGTCACCCTGGGAACACCTGTGATGCTTTAAACCAGCGCAAATACTCTCTGCCCCTCCATAAAACCAGCCCTGCCCATTGGGGTCACTTTGGCTTAGGCAACATGGGCTTTTTGATCACCAAATACAGGGAGCTGCATTGGTCAACTCTCATTAAACTGAAAGGCAATCACTGCAACCCGTGCAATAACCCATAGCGAACAATCATGGATTTGCTTTAGGGCTTATTTATAATGTTAAGCAAATCTTTACCAGGAGAGTAAAACACATCTATATAAGAGAGTCAAAATCTATATTTGTATCCATTATCGGTTTATGCAGTGTAAAACCCTAGTGAATAGTTCATGTAAATCTTGGATCCTGGTGTCACAATCCCATAGCTAAAGGCAAATAATGTTAGAAACCTCTGTGATCTTCAGTGGAGCTTTTTCCCTGCAAACAGCCAATATTTATGTATCGTAGCCCCTTTGGTACAGCATGTGTTTTATCTTTGGATAAGCCAGGAGGTAAGGGGTCAATACCCTGTGTAATTCTAGCGTTCTTGCTGAAATAACAAGGCCTGTTTGCACAGGTTTAATGGATAGCTAGGGGGGAGCACAGTGTCCCACTGAGAGTCCCCAGGTACGGCCACAGAGCAAGGAGCACC**ATG**GCCACAAGACGTGTGCCACTGGCTTTAGCCTGGGCAGCCTGGCTGCTTGTTTTGCCACTCAGCTTCTCTAGACCAATTGTGGAGGAATCTAATAGTGGGGAATACAGAGGTACCCCTTCTCCAGAGGTCAATGTCCCCCCAAATATGGACCCTCGTGGTGTAAAACCAGAAGGACCACTTCAGATAGACACAGAACAATTTAAATTGGCAGCGGATTATCTGATAGAGTTTGGGTACCTTCCGGACGAGAACTTTACAGAATATTCTGTGCCCAACACTCAATTTTTCTCTGAAGATGAGCTGCCCGAAGAGTTTATCAGCGGCTTGGAGTGGTTCCAGAGACAGAATGGCTTGAAAGTGACTGGTAAATTAGACACAGACACGGCTGAAGCAATGAGGCTTCCCCGCTGTGGGAAACATGAGCAACGCATGTCATACAATCTGGGCTCTAAGTGGAAGAAAGATATGCTGACCTACAAGATCCTTAATACGACAGCCCAGCTGCCTGAAAAGATGGTCAAGGATGAGTTAAGCAAAGCCTTGAAGGTCTGGCAAGATGTATCTTCTCTGAAGTTTGTAGAAGTTGGGATCAATGAAACAGCAGACATTGATATGTTCTTTGTGTCTGGCCTGCATAATGATGGCATTAAAAATGCTTTTGATGGTCCTGGTCGTGTTCTGGGTCACGCGTTCATGCCTCCGTTCAGTAAAAATAAAAAGGACATCGATGGAGACCTTCATCTGGATAATGATGAGAAATGGACAATTAATGAGAAGAAAGGGGTCAACCTTTTGCAGGCCGCCGCACATGAACTTGGGCATGCCCTGGGACTAGACCATTCCACTGTAACAGGAGCTCTCATGGCCCCAACGTACAAAGGCTACAACCCAAAATTCCAGCTTCACGAGGATGACATTCAGGCCATCCAGGCTCTTTATGGCAAACCAAAGCTTAATCAGACCACAGCTAGTAATGCCACCGTAATCAAGACTGGAGATGTGAAGACAGATACAACATCCAACACAAAACCCAAAGCAGAAGACAAGGATCCCAAAAAGCAATCGGTGGTGAAGATGTGTGGGCAAGAACCAATTGATACATTTGTATCCACCAAGAATGGATCTATCTTTTTATTTAAAGGTGAATACTTCTGGGAGATGAGCCACGGGAAGCTGCCATTAACAACCAAGAAGAAATACCCCCAGCTTATTTCAACAAAGTGGAAATCTTTGCCTTCTTCCATTGATGCAGCAATAAGAATGCAGAACCCATCTGCAGATCAAGATGGAAAGATCTTCTTTTTTAAGGGAAGTAATTACTGGAAGTTTGACAATGGTCAAATGGAACCCGGATACCCAAAGTTAATCAGCGAAGGGTTCCCAGGTGTCCCAGACCATCTGGATGCGGCATTTACACAACCTGCTATTGTAGCAAAAGGAGGGAAAGTTATAAGAGAAGAGAGAATTTTCTTCATAAAAGGAAAAAACTTTACTGTGTACAACCCTGCCACTGGGAATAGTACCAGCCCTCAGTCTCTGCAGGATGATTGGGTGGGAGTCAAGCTGCCAATCACTGCAGCGCTCAGTCTGAAGAATGAGATGTTCCTCATCAGCAAAAAGAAGTTCCAGAAGGTTCTCATGCTGACTTACACTCAGGATCACGTGTACGGCAACATACACCAGCCCAAGAATCTAAATCAGCTGCTTCAGTGCGACTGAACTATAACACTGAGCTCTGTCTGCACAGATATATGTAAATAATTCTACCTTACTGAGTCATGCACACTCTTACAATATTCTTTTATTACTATATGTGAGATCGATTTATCTCCTTAATCATGACATCATATGTAATATCTCATGAATCGCTGAATACATAATGATTGTATGATTAACCATTTTATGTTGGGAGGGAACAAAAAATCTAACCACCCCACCCTCTTTTGGTTTAGGTGTGAGCCCCCTTTCCACAACCTGCTGCACAGGTTGCCAGAAAAATAGTAACATTTTTCTATATGTTTCATATTTTTATCTTTATTTCCTATTAATAATATTGGCATGAAGCATCATTTTTAATGGCCAGAAAGCAACCCCCAGCTGCAAAGCTCCCAGTGTGTAAAACCCCACAGGTCCCCCCCATACCTGGTCTGCAAATGCAGAGTTGTGTAGCAAGGCTGTCAGCTGCCATGTCACATCCAGTGTCTCTGTCCCATTGCAGAGCAGTCACATATGCACTGAAAGCCAATTTGGTTGGTTAAATCTGGGGTCTGGTTTCCAGCCAAATATGGCACCCAACAAACCTGCCACACAGGCACAATGCCATCCTAAAGCCGCTGAATATTACCCGGGCATAAAACTGGGTAGCTGCTAAAAAAGGGGGGGTTGACAGCCCTGATATTTACAGGCTGAATGGATGCTATGTGATTGGGTAGGGGAAGGGGCATTCTAAGGTATCTTACTCTTAAAAATGTTCTTAGGGTTAAAATAAAGATATGGTATG

**23. Xt-MMP N2 cDNA (Scaffold_119):**

**ATG**CTACAAGTAATGTTCTTCGTTTTCTGTTCTCTTTCCTACAGCACTGCAATGCCAACACCCCAAAGTGAGGATGTCATCAGCCAATCAGACTACAAGTTTGCTGAGGATTATTTAGGAAAGTTCTATCCGATGAACCCAAAACTAAAGGCAAACGTATTTGTGGAGAAAATCAAAGAAATGCAAAAGTTCTTCAGAATGACAGTTTCGGGGAAGCTGGACAGAGATACTCTGGCTATGATGAAGGCTCCTCGATGTGGAATGCCCGATGTGTCAGAATACAGCAAGTTCCCTGGACATCCACGATGGAAAAACACCAAACTGACATACAGAATTCAGAATTACACCCCTGATCTCCCTCGCCAAAAGGTAGATGAAGCGATACAAAGGGCATTCAAGCTGTGGAGTGATGTTGCCCCACTGACCTTCAGAAAACTAACTTCGGGAACAGCAGATATCATGATCAAATTTGCAAAACGCTCACATGGAGATTTTGACCCTTTTGATGGCCCCCATGGTGTTTTGGCTCATGCATTTGCTCCAGGAAATGGAATAGGTGGGGATGCTCATTTTGATGAGGATGAAAAGTGGACAAATAGTGCAGCAGAATATAATTTGTTTCTTGTTGCCGCACATGAATTTGGTCATTCGCTGGGTTTGGGTCATTCTCGTGATCCAAACGCTTTGATGTATCCAACCTATCGCTATTGGAACACCGGAAACTTTCGTCTACCCCAAGATGATGTGAAAGGAATACAGTCCATATATGGGAGAAAGAAATAGGCAAAATAACATGAGCTGGATCTCCATATTGTATTTGCCAAACAAAATCAGTATGCAATGCTTTGTTATTTTAGTATGGCAATAATCCTTCAGCAGCTACACAAAGAGAATGCAATAATAACCTACATTTAACCACACGGGAGGGTTCTAGTTGGTCATGGCATGAATCCAGCTTTCCAAACAGTGATTGGCCAACGTGAGAGTGGACCTTGGAATCGGCAAAGGTTTAAGACATCTTCTATGGACCCCACTGGTCATAATGATATTAATTACTAATTGTTTGCTTCTGTTTTTC

**24. Xt-MMP N3 cDNA (Scaffold_508):**

**ATG**GAGTCCAGAATAAGGCCAAGTTTGGACCGAATGGAGCAGTTTGCTCATCGAGGGAAGATGCCAGTCACAGTCGCCATAATCTTCCTGATGCTGATCGCAGGATGGAGTGACTCGGAGAAAATCTTTCATAACAGAGATCACTCAGATTTTGATGTGCAGAATATACCACATGCGGGTGCGATCCTGACCCCCGAGGCTGCCCAGCAATACCTTGTAAAATATGGCTGGGTTGCTCCTGTAAATTGGGAGGAGCAAGCGTTTACAGATTTCCCTGTACATGATTTGGCACCACAAGACGTCTCCCAGCTGATATCAGAGGGAGAATCTGAGGAGCATCCTAGAGTCAGTAATTCAAGTGAAAATGAAACTGAGATAAACCCTTCCTTCATAGACTCCTTGAAAAAATTCCAGGATGCAAACGGGCTAACTGCTACAGGAGTCCTGGATAGTGCTACAAAGATAGCCATGAACAAGCCACGCTGTGGTGTTCCTGACTTCAAAGTTGCATCAAGACGCAAGAATGCAACTCTAAGTTTTACGGATGCAGACAGCAACTCTCACAATAGCTCACAAGCTGGTGCAACTCAACGGAGGAAGAGAAGTTTCCTTTCTAAAATGGTTGAACACTACAGGCAAAAAAGAGAAGCCAAAGACACCATGGGAAGCAGCAATTCCAAACGGTTTTCAAGGAATACGGTGAAGTGGAGGCTAATGGGAGAAGGGTACAGCATGCAGCTGACCATACAACAACAGAGGGCAATTCTTGCCTTGGCTTTCCGTATGTGGAGCGAGGTTGTCCCTCTTCTATTTGTGGAGGACCTCACAGGAGATGATATAGATATTCGAATTGGCTTTGGAACAGGACAGCACCTTGGCTGCTCACAGGCATTCGATGGGGTGGGGCAGCAGTTTGCTCATGCCTGGTACCTCGGGGATATTCATTTCGATGATGATGAACACTTTGTTGGACCATCCAGTGAGCATGGAATCAGCCTTTTAAAGGTTGCGGTGCATGAGATTGGCCATGCCCTTGGACTGAGCCATATAAACCGTGAAGGATCTGTCATGCAGCCAAATTATATCCCTCAGGAGCGCCATTTTGAGCTTGACTGGGAAGACAGAAAAGCAGTACAGGAAAAATATGGTGCCTGTGATGGCAGCTTCAGCACAGTTTTTGATTGGATAAGGAAAGAACGGACTCCACGCGGAGAAACAGTCTATCGATTTAACACTTATTTTTTTAAGACAAATTGGTACTGGATGTATGAGAACAAGAGCAATCGAACACGATTTGGGGACCCTCTTCCTATCAAAGGCGGCTGGAATGGTATTCCAGATAAAGACATTGATGCCTATGTTCATGTATGGACTTGGAACATTGATGCACAGTACTTTTTCAAAGGCACCCAAGTCTGGCGCTATGACCCTGACAAAGACAGGGCATTTACAGAGGACTGGAGGAAAGTCAAATATCCCCAGCCTATTGCCGACGTGTTTCCAGGAATCCCAAGTCCAGTGGATGCGGCTTATTTCAACAAGAAGGAGCGTTTCATCTACTTCTTCAGAGGAAATAATGTTACTGCCTTTAGTGTTGATAAAAATCAAAAAGTGGAAAAGTTCCCAAAATCCATCATTGACGTATTCCCACCTGTGGATCCCAATGACCACCCCAGAGGAAATATCGATGCTGTCTATTTCTCTTATTCCTACCAAACAACGTTCTTCATCAAGGACAAGTATTACTGGAAAATGGTTATTGATCAAGAAAGACAAACCAACTCCTCTCTGCCTTTGAATGGATTATGGCCCCGCAAGAAAATCAACTCCCAGTGGTTTGATATTTGTGATGTCCATCCTTCTGTACTCTTGCTGTCAACATAAGGAGCAACGGTTAAGGCTTCCATCAGAAGGACTGTATAGACTTGATGTCTTTGAATACATTTCACCTTCAGCCATCCCTGTGGACAACCAACAGACTGAGGACTTCTGCTGTGCA

**25. Xt-MMP N4 cDNA (Scaffold_119):** CAGCA**ATG**GAGTCCTGCGTTCTGTTCCTGCTGTGTATAGCATGTACCATGGGGTTCCCTGCTGGGATACTGACTGACACAGAGAAGCACAATGAAGCATTTGCTAAGGAGTACTTGAAAAAATTCTACAGGTTTGAGGGGGAGGGGACCAGCCGCAGTGCATTGTTGGATAAAATTCAAAAGATGCAGAACTTTTTTGGATTGGAGGTCACTGCAAGACTGAATGAAGAGACCATAGACGTAATGAAACAACCTCGGTGCGGGGTTCCTGATATTGCCCGTTTCAGCACCTTCTCAGGAAATGCAGTGTGGAAGAAAAAGGACCTGACGTACAGAATTCTGAATTACACCCCAGATATGACCAGAAATGATGTGGACCGGGCCATTGAGAAGGCTTTCAAGGTCTGGAGTGATGTCGTCCCTCTCACCTTTACCAGAATCTATGACAGAGTCTCTGATATTGAGATGCTGTTTGCATATAGAGATCATAAAGATTCTCTGCCTTTTGATGGCCCATCTGGCATTCTGGCCCATGCATTTGCTCCTGGAGATAACATTGGGGGGGACGTACATTTTGATGAAGACGAGAGATGGACAAGTGGATCAGCCGGTACTAACTTGTTCCTTGTGGCTGCTCATGAACTTGGCCATTCCCTTGGGCTCGACCATTCTAATGACCCTAATGCCCTTATGTACCCAACCTACCATTATATCAATCCCAACACATTCCAGCTTCCTCAGGATGATATCAATGGAATCCACTCACTGTATGGAACAAAAGAAAATCCAATCGTCCCAATAATAAACTGTCAGGAACAAATTTCTTTTGACGCGGTCACAACCCTGCGTGGGGACATTCTATTTTTCAAGAACAGATCCTTTTGGCGCAAGACAGCCGGTAAATCAGAAATCGAGCAACATGAGATCAGTTCCTTCTGGCCTTCCCTTCCAGCTGACATCGACGCAGCCTATGAAAATCAGAAAAAGGATCAGGTTCTTCTCTTTAAAGGAACAAAATACTGGGTTCTACGAGGCTATACAGTTCAAGAGGGATTCCCTAAGAACATCTACGAACTTGGGTTTCCCTTGACTGTAACGCAAATTGATGCAGCCGTCCACGATGATGAAACAGGGAGAACCTACTTCTTTATTAATGATCGGTATTGGAGTTATAATGAAGAAACATCACAGATGGATAAAGAATCTCCTCAGAGAATCAGTAAAGGTTTCCCAGGTGTTGGAAACAAGGTGGAGGCAGCATTTCGATCAAATGGAATGCTCTATTTGTTCAGTGGGAACCAGCAGTATGAATTCAGCACGACAAACAGGAAAGTCACTCGCCTGCTAAAGTACACCAGTTGGCTGAACTGCTAACCCAGAAACCCCAGCAGCGATAGGAGTTACCAAATGAACACAAGTTCAATAACAACCTTATTTATTTTTTTACATCTAACTTATTTTATAATTTATAGAAATATATATTGTATATTATCTTCTTGTATGTAACTTATTTAATTTATCTTTACTATACTATATTTTATATATCTTGATGTAAAACATATTTCATAATAAATATACTGATTTTTCAATGCAC

**26. Xt-MMP N5 cDNA (Scaffold_119):**

CAGCA**ATG**GAGTCCTGGGTTCTGTTCCTGCTGTGTATAGCATGTTGCACGGCATTCCCAGCCTGGATACAGACAGACACAGATAAGAATAATGAGGCATTTGCTAAGGAGTACTTGGGAAAATTCTACAAATCTAAGGACGATGGAAAAATGAGCAGAAGTGGGTTTTTGGAGAAAATCCGTCGGATGCAGGATGTTTTGGGACTGGAGGTCACTGCAAGAATGGATAAAGAAAACATAGAAGCAATGAAACAACCTCGGTGCGGGGTTCCTAATATTGGCCGTTTTAGTGCCTTCCCAAGAAATCCTGTGTGGAAGAAAAAGGACCTGACGTACAGAATTCTGAACTATACTTCACATATGACCAGGGATGAGGTGGACCGGGCCATTGAGAAGGCTTTCAAGGTCTGGAGTGATGTCGTCCCTCTCACCTTTACCAGAATCTATGACAGAGTCTCTGATATTGAGATGTCGTTTGCATCTGGAGATCACAAAGATGCTTTCCCTTTTGATGGCCCATCTGGCATTCTGGCCCATACATTTGCTCCTGGAGATAATACTGGGGGGGACGTACATTTTGATGCGGATGAAACATGGACAAGTGGATCAGCCGGTACTAACTTGTTCCTTGTGGCTGCTCATGAACTTGGCCATTCCCTTGGGCTCGACCATTCTAATGACCTTAGTGCCCTTATGTACCCAACCTACCATTATACCAATCCCAATACATTCCAGCTTTCTGAGGATGATATCAAGGGAATCCACTCACTGTATGGATCCTTTTGGCGCAAGACAGCCGGTAAATCAGAAATCGAGCAACATGAGATCAGTTCCTTCTGGCCTTCCCTTCCAGCTGACATCGACGCAGCCTATGAAAATCAGAAAAAGGATCAGGTTCTTCTCTTTAAAGGAACAAAATACTGGGTTCTACGAGGCTATACAGTTCAAGAGGGATTCCCTAAGAACATCTACGAACTTGGGTTTCCCTTGACTGTAACGCAAATTGATGCAGCCGTCCATGATGATGAAACAGGGAGAACCTACTTCTTTATTAATGATCGGTATTGGAGTTATAATGAAGAAACATCACAGATGGATAAAGAATCTCCTCAGAGAATCAGTAAAGGTTTCCCAGGTGTTGGAAACAAGGTGGAGGCAGCATTTCGATCAAATGGTGAGCTCTATTTGTTCAGTGGGAACCAGCAGTATGAATTCAGCACGACAAACAGGAAAGTCACTCGCCTGCTAAAGTACACCAGTTGGCTGAACTGCTAA

**27. Xt-MMP N6 cDNA (Scaffold_32):** CTCTGGTCTATTTGATCTCCCAGATTCCAGTATTTATGTACTAAGGGATTGATAAGTATTTTACAGTATCTTGCCATATTAACTGTCTCCCAAGCCTGCCCATTCCTTGAAGCACTTTAGGTCACCATCACTATATTATTAAGGCTATTTTCATCACTGTGTAGAAT**ATG**GATTGGGGATTGGCTGATAGAAGTTATGGCTGGATTTGTGTTTCTGCTGCTGTCACTTTCCTGTTACTCAGCAACTGGTTCTGTTACCAGCAGAAATCTTTGATTTTCCCAAAGTTTCAGAATGAAGTTGTGGAATGCGGCACAAACTGTTCCTTTAGAAGCAGCATACTAACTAGAAGAAAAAGATATACCATTAATCCTTTGGGGTACAAGTGGGACCATTTAAATCTGACCTACAAGATTGTTCAATTTCCCAACACCCTGAATAAAGATGATACAGAGAGAGCACTGGCCCTTGCCTTCCGCATGTGGAGTAAGGTTTCCTCCCTCACATTTCAGCGTGTACAATCACACCAGGTCTCTGACCTAAGAATAGGATTTTACACCTTTAATCACAGTGATTGTTGGGGGTCCCCACTCCACCCCTGCTTTGATGGCTTAAATGGGGAATTAGCTCATGCTTTTTTGCCACCACGAGGGGAAATTCATTTTGACAACCATGAGTTCTGGGTCCTTGGACCTTCACGCTTCAGTTGGAAACAAGGTGTGTGGTACAATGATCTTGTCCAAGTTGCGGCACATGAGATTGGTCATGCACTTGGGTTGTGGCATTCTAGTAACGTAACCGCTCTCATGCATCCCAACGCTACCTACACCAGGATACGTCATGTCACGAAAGATGATATCATGGCCATACAGAGCCTCTATGGATGTCCTTCTTCAGGCTCTAGATGTTATTCCCTTGAGCCTTCGGGGTCCTGTGGGAAGCAGTGTCATCTGAAATGTGACAGTTGCAAAGAGTCTCTTGACCAAAGACCAAAACAATATAGGATTAAAATAAAGAATCGTTATGTTTCCCAGGGTCGACCTGTTACCTTTCATTGCTCCCATAAAGTCTCCCAAGCATCCAAGAGGGTAAGTTGGTATAAAGATGGAGCTCGTCTCTCCTCCTCTACACCTGGGTTAGTTAACTTGTCCCTAAGCAGCCTGGTTCTAAAAGCAGAGGAAGAAACCCAGGGCCGCTACACATGTGTGATTCGCCATGGCAAAGTCATTGTAGGAGGAAAGTCTTGGAACCTCCACATCACATGAGCAGAGAAAAGAAAGATGACATGTCACTGCACTAGTCATGCAATAAAAACATGTCTGGGGTGCAAAATAAAAAAGCAATGACACTG

***X. laevis* MMP cDNA sequences:**

**28. Xl-MMP1A cDNA (BC054233):**

GAGAGCAAGCTTGGAAGCTTCAACTTTGAGGCAGTACACTCCAAGAAACAACTAACAAGCCAAAAACAAT

C**ATG**AAGTCCTGGATTCTGTTGCTGCTGTGTATAGCATGTTGCACAGCATTCCCTGCTAGGATACAGACA

GACATAGAAAAGAATGATGGGAAGGTTGCTGAGGAGTTCTTGAAGAAATACTTCAACCTTCAGACAGAAG

GAGGCCGTCTGGCAAGAAAAAAGGGCAGCAATGCATTTTCAGAGAAAATCCGCGAGATGCAAGACTTCTA

TGGTTTGGAGGTCACTGGAACACTGGACGAAGAAACCTTAGAAGTGATGCAGCAACCTCGATGTGGTATT

GCTGACGTGGCCAATTTCAGAGTTTTCCCTGGAAACCCCGTGTGGAACAAGAAAGACCTGACATACAGAA

TTCAGAACTATACCCCAGACATGACCAGAGATGAGGTGGACCGGGCCATTCAGAAGGCTTTCAAAGTCTG

GAGTGATGTCACCCCTCTTACTTTCACTAGGGTCAATGATGGCGTCGCTGATATTGAGATCTCATTTGCA

GCTCGAGATCATAAAGATTTTTATCCTTTTGATGGCCCATATGGTACCCTGGCACATGCATTTGCACCTG

GAAATAACAATGGAGGGGACGCACATTTTGATGAAGATGAAGACTGGACAAGTGGATCAGTTGGTTTCAA

CTTGTTCCTTGTGGCTGCTCATGAGTTTGGCCATTCCCTTGGGCTCTACCATTCTAATGACCCTAATGCC

CTGATGTACCCAAACTACCATTACGTCAATCCCAATACATACCAGTTGCCTCAGGATGACCTTAATGGAA

TCCAGTCACTGTACGGAGCAAGGACAAAGCCTGCTGAACCATCAACACCAAGCAACCCAACAAACTGCCC

CCCAAACATAACATTTGATGCAATTACAACACTGCGTGGGGAAATGTTGTTCTTTAAACACAGATCCTTC

TGGCGCAAGATCCCCAATAAATCAGAAATTGAGCATTATGAGATCAGGACATTCTGGAGTTCTCTTCCCA

GTGGAATTCAGGCAGCCTATGAAAATCAGGAAAAGGATCAAGTTTTTTTATTTAAAGGAACAAAATACTG

GGCTCTAAATGGCTTTGACATTGAGAAGGGATTCCCTAAGAGCATCTACCAACTTGGATTTCCCCAGTCT

GTGAAGAAAATCGATGCAGCTGTTCACGTCGAAGAAACAGGGAAAACATACTTCTTTGTTAAAAATCAGT

ATTGGAGTTATGATGAAGCAAAATCACGAATGGATAAGGACTCTCCTCACAGCATCATTAATGGTTTCCC

AGGCGTTGGAAACAAGGTGCAAGCAGTCTTCCAATCAAATGGAATGCTCTATTTCTTCAATGGCAATCGG

CAGTATGAATTCAGCATGGCAAAGAAGAGGGTCTTGCGTCTGTTAAAGTATACCAGTTGGTTGAACTGCT

AATCTGGAGACACCAGCAGTGACCGAAGGAACCAAATGAACAGCGTTCAATAAGCGCCTTGTTTTCTTTT

TCACATTTTACTTAAATTTTAATTTATATAAACATAAATATTTGTGTTATTCTGTATTATTATAACAATA

TGCTTTCTACTTTTTGCTATGTATGTTTTATATTGAGTATATTAAATTTATTCAATTTACAAAAAAAAAA

AAAAAAAAAAAAAAAAAAAA

**29. Xl-MMP1B cDNA (BC084836):**

GGGGAGAGCAAGGTTGGAAACTTCAACATTGAAGCGGTAGACTCCAAGAAACAGATAACCAGCCAAGAGC

AGCT**ATG**AAGTCCTGGATTCTTTTGCTGCTGTGTATAGCCTGTTGCACGGCATTCCCTGCTGGGACCCTG

ACAGACACAGAAAAGAATGATGGGAAGTTTGCTGAGGAGTTCTTGAAAAAATACTTCAGCCTTCAGACAG

ACGGGCTCCGCCTTGCAAAAAAGAAGGGCAGCAATGCATTTTCAGAGAAAATCCGTGAAATGCAAGACTT

CTATGGTTTGGAGGTCACTGGAACACTGGACGAAGAAACCTTAGATGTGATGCAACAACCTCGTTGTGGG

ATTTCTGACGTGGGCAATTTTAGAGCCTTCCCTGGAAACCCTGTGTGGAAGAAGAAAGACCTGACATACA

GAATTTTGAACTATACCCCAGATATGACCAGAGAGGAGGTGGATCGGGCCATTCAAAAGGCTTTCAAGGT

CTGGAGTGATGTTACCCCACTAACCTTCACAAGGGTCAATGATGGTGTCGCTGATATTGAGATCTCATTT

GCAGCTCAAGTTCATAATGATTTTTATCCTTTTGATGGCCCATATGGTACCCTGGCACATGCATTTGCCC

CTGGAAATAACATTGGAGGTGATGCACATTTTGACGAAGATGAAGACTGGACAAGTGGATCAGTTGGTTT

CAACTTGTTCCTTGTGGCTGCTCATGAGTTTGGTCATTCACTTGGGCTCTTCCATTCTAATGACCCTAGT

GCCCTTATGTACCCAACCTACCATTACGTCGATCCCAGTACATTCCAGCTTCCTCAGGATGACGTCAATG

GAATCCAGTCGCTATATGGAGCAAGGACAAAGCCTGCTGAACCATCAACACCAAGCAACCCAACAAACTG

CCCCCCAAACATAACATTTGATGCTATAACAACATTGCGTGGGGAAATTTTGTTCTTTAAACACAGATCC

TTCTGGCGTAAAATCCCCAATAATTCAGAAATTGAGCATCATGAGATCAGGACATTCTGGAGTTCTCTTC

CCAGTGGAATTCAGGCAGCCTATGAAAATCAGGAAAAGGACCAAGTTCTTCTCTTTAAAGGAACAAAATA

CTGGGCTCTTAAAGGCTATGACATTGAGGAGGGATTCCCAAAGAGCATCTACCAACTTGGATTTCCCCAG

ACTGTAAAGAAAATTGATGCAGCTGTACACGTTGAAGAAATGGGAAAAACATACTTCTTTGTTAATGATC

AGTATTGGAGTTATGATGAAGAAAAATTGCAAATGGATAAAGACTCTCCTCGGACCATCATTAATGGTTT

CCCAGGTGTTCCAAACAAGGTGCAAGCAGTCTTCCAGTCAAATGGAATGCTCTATTTCTTCAATGGCAAT

CGACAGTACGAATTCAGCATAACGAAGAAGAGAGTCTTGCGCCTGCTAAAGCACACCAGTTGGTTGAACT

GCTAATCCAGAGACACCAGCAGTGATGCAAGCAATCTAATGAACAGCGTTCAATAAGCACCTTATGCTCT

TTTTAACATTTAATTTAATTTTTAATTTATATAAACATAAATATTTGTGCTGTTTTGTATTATTATAATA

TGCATTCTACATTTTTTGGTATTTATGTTTTATATTAAGTCTATTAAATGTATTCATTTTTCGAAAAAAA

AAAAAAAAAAAAAAAAAAAAAA

**30. Xl-MMP2 cDNA (AY037943):**

GCACGAGGAAACTGCAACACAGACATTAACTCAAAGCTGTTTACCTGGAATGAGCAGTCCATTTATCTAT

TAGAACAGTTCCATAAAGGTTTTCGGCAGAAAGTCAGGCTTTAGC**ATG**CGGACAATTAAAATTATTAACG

TTTTAGTACTCATTTTCAATAGTTTTAGTATATTTTATTATGTTTCCCCTGCTCCATCTCCTATCATAAA

GTTTCCAGGAGACAAATCTCCAAAGACAGACGTGGAACTTGCTGCGCAATACCTGAATCAGTTTTATGGC

TGCCCTAAGGAAAAATGTCACCTGATGGTTCTTAAGGATGCCCTAAAGAAAATGCAAAGTTTTTTTGGTC

TTCCTGAAACTGGGGAGTTTGATCAAAATACCATTGAAACCATGAAAAAGCCAAGATGTGGGAACCCCGA

TGTAGCCAACTATAATTTCTTTCCCAGGAAACCGAAATGGGACAAAAACCATCTGACATACAGGATTCTT

GGTTATACAACAGATCTGGATTCTGAAACAGTTGATGATGCTTTTGCACGTGCTTTTAAAGTTTGGAGTG

ATGTCACACCATTGGAATTTAACAGAATTCATGATGGAGAAGCAGATATCATGATCAATTTTGGACGATG

GGAACATGGTGATGGATATCCATTTGATGGCAAAGATGGACTTCTGGCTCATGCATTTGCACCTGGATCA

GGAGTTGGAGGCGATTCCCATTTTGACGATGATGAGCTTTGGACACTAGGAGAAGGCCAAGTTGTGAAAG

TAAAGTATGGTAATGCAGATGGAGAGTTCTGCAACTTTCCTTTCCTTTTTAATGATAAGGAATACAACAG

CTGCACTGATTCTGGTCGCTCAGATGGCTTCCTTTGGTGCTCCACTACATATGATTTTGACAAGGATGGG

AAATATGGCTTTTGTCCACATGAGTTGCTATTTACCTTAACTGGTAACGCAGAAGGGAAGCCCTGCAAAT

TTCCTTTTAAGTTTCAAGGCAACACATACAACAGCTGCACCACTGAAGGGCGAACAGATGGCTACAGGTG

GTGTGCTACCACAGAAGACTATGACAAGGATAAGATGTACGGCTTCTGTCCGGAAACAGCATTGTCCACT

GTTGGAGGAAATGCTGAAGGTTCCCCATGTGTCTTTCCTTTCACCTTCCTGGGAAACAAGTATGATTCGT

GCACCAGCTCTGGTCGCAGTGATGGAAAACTATGGTGTGCATCAAGCAGTAACTATGATGATGATCGCAA

GTGGGGATTCTGTCCTGATCAAGGTTACAGTCTTTTCCTAGTAGCTGCTCATGAATTTGGTCATGCTTTA

GGACTAGAGCACTCTCAGGATCCTGGAGCATTAATGGCTCCAATTTATACATACATAAAGAACTTTCGGC

TTTCACAAGATGATATTACTGGGATTCAAGAACTCTATGGTCAGGGATCCAAGGAAAAACCAGGTCCTGG

ACCTATCCCAACAATGGGCCCTGTTACCCCTGATCTGTGCTCTAAAGATGTTGTACTTGATGGCATGTCT

CAAATAAGAGGGGAAACATTCTTTTTTAAAGACAGATTTATATGGCGCACTCCAAACATAAGAAATAAAC

CATCAGGACCTCTACTTATTGCTACCTTCTGGCCTGAGCTACCTGATAAGATTGATGCTGTTTATGAAGA

ACCTCAAGAAGAGAAGACTGTTTTTTTTGCAGGTAATGAATATTGGGTCTACTCATCAAGTACTTTGGAA

AGAGGTTACCCAAAAAAGTTGACCAGTTTAGGACTACCTCCTGATGTTGATCGTGTTGATGCTGCGTTCA

ACTGGAGCAAAAACAAAAAGACATACTTCTTTGCTGGAGACAAGTTCTGGAGATATAATGAAGTAAAAAA

GAAAATGGACACTGGTTTCCCAAAACTAATTGCAGATGCATGGAATGGTGTACCAGATAATTTAGATGCT

GTTCTAGATCAAACCGGAAGTGGCTACAGCTACTTTTTCAAAGACTGGTATTACTTTCAAGTTGAAGGCA

AAAGTGTGAAGATTGTAAAGGTTGGCAATGTCAAAAATGACTGGCTACGCTGCTGAAATATATTCATTTA

ATTACTCTGTATTGCTTAACATGAACTTTCATACGTGTATGGCATTTTTATAGCATAATAAACTATTATA

AGGGACCAGTCTGGTACTGGTATTTGGGGAGCATACCAAGATATACTACAGTAACACTCAAACACTGCAT

TTTTAAGAATTTGTTCATTCCATTAGTTTTTTTCACTTGCTTGCTTAACTATAGCTTTTAACTTCATCTT

AAAATGTTTTTATTATTGTAAGCAGTGTGACAATGAGTTAACTCCTGCTACTTTTTTATATTGACCTTAA

TAAATCAAATCAAATTTACTAACCCATTTGTACATAGGTACCGGCTGCCTATACTGTACACTTTAATAAT

GCTATGGATTTTTGAGAGAAAACACTGATAAGACCCCATTTCTGTAAGTGCTTTCAATTTTATTCACCAA

ACTGCAGTGAGAGACTACAGTGCAAAATTAATCAACTATTGTGTGCTGTTATTTTCACACAGCTGTATGT

TATCCATATTTCAAAAGAAAATCTGTACATCATCAAAAGAACGTATATAATTATAACATAAAAGATAAAC

ATTCAAAGTGTCTAATTTGTGCGCAAAAAGTAAAATAAATGGGCAATTGTAGTTATGCATTGACATTACT

TTTTGGAATGCTTTTAAGGGTATTTAAAGTGTACCTGTCACCCAGACACAAAAAGCTGTATAATAAAAGT

CCTTTTCAAATTAAAAAAAAAAAAAAAAA

**31. Xl-MMP3 cDNA (BC077966):**

GGAAAAGAGAAAGGAAGAAGCTGAGGGGAGCGCAAGATGTTTCTCTCCTGGCTCCTCGTTCAGAGTGTCC

TGCTGTACATTTATATGGTGGAGTTGGTGCCACTTCCAGAAGAACCCACATACCTGACCCATGGGGAAGT

ACAAGCAGCTCCAGAGATTTCTGAATTTACC**ATG**GAGATGATAGAAATTACAGAAAATGACCAGCTAAAA

GTTCAGGAATATCTAAATCAGTACTACAGCGGCGTTACAGCAATTGGAAGAAAGGCATCGCCAACAGAAG

AAAAGATAAAGGCCATGCAAAGATTCCTGGGATTGGAAGTCACTGGGAAAATTGATGTCAATACCATGAA

AGTTATACAGAAACCCAGATGCGGGGTCCCGGACGTTCAAAAATTCAGTCATTTCGCTGGAAATCCAAAA

TGGCAAAAAACAACTATAACCTACAGAATTCTTAACTACACTCCGGATATCACTAAATCAGAAGTAGACT

ATGCCATTGCACAAGCTTTTAGAGTGTGGAGCGATGTTACCCCTTTAAATTTCCAAAAGCTGAATAGCGT

AGATGCCGATATACTGATCTCTTTTAACACTAAAGCTCATGGTGATTTTAATTCGTTTGATGGGCCCAAC

GGTGTTCTGGCACATGCTTATGCACCCGCCGAGGGCATTGGGGGAGATGCACATTTTGATGAGGATGAAC

AATGGACGTTGGGACCTCAAGGTGCTAATATTTTTCTTGTTGCTGCCCATGAGTTTGGCCATTCCCTTGG

CCTGTCTCATTCCAACGACCCCAATGCTTTGATGTTCCCAACGGCCTCGTTTGGCATGACGATTAACCCT

GCTCAGTATAAGCTTTCTGCAGATGATATCGCAGGCATCCAGACTTTATATGGAAGAAGAAACTCCATTG

AACCAAAGCCCAATCCAATGCCACCAGTGATCACCCCCAAACCAGCACCGCCCCCAAAGAACCAGCCAAA

TAAGTGTGATCCCAACCTTCAGTTTGATGCTGTTACGAGCATGAGAGGAGATCTGCTCTTTTTCAAAGAC

GGGGTGTTCTGGAGAAAGAGCGCACGATTTCCTGAAGTGGAAACCATCTCCATGAATATTATTTGGCCGA

GCGTGGGAAGAGTGGATGCAGCTTATGAAGTTGTAGGGAGAGACATAGTATATCTATTCAAAGGACGACA

ACACTGGGCCACAAGAGGATGGACCATTCTTCCGGGATATCCAAAGGACATCAGCTCATTCGGATTTCCG

TATGATGTTAAAAAAATAGATGCCGCGACCTTCATCAGAGAAGAAATGAAAGCCATTTTCTTTGTTGGAG

ATAGATATTATAGCTACAACCACAGAACCAACGCAATGGATTCCAGGAAACCTCGAAAAATAAAATCTGA

TTTTCCTGGAATTGGAAAAAAAGTTGATGCGGCATTTCAGAATGGTTATCTCTACTTCTCTGCAGGAGCC

AAACAAGCGGAATATGATTACAGAGGGAAAAAGGTGGTCCGTTACCTGCAGAACTACAGATGGATGAGCT

GCAAATGAAACTGATTAGGTCTTACTATAAAAAAACCACACCCTTTATGTTTGGGGTGGTTTCTTAGAAA

AAGTTATATTATTATAATAATATAACTTTTTCTAAGAAATTACTTTAAACATAACCATTCCTATTATAGA

GCTAAGACTTACTGCACATCGCTACTACAGGTATTAGACCGGTTATCCAGAATACTCTGGACCTGGGGCT

TTCCAGATAACGGATCTTTCCGTAATTTGGATCTCGATACCTTAAGTCTACTAGAAAATCATGTAAATAT

TAAAAAAACTAATAGGCTGGTTTTGCTTCCAATGACGGTTAATTATATCTTAGTTTGGATCAAGTTCAAG

CTACTGTTTTATTACTACATTGAAAAAGGAAATCATTATAAAAAATTTGGATTTGGATAAAATTGAGTCT

ATGGGAGAGGGGCATCCCATGATTCAGAGCTTTCTGGATAATGGGTTTCTAGATGACGGATTCCATACCT

GTATAGCACTTGGTAGAAGTGAGATGTGCATATCATAGATATATGCATACAAAAGCACAGTGTTAGAATG

GTTACAATTAGTTTTTTTGCACAGATTAAGCCACAAATAATTTTAAAAACATAGTAATATCTGGTGCAAA

TCTCTGAAGCAATCCTTTTACCAGTTGATTGTGTTTTACAGATGCATGCACATACACATTATTCACGCTC

TTCTTAAAGACGTTTCGGGCCTAAAAAAACAGAAGTGTTATTAATGATTTGTTATTATCTGGCTCCTTTG

ATCTGTATAGTGACAATCTCGGCATTTGCACAATTATTAAAGGACAGTTATGCCAATCACATCCACAGTA

ATAACAATGTATGGCTTCCACTGTTGAATAAATCTTTGTGTCTGAAAAAAAAAAAAAAAA

**32. Xl-MMP7A cDNA (AY573380):**

CCACGCGTCCGGACTGTCTGATTTCCAAGTGTCACC**ATG**CTCCAAGAATTCCTGTTAGTTTTCTTGGCAC

TGTCCTGTATCCAGGCCATGCCAATGCCCCAACCTGAAGATCCTATGAGCCCTTCGGAACGTATGTTTGC

AGAGAAATACTTAGACACTTTCTATCAAATGAGGTCAAAGAACACTTTCGCTGAAAAACTCAAAGAGATG

CAAAAGTTCTTTGGAATGTCGGTGACAGGGAGGTTGGACTCGGACACCATGAAGATGATGAAGACCCCTC

GGTGCGGAATGCCCGATGTTGCAGAATTCCGACAGTTCCCTGGGAATCCCAGATGGTCAAAAGCGCAGCT

GACTTACAGCATTGTGAATTACACTCCCGACCTGCGTCGCCAAGTGGTAGATGACGCCATCCAGAGGGCA

TTTGGGGTGTGGAGTAACGTGACCCCGCTGCAATTCACAAAAGTTTCCTCTGGAAATGCAGATATATTCA

TCCGATTCGGAGCACGCACGCATGGAGATTCCAACCCATTTGATGGCCCCAGTGGAGTTTTAGCTCACGC

CTATGGTCCAGGGCGCGGCATTGGAGGTGATGCCCATTTTGATGAGGATGAAAGATGGACAAGTTCTAGA

GCAGGTTTTAACTTGTTTCTAGTGGCTGCGCATGAATTTGGTCATTCTCTGGGACTTGATCATTCTACTG

ATCCACAAGCGTTGATGTTCCCAACCTACCATTATGTGGAAACCACATCCTTCCGTTTGTCACAAGATGA

CATTAACGGAATACAGTCAATCTAATGGGGAGAAGGCAAAAGGCTTAGCAACACGGCCTTGATACCAGTA

AAGTGCAAATTTACTTGCTTTCAGCTTGGCCATCATTTCACTGCTATATAGAAGAACTATAGTGAATTCC

AAAATAATTCTCTCTCTCTCTCTCTCTCTCTCTCTCTCTCTCTCTCTCTTTCTCTCTCTCTGTTAGTTAT

TTTGGGTGGCCCTACTCTACTATAGAGTCTGCCTGGTTCCTTGCTGCCTACCATAGTTGCATCTGTAGGA

TTCATTAACTAAGGCATCGCCATTCCATTTCAGAACCACAAATTGTCCTTTTGTTTATAAACTATAGGAG

CCAATAAAGTACTTTTTGAAGCAATAATTTAACATCTGGTGTGCTACAGTGAACATTGAATTTTTCTATT

TGCTATAACCCGTGGCACACAGTTACAGCACCTGCAAGGGTCATTTGATCAGATTGGGCACTCCATAGTG

TAGAAGCCGCTGGCCCAGTCACTCCCAGTTATGCCCAGACCCATGCAGACTGCAGACCGGGTGCAAGAGC

TGTGTATTGTTCCACGCATTAGTAATTGAGTTTTAAAGGAATTGTTCAGTGTAAAAATAAATATGGGTGA

ATAGATAGGCTGTGCAAAATAAAAAATGTTTCTAATATAGTAAGTTAGCCAAAAATGTCATCTATAAAGG

CTGGAGTGACTGGATGTATTCCATAGAAAAAAAAGTCCAATGGCACACAGGATTGATACAAGCAAGATTG

CCTTGTGTGTTTAATGCGGAGTGCAACATTTCTGGGTCACGCCCCTTTGTCAAGCATACGGAACAAGTGT

GCAGTGAGTATATATAAAGATTAACCAACAAGCAATTAAAGCATAACCATCATTCAATTAAGTCAATTAA

AGTGGACCTGTCACCCAGACACAAAAATCTGTATAATAAAAGTCCTTTTCAAATTAAAAAAAAAAAAAAA

GGG

**33. Xl-MMP7B cDNA (BC056040):**

GTCTGATTTCCAAGTGCCACC**ATG**CTCCAAGCAATCCCGTTAGTTATCTTGTCCCTGTCCTATATCAAAG

CTATGCCAGTGCCTCAACCTGAAGAACCAATGAGACCTTCGGAGCGTATGTTTGCAGAGAAATATTTAGA

CACATTCTATCCAATGGGATCAAAGAACACGTTTGCTGAAAAACTCAGAGAGATGCAGAAGTTCTTTGGG

ATGTCAGTGACGGGGAGGTTGGACTCGCATACGATGACAATGATGAAGACTCCTCGATGTGGAATGCCCG

ATGTTGCAGCATTCACACAGTTCTCTGGGAATCCCAGATGGTCAACAACCCAGCTGACATACAGCATTGT

GAATTACACTCCTGATCTGCCTCGCCAATTGGTAGATGAGGCCATAAAGAGGGCATTTGGGGTGTGGAGT

AACGTAACCCCACTGCAATTCACTGCAATTTCCTCTGGAGACGCAGATATATTCATCCGATTTGGAGCAC

GCGCGCATGGAGATTCCTTGCCATTCGATGGCCCCAGTGGAGTTTTAGCTCATGCCTATGCTCCAGGGCG

TGGAATTGGAGGTGATGCCCATTTTGATGAAGATGAAAGCTGGACAAGTTCTAGAGCAGGTTTTAACTTG

TTTCTAGTGGCTGCACACGAATTCGGACATTCTCTGGGACTTGATCATTCCACTGTTCCGCAAGCCTTGA

TGTTCCCAAACTACCGATATGAGGACACCGCAACCTTTAGTTTGTCACAAGATGACATCAATGGAATACA

GTCCATCTATGGCAAAAGGCAACAAGCTTAGCACCATCTTTTATACCACCTATAATCAAGGTGCAATTTG

GCATGCTTTTAGCTTGGCCGTCATTTCACTGCTATTTAGGACTATTGTGAATTCCACAATAATTCTCTCT

CTGTTAGTTATTATGGGTGGCCCTACTCTTGTAAAGAGTCTGCCTCATTCCTTGCTTCCTACCTACAAAA

AAAAGGGGGGATTAATTAATGCACATTCCATTGTCCCCCTTTCATGAGTAATCTAGTATCTATGCAAGTG

CAAGCATTTAAAAAAAAAAAAAAA

**34. Xl-MMP9 cDNA (AF072455):**

ACCCACGCGTCCGCCCACGCGTCCGTAAAGTCACGTATAGTGAAATTTAGAGTTTGTCTTGGTGCCAAC**A**

**TG**GGTAGGGTGGGAGTTTTAGTCTTAGTGACCATCCTTTGTTCCAAGGGTCACTCCGTTCCTATCGCCAG

CAAGTCCCCTCTAACCATTTTATTTCCAGGGGACATCCAGAGTGGCACAACTGATATGGAACTGGCTGAG

AGCTACCTCCTACAGTTTGGGTATCTTATCCAAGAACAGGGCTCAAATGCCACTTTACAAAATGCGCTCA

CCATTATGCAACAGAAACTGGGACTGAAGGAGACTGGAGTCCTAGATGCTGAAACACTGGAGGCCATGAA

AAGACCTCGCTGTGGGGTTCCAGATATTGGACAATTCAACACATTTGAGGGGGATTTAAAGTGGGATCAC

AATGATATCACTTACCGTATTCTGAACTACTCCCCTGACCTGGACCCTGAAGTGATCGATGATGCCTTCG

CCCGCGCATTCAAAGTCTGGAGTGATGTCACACCTCTGTCCTTCACTCGTATATACAGCGGGGAACCTGA

TATTAACATTCTGTTTGGGACTGAGGACCATGGGGATCCTTACCCCTTTGATAGGAAGGATGGTCTCTTG

GCTCATGCTTACCCTCCTGGCCCTGGGGTGCAGGGTGATGCTCACTTTGATGATGATGAATTTTGGACAC

TGGGTACTGGAACTGTGGTAAAGACTCGCTTTGGGAATGCTGAAGGGGCTCTGTGTCGCTTCCCATTCAC

ATTTGATGGACAGTCGTTCTCCACCTGTACAACTGCTGGACGCTCAGATGGGTTGCCTTGGTGCAGCACC

ACTCCCAATTATGATCAGGATAAAAAATACGGCTTCTGCCCCAGCGAGATGCTCTACACATATGGAGGCA

ACAGTAATGGGCAGCCTTGTGTTTTACCATTTATCTTCGACGGAGTGTCATATAATGGTTGTACCAAGGA

AGGCCGCCAAGATGGGTATCGCTGGTGCAGCACTACTGCCAACTTTGACCAAGACAAGAAATACGGATTT

TGCCCTAACCGAGACACATCTGTGATTGGTGGAAACTCCCAAGGAGAGCCTTGTGTCTTTCCATTCACAT

TCCTGGGGAAGATACACAATTCCTGCACTACAGATGGCCGGGATGACAGAAAGTTGTGGTGTGCCACCAC

CTCCAACTATGACCTAGACAGCAAGTGGGGATTCTGCCCTGATCAAGGGTACAGTCTTTTCCTGGTGGCA

GCCCATGAGTTTGGCCATGCACTTGGTCTGGATCACAGTGATGTGCAAGATGCTCTCATGTACCCCATGT

ACAGCTATGTGAAAGATTTCCAGCTTCATCAAGATGACGTGCGCGGGATCCAATTTCTATATGGTACAGG

TTCAGGCGCTCCTCCTAACCCCAATCCACCAAAGCCCACCAAGAAGCCATTAGCTACCAAACGTCCCAGA

ACCACCACAAGACCTGTACCTCCAGTGAACCCGGCACAAGATGCCTGTAATGTGGAGATGTTTGATGCCA

TTGCAGACCTACAGGGAGCTCTTCACTTCTTCAAAGATGGGTTGTATTGGACTCTGACTCCCAGAAGTAA

GAATTCTCCTCAGTCTCCTCTGCGCATCTCAGATACATGGCCAGCCCTCCCCAGCAAAATTGATACTGCA

TTCCAGGACCCCACCAGCAAAAGCATTTTTTTCTTCTCAGGGAGCAAGTTCTGGCAGTACACAGGTACAA

GTGCTATAGGCCCACGCAGCATCGAGAAGTTGGGTCTGAGTAAGGATGTGGAGGCTATCATGGGATCTTT

TGCTCGTGATAATGGAAAGGCCCTGTTATTCAATGGAGAACAATACTGGAGGCTTAATCTAAAGACATTG

ACTATTGATAATGGCTACCCACGGCAGACAGCTGACAACTATCCTGGTGTTCCGGGTGACTCCCATGATG

TTTTTCTCTATCAAGGAAACTATTACTTCTGCCAGGATCAATACTTCTGGCGTGTGACATCACGAAAGCA

ACAAGATATGGTTGGATATGTCAGCTATGACCTCCTTCGTTGCCCACAGAATTAAATGGGGAGCTAGGAG

AAATGGGGCTGCCGGATACTATTGGGGACACTGAGATGATTGATTTTGATTGGGGAAAGGAGTAGTGCAT

TTCAGATTTTGATTCAAGCCCTCCTTTGCAGTGAAAAATGAGGTCAATATTACAGAAAGCAATGCCAGTC

ATTCTGGTATAGTTTAGATTATCTATAGGGTGGCAAATAAATATATGCATTCCCTCTTTTGCATTCAGAC

TGCCCCATGTCTATTGCTTTGAGGCCTCCTAGGGACACACCATAGGTTGGGATCTTCTCCCATACATTGG

CATGAAAACAAAAGGAATCTACAGATATTGTGCCTATGAATTCAACAAATAGGATGTAATTGTTTTACTA

ACTGAGTTTATTTTGTAGAAATAAGGTATTGCCAGATGAACTTATGTATCCTGTGATATTATATATGTCT

GCTGTCTTTGCATGGTTTGAAAGGCCTTTACCCAATAGCAGTTTTCTTTTCCCACAATGTTAAACCATTA

TTGTGATGCATCTAGAAGTGTCCAAGAGAGGGTGAATATTACACTTTGCAGGGATGGCTGCCATTCTTAG

AGGGGTGGTTCACCTTTAAAGGAATTGTTCAGTATAAAAATAAAAACTAGGTTAATAGATAGACTGTGCA

AAATAAAACATGTTTCTAATATAGTTAGGCAAAAATGTTTTTATTTTCATACTGAACTGTTCCTTTAAGT

TAAATTTTAGTATGTTATAGAATGGCCAGTTCTAAGCATCTTTTCAATTGATCTTCGTTATTTATTTATT

TTTTCCAGCTTTCAAATAGGGGTCATCTATACAAATGCTCTTTAAAGCTACAAAGTATAGTTAATGCTAC

TTTTAATTACTCATCTTTGTATTCAGGCCCCTCCTTTTCATATTCCAGTCTTGTATTCAAATCACTGCAT

AATTGCTAGGGTAAGTTGGACCCTAGCAACAAGATTGCTAAAACTGTAGAGTTGCTGACGAAAAAGGCTA

AATAACTCAAAGACCACAAATAGTAAAAAATGAAAACCAATTGCAAGTTGTCTTAGAATATCACTCTCTG

CATCATACTAAAAGTTAAATCAAAGATGAACAGCCCCTTTAAAAGAACATCTGGCCTAAGCTGCTCAAGT

GAAGTAGCCTGTAGCAACCATTGGGAAAGGAGAAAATGAAAGCATAGGTCTGCTGAAGGCAACTGCAGTG

TGAAATTTTCATTAATGCTTTTCTAGGGTGGAATGTGTCTGTGGAATGTGTGTGTCTGATTTGCGCGTCA

GGTGTATATTCTGAATTACATGTAAATGGAACAAGATGTGACCTGTACTAATATCACTTTATAATATAAA

TAAAGCATTTTCTGCTCAGCC

**35. Xl-MMP9TH cDNA (AB288054):**

AGACAGGAGGCACCTATAGGGAGACAAGAAGAGTAAAAGGCAGAGCATTCCTAGGTGTCATT**ATG**GGTGG

GCTGTGTGTTTTTGTCCTAGTTTCTATACTTTGTGCGTGGGGTCACTCTGCTCCCACAGCCAGCAAGACC

CCTGTGTCAATTACATTTCCAGGGGAAATCCGTAAAAACATGACTAGTGTGGAGGTGGCGGAGTGGTATC

TGGTGAAGTTTGGGTATCTGCCCCTCCAGCAGGGCTCAAACCATCATGTCTCCTTAAAAAAAGCTCTCAG

CCAAATGCAAAGTAAGCTGGGACTAAAGGTTACTGGAAACCTGGATGCTGAAACATTGGATGCCATGAAA

ACCCCTCGATGTGCAGTACCAGATATTGGCAATTACAACACGTTTGAGGGGGAATTAAAGTGGGATCACA

ATGATCTCACATATCGTATCCTAAACTACTCCCCTGACCTGGACCCCGATGTGATCGATGATGCCTTCGC

CCGCGCATTCAAAGTCTGGAGTGATGTCACACCTCTGACCTTCACTCGTATATACAGTGGGGAACCCGAT

ATCAACATTATGTTTGGAACTGAGAACCATGGGGATCCTTACCCCTTCGATGGAAAGGATGGCCTCTTGG

CTCATGCATATCCACCAGGCCCTGGAATGCAAGGCGATGCTCACTTTGATGATGATGAGTTCTGGACACT

TGGTACTGGAATTGTGGTAAAGACTCGATTTGGGAATGCTGAAGGGGCCGCATGTCACTTTCCATTTGTA

TTCGATGGCCAGTCTTACAACTCCTGTACAAGTGATGGACGCTCAGATGGGCTGCCTTGGTGTAGCACCA

CCCCAGACTTTGATCAGGATAAAAAATATGGATTCTGCCCCAGCGAGTTGCTCTACACGTATGGAGGCAA

CAGCAACGGTGAGCCTTGTGTCTTTCCTTTCATCTTTGATGGAGTGTCGTATAAGGGCTGTACCAAGGAT

GGGCGTCAAGATGGATACCGATGGTGCAGCACCACTGCCAACTATGACCAAGACCATAAATATGGATTTT

GCCCTAACCGAGATACATCTGTGATTGGCGGAAACTCCCAGGGAGATCCTTGCGTCTTTCCATTCACATT

CCTGGGGAAGAGATACAATTCCTGCACTAGTGAGGGGCGCGGTGACAGGAAGTTGTGGTGTGCTACAACT

TCCAGCTATGACCAAGACAAGAAGTGGGGCTTCTGTCCTGATCAAGGGTACAGTCTTTTCCTGGTGGCAG

CTCATGAGTTTGGCCATTCGCTTGGTCTGGAGCACAGTGATGTGAAAGATGCTCTTATGTACCCCATGTA

CAGCTATGTGAAAGATTTCGAGCTTCATGAAGACGATGTGAATGGGATCCAATATTTATATGGGTCTGGG

CCACATCCCGCTCCACCAAAACCCACCGACAAGCCAATACCAACCACCACCCCATCCACCAGCATTACTA

CTACCACCCCATCCACCAGGACCACTACTACCACCCCACTGACACCATCCGTGAACCCTGCCCTAGATGC

TTGTAAAGTGAAAATGTTTGACGCCATTGCAGAGCTACAAGGAGCTCTGCACTTTTTCAAAGACGGGTTG

TTCTGGACTGTGACCTCCAAAAATAAGAATGCGCCACAGTCTCCTCGCAAAATCTCGGACACATGGCCAG

CCCTTCCCTCTAAAATTGACACTGCATTCCAGGACCCCACCAGCAAGAACATGTTTTTCTTCTCAGGGCG

CAAGTTCTGGCAGTATACAGGGAAGAGTGTCTTAGGTCCACGCAGCATTGAGAAGTTGGGTCTGAGCAAG

GATGTGGAGGGCATCATGGGATCTTTTGCTCGTGATAATGGAAAGGCCCTGTTATTCAACGGGGAGCGAT

ACTGGAGGCTTAATGTAAAGACATTGACCATCGACAAAGGATACCCACGACTGACAGATGTAGACTATGC

TGGTGTTCCAAGTGACTCCCATGATGTTTTCCTGTACCAAGGGAAATATTACTTCTGTCAGGATCGCTTC

TTCTGGCGCATGACATCACGCAAGCAAGTTGACAGAGTTGGTTATGTCAAATATGATCTCTTGCACTGCC

CAGAGCAAAAATAGATAGCTGAGGAAGAGTGGGTTGGTGTGTCT

**36. Xl-MMP11 cDNA (Z27093):**

ATCCACAGCTGGATTCTCATGCACACTGACAAGCACCGGACAGCTCCTCCGCGCGCACACACACAGAGCA

AGCAGGGAGAGAGCCAGAGCCACAGACAAAGACACCTACACAGACTCACAGGGAAAAGACTTATGTAAAG

GACTTGTGTAATAACTTGCACTTGGGAAACACTTGTAGCCATTGTATCACACTCACCCTACAGCTTGTGA

GA**ATG**CATCTCCTCATCCTTCTACCTGCACTGTGTGTACTTGGAGCTCACTCAGCCCCTCTGTCATACAC

TTACCTTCAGCACAGAATCCAGGAAAAGCCTCAGAAAGATCATGGCAGGTTGCAGTTTAATTCATTACAT

TATCCTCATATAAAGGGACTGCTCAATGCTCACGGTTCATGGAACCCCCCACGCTGTGGAGTACCAGATA

TTCCTGCCCCTCCAGATTCCTCCAGTGGTCGAAATCGTCAGAAACGGTTTGTCCTGTCGGGAGGACGCTG

GGACAAGACAAACCTGACGTACAAGATCATCCGTTTCCCATGGCAGCTAAGTAAGGTGAAAGTGAGACGC

ACTATTGCAGAAGCCCTAAAGGTATGGAGCGAGGTTACCCCTTTGACTTTCACTGAGGTGCATGAAGGAC

GCTCTGATATCATAATTGACTTCACACGGTACTGGCATGGAGATAATCTCCCATTTGATGGTCCGGGGGG

TATCTTGGCACATGCTTTCTTCCCAAAAACCCATCGAGAGGGGGATGTACATTTTGATTATGATGAAGCT

TGGACTATTGGAAACAATATAGGTACAGACCTACTTCAAGTAGCTGCCCATGAGTTTGGTCATATGCTCG

GCCTGCAGCATTCTTCTATCTCCAAATCGCTTATGTCACCATTTTACACATTCCGTTACCCACTAAGCCT

TAGTGCAGATGACAAGCATGGCATACAATTTTTGTATGGGGCTCCGCGTCCTCCGACTCCTTCCCCAACC

CCTAGGGTGGAGGTCAACCAGGTGGAAAATGAGAGTAATGAGATTCCTGCGGCAGAGCCTGATGCATGCA

AAACTAATTTTGATGCGGTGTCTACAATTCGTGGAGAGTTATTCTTCTTCAAGTCTGGTTATGTATGGCG

CCTTCGTGGTGGGAAATTGCAGAATGGCTACCCAGCCCTGGCATCACGCCACTGGCGAGGGATCCCTGAT

ACAGTCGATGCAGCATTTGAAGATTCTGTGGGAAACATCTGGTTCTTCTATGGCTCACAGTTTTGGGTCT

TTGATGGGAAGCTGCAGGCGTCGGGGCCCTTTCCAATTACTGACATTGGCATATCAGTGACACAGATTCA

GGCCGCCTTCGTGTGGGGTACAGAAAAGAACAAGAAAACCTATCTGTTTAGGGGTGGAGAATACTGGCGA

TTCAACCCAGAAACTAGACGAGTGGAAAGCCGGCACTCACGGAGGATTGGAGACTGGAGAGGGGTACCTA

AAGGCATTGATGCTGCTTTCCAGGATGAACAAGGTTATGCCTATTTTGTAAAAGGACGACAGTACTGGAA

ATTTGACCCATTCAAAGTTCGTGTCATGGATGGGTATCCTCACCTGATCAGTCAAGATTTCTTCAATTGT

CAAGCAAGCTCTACATTTGTTAATTCATTAAGATGAGCCGGCTCATGTTTCTTACCTTGGATTGCAGGGA

GTACTCTGTAATACAGACTGGCCAGGCAGGACCACAGGGCCCATATCTGACTCACTGATTTGCAATTCAG

AATGCAATGGGATCAGTAGAACTGCAATATGGTCTCACATACACCATTTGGAATGTGAACCCTCAAAAAT

CCAGCTACTTGGTATGGGAAATACAAACAAAATAAAAAACATATTTTACCTTATATTGTAAAACAGCATA

TCTGTTTTGAATTTCTCCAGGCATGTTACCTAGGATGGAAATCAGCATCCCCAAACACTTTGCAGAGCTC

TAAGACTGTGTTGTGTGTTTCGGACACTGGGATCTTCACACTTTCTTTGTTGCACAAGCCATTTGAAGTT

AGCCGTGCAGTTTGCTTTAATACCAATGCTATGTTCTCACAATACCTCTGTCAGTGCTGGACACTGATCC

TGATATGAAACTCTTCAAGTGTGGAAGTATTTGGAGATTTTTTTTTTTGTGACTTTTTTTTTATCATTTA

CTGATAAATATGACTGCTCTATTTGCTCATAGATAGTGCTAAACTCTGGATTGTCCAACAAAATTGTCCA

CAATTATCCAAAAATAACAAGCCACATTAGAAAAAAAAAAATTCTATGTGAAATCTCGAAAAATCTAAAA

TAGAAGAAGAAAAGGTTAAATATGAAAGGGAATAGAATTTTGGTAAATCTGAAACCTAAAAATCTGAATG

AAATATTTAACAACTCTTGTGCATGGGGTTGAGAAAGACAACAAGCTTGTGTTTACCCACACGTTTGAAG

TTGACATGTCTGCTGTTATAAGCAAACAGAGATTGAGCAGGGAAATTCACCTAGGGGTAAGGATTACTGA

CTTTCTTGTAGGTTCATTGCAATTTGTCATTTAGCAAATAATCCCTAAAACAGCATATTCCAATAATGTT

CCTTATAAACCAATTGACTACAAAGTCATATTGGGATGGCTGAATACTAGATAAACCAAATTAGTGAAAC

CAGCCAGCAGCAATTAAAAGTTTAATAAACAATAAAAGCTTTTATGCCTTATTTTATAGGGGTCAGTTTA

CCACTCCTTTGACAGTCACTCTATTCGGTTCAGCACCATGTAGCCAACTACACAGTTGCATCCAATGTGT

GTTTTAATTACTAATGTGAATGTTATTTTGTACATAAATACATTTTTGTATGTTTTATATATGTAATAAA

TTTGCTTTGTTTTATAAAAA

**37. Xl-MMP13 cDNA (L49412):**

TCATCCTTGTCAGTGCTTGTCCTATCTCTGAGCTTCGCTTATTGCCTCTCAGCCCCTGTTCCACAGGATG

AAGACTCTGAACTGACACCAGGAGACCTACAGTTGGCTGAGCATTACCTAAATCGGCTTTACAGTTCCTC

ATCTAACCCTGTGGGTATGCTGAGGATGAAGAATGTGAATAGCATAGAGACCAAGCTGAAGGAGATGCAG

TCCTTCTTTGGTTTGGAGGTGACTGGGAAACTCAATGAAGATACCCTGGACATCATGAAACAGCCAAGAT

GTGGAGTCCCTGATGTTGGGCAATACAACTTCTTCCCAAGAAAACTGAAATGGCCAAGAAATAACTTGAC

ATACAGGATTGTGAACTACACCCCAGATTTATCCACCAGTGAAGTCGATAGGGCCATCAAAAAAGCACTG

AAGGTATGGAGTGATGTAACGCCACTGAACTTCACTAGGCTCCGCACTGGCACCGCTGACATCATGGTCT

CTTTTGGCAAGAAAGAACATGGAGACTACTATCCATTTGATGGACCAGATGGTTTGCTGGCTCATGCCTT

TCCACCTGGGGAAAAGCTTGGGGGTGACACTCACTTTGACGATGATGAGATGTTTTCTACGGACAATAAA

GGATACAATCTCTTTGTTGTTGCTGCTCATGAGTTTGGCCATGCGTTGGGATTGGATCACTCCAGGGATC

CTGGATCTCTGATGTTTCCAGTTTACACTTACACAGAAACAAGTCGATTTGTGCTTCCCGACGATGATGT

GCAAGGAATTCAGGTTCTGTATGGTCCTGGAAATAGAGATCCACATCCAAAACACCCTAAAACTCCAGAA

AAATGTGATCCTGATCTAAGCATTGATGCTATCACGGAACTAAGAGGGGAAAAGATGATCTTTAAAGACA

GGTTTTTCTGGCGGGTTCACCCACAGATGACAGATGCAGAACTTGTACTGATCAAGTCCTTTTGGCCCGA

ACTTCCCAATAAGCTTGATGCTGCCTATGAACACCCAGCCAAGGATTTGAGCTACTTATTTAGAGGTAAA

AAGTTTTGGGCTCTCAATGGATATGACATAGTGGAAGATTATCCCAAAAAACTCCATGAGCTTGGCTTTC

CAAAGACTCTAAAGGCCATTGATGCGGCTGTGTATAATAAAGACACTGGGAAAACTTTCTTCTTCACTGA

GGACAGTTATTGGAGTTTTGATGAAGAAGCCAGGACCTTGGATAAGGGTTTCCCGAGACTGATCTCAGAG

GACTTCCCAGGAATAGGAGAGAAAGTAGATGCTGCTTATCAGAGAAATGGATATCTCTATTTCTTCAACG

GAGCTCTGCAATTTGAATACAGCATCTGGAGCCAGAGAATAACACGCATCCTGAAAACCAACTTTGTGCT

GATGTGCTGATCTTGAAAAAATAATTTATACAAATATATATATATCAGGCTGCACTGGTCTTCAACAAAG

CTATGGGTTGGCTATTAAGGGGTGATATAGCGCAGGTTAGCTACAGAGTACAGTAACATATTCCTTTCTT

CCTAATTTTTTCCAGATTTCATATGGATGTTATTATTTATATATAACGCTACAAGTACCTACTGGTATTT

ATACATAACTATGTATCATCATGGAAGCTCACCTGTATATGTTCAGCACATAGGGTAGTAAGAGAGTCAT

GGGACATCATATTAAAAAGGAACAATGCAATTGGTTGCTCTCTGAACAACTGATTATTGTAACTAGGTGA

CTGGTCCGATATAGATTGATTCCTTCTAGCCGGAGTGACTGATTGTGGCAGTGAAACACAGCAAGACATT

TACCTATTGGACATTGCCCTAAAGGGGTGTGTCATCTTTTTAAGAATTGTATTCCTTTTCAAATTCTATT

CCATTGTCTCTAATACTTTTCCTTTGTTGACTATGGTGTGATGGGATGCTGCCCAAGGACATCCCTGGAG

CTGCACTAGATGGAGCATCCCATTACACCTCAGCCCACAATGGAAATAATAGCAGCTAAATCAATCTGAA

GTAATCAAGTGTTGGTGCAGTTCACTTATCTAGCAGAACTGTGGTGAGACATTCCTACAAATAAATATGT

TACATATTATCAGTCCAGAATTAAGGCATCTTACGGTGTGCAGTCTAGATAGACTCATCTTCTGATTAGC

AACTCCCCAAGTCTTGGTCCTCCTATTTATTGTCCTGGAATAGTAATGCTATCCTATAACGCAAGGGTCC

CTAAACTTTTTTACCCATGAGCCTCATTCAAATGTAAAAAAGAGTTGGGAGCAAACACAATCATGAGGAA

GGTTCCTAGGATGCCAAATAAGGCTATGATTGGCTATTTGGTAGCCCATATGAGGACTGGCAGACCTACA

GAAGGTTTTTTTTTGTTGGAATTCAAAAATAAGCACCTGCTTTGAGGCCACTGGAGCAACATCCAAGGGG

TTGGTGAGCAACAAGTTGCTGAGTCACTGGTTGGGGATCAGTGTTATACAGAATCTACTAAGGGGCTTTC

CACTTCTTCATTCTACAGATAATATACAAATAGATGTGTGTTTATGTTCACCTTTTTGGAGTTCTGTTCT

TTTGGGTTAATATATAGGACTATATTTTTTTCAAGTATTTTAACTATTTATTATATGTGAGAGAGCAGTG

CATTTCTAACAATGGAAAGACTTACAATCCTTGAAATCAAAACGTTCTATATTTTTCATAACATGATATT

CAGCCAAACATTAAGGATAAATGCAGAGTCTGTCACAGCTGCATCCCCTGGGAGCTGGGTTCTTTTTCTA

AATCTAATGCCTTTTTCTACATGGTATATTTATTAAAAGGAATATCCAAT

**38. Xl-MMP13A cDNA (U41824):**

AAGCCAAGC**ATG**GCTCCTTCATCCTTGTCAGTGTTTGTCCTATCTCTGAGCTTCACTTATTGCCTTTCAG

CCCCTGTTTCACAGGATGAAGACTCTGAACTGACACCAGGAGCCCTACAGTTGGCTGAGCATTACCTAAA

CAGGCTTTACAGTTCGTCGTCTAACCCCGCGGGCATGCTGAGGATGAAAGATGTGAATAGCGTAGAGACC

AAGCTGAAGGAGATGCAGTCCTTCTTTGGTTTGGAGGTGACTGGGAAACTCAATGAAGATACTCTGGACA

TCATGAAACAGCCAAGATGTGGTGTCCCTGATGTTGGGCAATACAACTTCTTTCCAAGAAAACTAAAATG

GCCAAGAAATAACCTGACATACAGGATTGTGAACTACACCCCAGATTTATCCACCAGTGATGTGGATAGG

GCCATCAAAAAAGCACTGAAGGTGTGGAGTGATGTCACGCCACTGAACTTCACCAGGCTCCGCACAGGCA

CTGCTGACATCATGGTCGCTTTTGGCAAGAAAGAGCATGGAGACTATTATCCATTTGATGGACCCGATGG

CTTACTAGCTCATGCCTTTCCACCTGGGGAAAAGATCGGGGGTGACACTCACTTTGACGATGATGAAATG

TTCTCAACGGACAATAAAGGATACAACCTCTTTGTTGTTGCTGCTCATGAGTTTGGCCATGCATTGGGAT

TGGATCATTCCAGGGATCCAGGATCTCTGATGTTCCCAGTTTACACCTACACAGAAACAAGTCGCTTTGT

ACTTCCCGATGATGACGTGCAAGGAATTCAGGCTCTGTATGGCTCTGGTAATAGAGATCCACATCCAAAA

CACCCTAAAACTCCCGAAAAATGTGATCCTGATTTAACCATTGATGCTATCACAGAACTAAGAGGAGAAA

AGATGATCTTTAAAGACAGGTTCTTCTGGCGGGTTCACCCTCAGATGACAGATGCAGAACTTGTTCTGAT

CAAGTCCTTTTGGCCTGAACTTCCCAATAAGATTGATGCTGCCTATGAGCACCCAGCCAAGGATTTGATC

TACATATTTAGAGGTAAAAAGTTTTGGGCGCTTAATGGATATGATTTTGTGGAAGATTATCCTAAAAAAC

TCCATGAGCTTGGCTTTCCAAAAACTCTAAAGGCTATTGATGCAGCTGTGTATAATAAAGCCATTGGGAA

AACTCTCTTCTTCGCTGAGGACAGTTATTGGAGTTTTGATGAAGAAGCCAGAACCATGGATAAGGGCTTC

CCGAGACTGATTTCAGAGGACTTCCCGGGAATCGGAGAAAAAGTAGATGCTGCTTATCAGAGAAATGGTT

ATATCTATTTCTTCAACGGAGCTCTGCAATTTGAATACAGCATCTGGAGCAAGAGAATAACACGCATCCT

GAAAACCAATTTTGTCCTGATGTGCTGATCTTGAAACTAATAATTTATATATATATATGTATCTATCAGG

CTGTTGAACGGCCTCATTTTATGCAGTTGACTACCACAATGGTCTTCAACAAAGTTATGGTTTGGATTTT

AAGGGGTGATATGCGCAGGTTAGCTACAGAGTACAGTTACATATTCCTTTCTTCTACACTTTTCCAGATT

TCATATGGATGTTCTTATTTATATATGACACTACACGTACATACTGGTATTTATAACTACAATGCAATTA

CTGGGTATCATCATAGAAGCTCACCTGTATTACATTTTGAAAGCCAATATCAGTGACCATGCTTGATTAT

GTTTCAGCGGATGGGTTAGCGGAAGAGAGTCAAGGGACATCATACAGTAGAAGGAACTACTTGCGACTTG

CCATTGAAACATTTACCCACTCGGTATTATCCCTAAAGGGTGGTGTTATCTTTTTTAGGAATTGTATTCC

ATTTCAAACTCTATTCCATTGTCCACTCATAGAAAGTACTTCTTCCTTTGTTGTCTATGGTTTGATGGGA

TGTTGCTCAAGGACAACCACTGGAGCTGAATTAGATGGAACATCCCATTATACCTCAGTCCACAATGGAA

AGAAAAGCTAAACCGATCTGAAGCAATCAAGAGTTGGTGTGGCCTAAGCCACTCATCTAGTAGAAACTGT

GGTGAGATATTTCTACATATAAACATGTGGCATAATATAATTCCAGGATTCAGGCATATTGCTGTGTACA

GTCTAGATTGCCTTGTCTTCTGAATGGCAACTCCCCAAGGTCCTCCAAGTTATTGTTCTTGAATGGTAAT

CTTATAGGACGGGGGTCCCCAACCTTTTTACCCGTGAGTCACATTCAAATGTTAAAAACAGTTGGGGAGC

AACACAAACATGAAGAAAGTTCCTGTGGGTGTCAAATAAGGTCTGCAATTGGCCATTTGGTAGACTCTAT

GTGGACTGGCAGCCTACAGGAGGCTCTGCTGAGTAGTACACCTGGTGTTTATGCAATTAAATCTTGCCTC

CAAGCCAGGAATTCAAAGCACCTGCTTTGAGGCCACATCCAAAGAGTTGGTGAGCAACATGTTGCTTGCG

AGACACTGGTTGGGGATCACCGTTATAGAGAATCTACGCACAGGCCTTCCACTTCTTCATTCCACATATA

TTATACAGATAGAGGCACTGTGTGTTCATGTCCACCTTTCTGGAGTTCTTTTCTTTTGGGTGAATATATA

AGACTATATTTTTGTAAGTATTTTCGATATTTTTTACATGTGAGAGAGCAGTGTATTTCTAGCAATAGAA

AGACTTATCACCCATGAAATAAGAATGTTCAATACTTTCCATGATATTCAGCCAAACATTAAGGAGAACC

AGATTATGTGCAGTCTGTAATGGCCGTATCCCTTTGTATTGTGCCCATATGTTTCTGTGTGCCCTGGGAG

CTGGGTTCTTTTTTTAAATCGAATGCCTTCTTTCTACATGATATATTTATTAAAAGGAATAACTAATAAA

TAAAAAAAAAAAAAAAAAAA

**39. Xl-MMP14A cDNA (AY633953):**

GGCAGTTGAAGAGGGAGAGAGGGATAGTGAGGGGAATAGCATAGTGGGGGCAGTCACAGCAAATATACTC

GGGAGGGAAAGAAAAACTTGATATAGCATGCACTGTGAATAACCTTTACAAATGACTCACTTTAAAAAGA

GTTAAAACATAGTTTGAACTCTGAAGGGGGGTTAAGGGGACTGAACAGAGATCAGTGGATAAGAACCCTA

TTCTTGCTCTGCAGACAACCACTCTTCAGAGGAGCTTTTACTCAGTCGCACAGG**ATG**GAGCCTCTGAGAG

CAGCCTGGATTTGCCTGTTTCTGTGTTCTGTGTGCAGCTCCAACCCTTCCAAATTCAGCCCAGAGGCCTG

GCTTCAACAGTATGGATATCTGCCCCCTGGAGATCTGCGGACACACACCTTACGCTCCCCTCAGTCCATG

AGTGCTGCAGTCTCTGCCATGCAGAAGTTCTATGGCTTAAAAGTGACAGGAAAGTTTGACAGTGACACAG

AGAAAGCAATGAAGAAGCCACGATGTGGAGTCCCTGACAAATTTGGTTCTGAAATAAAGGCAAATGTGAG

ACGGAAGAGATACGCCATCCAGGGTTTGAAGTGGCAGCACAAGGACATCACATTTTGCATACAGAATTAC

ACTCCCAAAATTGGCGAGTATTCTACCTATGAGGCGATTCGGAGAGCCTTTAAAGTTTGGGAGAGTGTGA

CGCCACTTCGCTTCCGGGAGGTTCGATATGTGGATATTAAAGATGGATACACTAAACATGCTGACATCAT

GCTTTTTTTTGCTGAGGGTTTTCATGGAGACAGCACTCCTTTTGATGGAGAGGGGGGCTTTTTGGCACAT

GCATATTTTCCTGGGTCAGGCATTGGAGGTGATACACACTTTGACTCTGCAGAACCTTGGACAGCTAGGA

ATGATGATCTTGAAGGTAATGATCTGTTCCTGGTGGCTGTACATGAACTTGGCCATGCTCTCGGTTTGGA

ACATTCGAATGATCCATCTGCAATTATGGCTCCATTTTATCAGTGGATGGACACAACTAATTTTCAGCTA

CCAGATGATGACCGCAGAGGAATCCAACAGCTCTATGGTCCCGATCACGGAGAAGATCGTCCAACTCGTG

CACCTCGTCCTACACGAGCTCCGCGACCCACACAAACCCAGAGACCAGATGATAACCCACATGATCCCAC

TTATGGACCTGACATCTGCCAGGGCAACTTTGACACAATTGCTGTGTTAAGGGGAGAGATGTTTGTATTC

AAGGAACGCTGGTTCTGGCGTGTACGTCATAAACGTGTAATGGATGGATACCCTATGCCTATTGGGCAAT

TCTGGCGTGGTCTTCCCAGCTCCATTAATTCAGCTTATGAGCGAAAGGATGGCAAATTTGTGTTTTTCAA

AGGGGATAAGCACTGGGTGTTTGATGAAGCTGTTTTAGAGCCAGGGTATCCCAAAACCCTGAAGGAGATG

GGCAGAGGACTTCCTAGCGACAGAATTGATGCTGCCCTATATTGGATGCCAAATGGAAAGACCTACTTCT

TCAGGGGTACCAAGTATTACCGGTTTAACGAGGAGATGAGAGCAGTGGATCCAGAATACCCCAAACCTGT

CAACGTGTGGGAAGGCATCCCAGACTCCATCAAAGGAGCATTCATGGGCAGTGATGGAGCTTTCACTTAC

TTTTACAAGGGTAACAAGTATTGGAAGTTCAACAATCAGCAGCTGAAGACAGAGTCTGGCTACCCGAAAT

CCGTTTTGGTCGACTGGATGGGCTGCAGCACTGTCCGACAGCCGGAAGAGGATGATGACAGGGAGGTGGT

TATTATTGAGGTAGACGAGGCAAGTGGTGAAGTGAGTGCTGCAGCCATTGTAGTTCCTGTCATTCTCTTG

CTTTGTGTTCTTACCCTTGGACTGGCTGTTGTGCTCTTCAGGCAATGTGGCACACCGAAGAGGATTCTAT

ACTGGCAGCGGTCCCTCCTGGATAAAGTGTGAACACCTACACTTAGAAATTTGCACCCTCCCCCTGAATT

ACTGCCTCTCCAGCCCTCCTCTGTCTGCCTAATACAAATGATACAGGGAGCAGAATCAGCTTCCAGGAAA

GAGAGAAGTGCTAATGCAATTTGCACCAACTTTGCCTCTTATTATTTTTTCTAGTGGCACGTGTTGTGTT

TAACCCCTTCACTGCCAGACTGGGGCCAAGATGATTGAACAGATTATGATGTGTTTGGTCATTAGGGCCT

GTTCTCTCAAATAACAGCAAGTTCCAAAAAAAAAAAAAAAAAAAAAAAAAAAA

**40. Xl-MMP14B cDNA (BC077870):**

CCCACGCGTCCGCGGACGCGTGGGGAGAAGGATAGTGAGGGGAATAGCGTAGTGGGGCAGTCACAGCAAA

GATACTAGGGAAGGAAAGAAAAACTTGATATAACATGCACTGTTAATAACCTTTGCAAATTACTCACTTT

AAAAAGAGTTAACACATAGTTTGAGCTGTGAAGTGTGGTTAAGGGGACTGAACAGAGATCAGCGGATAAG

AAGCCTATTCTTACCCTGCAGACAACTACTTTTCAGAGGCTCTTTTACTCAGTCGCACAGG**ATG**GAGCCT

CTGAGAGCAGCCTGGATTTGCCTGTTTCTGTGCTCTGTATGCAGCTCTAGCCCTGCCAAATTCAGCCCAG

AGGCCTGGCTACAACAGTATGGATATCTTCCCCCAGGAGATCTGCGGACACACACCTTACGCTCCCCTCA

GTCCATGAATTCTGCCATCTCTGCCATGCAGAAGTTCTATGGGTTAAAAGTTACAGGAACATTTGACAGT

GACACAGAGAAAGCAATGAAGAGGCCACGATGTGGAGTCCCTGACAAATTTGGTGCTGAAATAAAGGCAA

ATGTGAGACGGAAGAGATATGCCATCCAAGGTTTGAAGTGGCAGCACAAGGACATCACATTCTGCATACA

GAATTACACTCCCAAAATTGGCGAGTATTCTACCCATGAGGCAATTCGGAGAGCCTTTAAAGTTTGGGAG

AGTGTAACACCACTGCGCTTCCAGGAGGTTCGATATGTGGATATCCAAGATGGATACACTAAACATGCTG

ACATCATGTTATTTTTTGCTGAGGGTTTCCATGGAGACAGCACTCCTTTTGATGGAGAGGGGGGCTTTTT

AGCACATGCATATTTTCCTGGGACAGGCATTGGAGGTGATACACACTTTGATTCTGCAGAACCTTGGACA

GCTAGGAATGAGGATCTCGAAGGTAATGATCTGTTCCTGGTGGCTGTACATGAGCTTGGTCATGCACTTG

GTTTGGAACATTCAAATGATCCATCAGCAATTATGGCTCCCTTTTATCAGTGGATGGACACAACGAATTT

TCAGCTACCAGATGATGACCGCAGAGGAATCCAACAGCTCTATGGCCCTAATCATGGAGAAGGTCCTCCA

ACTCGTGCACCTTACCCTACAAGATCTCCCACACAAACCCAGAGACCAGATGATTCCCCACATGATCCCA

ACCCACCCACTTTTGGACCTGACATCTGTCAGGGCAACTTTGACACAATTGCTGTGTTAAGGGGAGAGAT

GTTTGTCTTCAAGGAACGCTGGTTCTGGCGTGTACGTCATAAACGTATAATGGATGGATACCCTATGCCT

ATTGGACAATTCTGGCGTGGTCTCCCCAGCTCCATTAATTCAGCTTATGAGCGAAAGGATGGCAAATTTG

TGTTTTTCAAAGGTGATATGCACTGGGTATTTGATGAAGCTATCTTAGAGCCAGGTTATCCTAAAACCCT

AAAGGAGATGGGCCGTGGACTTCCAAGCGACAGAATTGATGCAGCCCTCTATTGGATGCCAAATGGAAAG

ACCTACTTCTTCAGGGGAACCAAGTATTACCGGTTTAATGAGGAGACGAGAGCAGTGGATCCAGAATACC

CCAAGCCTGTCGAAGTGTGGGAAGGCATCCCAGACTCTATCAAAGGAGCATTCATGGGCAGTGATGGAGC

CTTCACTTACTTTTACAAGGGTAACAAGTATTGGAAGTTCAACAACCAGCAACTGAAGACAGAGTCTGGC

TACCCTAAATCCGTTTTGGTCGACTGGATGGGCTGCAGCACTGTTCAACAGCCAGAAGAGGATGATGACA

GGGAGGTGGTTATTATTGAGGTAGACGAGGCGAGTGGTGGAGTGAGTGCTGCAGCCATTGTCGTTCCTGT

CCTTCTTCTGCTTTGTGTTATTTCCCTTGGACTGGCTGTGGTGCTTTTCAGGCAATGTGGCACACCGAAG

AGGATTCTTTATTGGCAGCGGTCCCTTTTGGATAAAGTGTGACCACCTACACTTAGAAATTTGCACCCTC

CCCCTGAATTACTGCCTCTCCAGCCCTCCTCTGTCTGCCTCATACAAATGATACAGGGAGCAGAATCAGC

TTCCAGGAAAGAGAGAAGCGCTAATGCAATTTGCACCAACTTTGCCTCTTACTATTTTCTCTAGTGGCAC

GTGTTGTGTTTAACCCCTTCACTGCCAGACTGGGGCCAAGATGATTTAACAGATTATGATAAGTGTTTGG

TCATTAGGGCCTGTTCTCTCAAATAACAGCAAGTTACAAAAGTCATGACAGAGAGGGACATGAACTTGAG

CTTTAAAAGGACTAATACTGTTTAGTTTTTGAAATTCAAAATAAATATGAGCCTTCGTTCTTTTCCTTCC

TACAAACACCCAATGTTTTATCACCAGCCAGAAGTAAAGAGCCCTGTTCCTAGCTGGGTATGAATGTGCT

AGTCATAAGGATTTTTTTTTGTCCAGGAATATGGAGGTGTACACTCAGGAACATACTCCTTGAACTGCAT

GGGATTTGATATAGGACACACAATGTTTTAAATATTGATTAGAAGGTCAGTGTCATGGTAATTATACATT

AAAGGCATTGATATGAATGATGAATTAAACTACAGGAGAGCACATTAAACTGGCTGTTAACCACAAGTCT

TGGTTACTAAGCTTTTAGATATGCCAAATAATTCTGCCAGTCCCATGGAAACTACAGATAAACTGTATAA

GTGTCCTAAGGGGAAGATATATAAAACTTGTAGTAGCTTCTCTCCACTTACGTATTGCTGTAACCCAACG

GTAGTTAGTATGTTGTGTGATCCCTTCATATTCCTGGACAGTATACCCTAAAAAATAGCCCCACCACTTA

ATCAGTAGAAAAGCCAGTGATGGGATTGTTTTGTAAGGTGTGACATGGGGACTGTTGAGGAGTTAGTTTC

TCTCTTTTTTTATTTTTTTTTGCAGAGCAGGAATCTGTATATAGAAGTGGGGGGGGGGCAAGTCTAAGAT

AAACATTGCCCAAATCCTTTAGTGCCTTATTAGGTGTCCCTCAACCACCCATTTTCTTCTATGACGCCTC

CTCTAAGCCCTCCACCTTTATAATCTGATGATTCTGTAAGGGAAAGGGTTAATGTGCAGCTCCCTGCTAC

CTACAGATCATTGCTGAGGGATGGACATTGCTCTGTAGGTACTTTGGAAGGTATTGGTTATTTTTTTTTG

TATACAATCTGGCAGCAAAGGGGTTATTCACTGCTCGTTGGGTCTAGCAGGAAGGCATTACATCATGCTG

TGCTAGCAAAAGTGTCCATTCCAGTGCTAAAGAAAAGATTATCCATCTCTGTTAATCGCAGCTATGGGTG

TCTTTGGAAGGAAAGGGGTGACGAAGGGCAAATCACATGGTACGGTTAAGGGGGTGGGGATCACATTTGC

TTCTTTCCCTTCTCCTATTCTTTGGTTTATATTTTTAATATTATAATTAATATTATTTGGTAATGAGGCT

TCATGTATTTGCACAGCCCCTGCAGGTCAGGTGGCTTTGTGTTTTACACCTGCCTACTGCAAGAGGGGTC

TATGGAGCAACTCAGGTCTTGGTATAAAGAGCAGCGCTCTGCTTGCCCCTTCTGCAGTGAATGGGTGTCT

TATTTTCCCCTACCCTTAAAAGCCACACTGAGGCCAGTGCTTGCACATTGATTTGGGGGGGGGGCAACCT

GCCAACCTCCAACCCAGACCCCAAGTGCATTATCACTGACTCATACAAAGACTAGAAAGGGCAAACTTAC

TGCAGTATTCCCTCAATTTAATGTATTGGAGTATCTGGGGCCCCGTTGTTGGGTTAATTAAAGCTTGTCG

AATGGACAAATTCAGTTTTTTAATTGTTTTTATGTGTCCTTTTTTATCTAAAGAAAACAATTTGTTATGT

CCAAAAAAATTTGTAACATTGGAACCATTAAATGCCAAAATGTGCTAAAAAAAAAAAAAAA

**41. Xl-MMP15 cDNA (AY573378):**

GAATTCGGCACGAGGGTTTAAGGGTGCCTGGTTTTGGAGGGTACGACACAACCGAGTCCTGGATAACTAC

CCC**ATG**CCCATTGGTCACTTCTGGCGAGGGCTGCCGCCTAACATTACTGCTGCCTATGAGCGACATGATG

GAAAATTTGTCTTCTTTAAAGGAGAGAAGTACTGGCTGTTCCGAGAGGCCAACCTGGAAACTGGTTACCC

GCAGCCGCTGACCAGCTTTGGATATGGGATCCCTTATGACCGAATTGATACAGCCATATTTTGGGAACCT

ACAGGACACACATACCTCTTCCGGGGAGACAAATACTGGCGTTTCAATGAGGAGTCTCGTTCTGCAGACG

TTGGTTACCCAAAACCCATTACAGTCTGGGCTGGTATCCCTGACACCCCCAAAGGGGCCTTCTTGAGCTC

TGACTCGACTTACACTTACTTCTACAAAGGTGCCAAATACTGGAAGTTTGATAACCAGCGGCTGAAGACA

GAGCCAGGATACCCTAAATCCATCTTGAGAGACTTTATGGGGTGCCAAGAAGAAGTTTTTCAGGATCCGG

ATGTTGTTCCACGATGGCCAGATGTTGAACGTCCCCCATTTAATCCTGATGTTGAGTCAGAGGATAAAGA

TGGCAAAGACAGTGACAGCGCGGGACGGGAGGACCCAAACACTGGACGTGATGTGGACGTTGTGGTCCAT

ATTGACGAGTACACACGGGCCGTTAGTGTTGCCATGATTATTGTGCCACTACTACTAATACTGTGCATTC

TGGGACTTATTTATGTCATTGTGCAGATGCACCGGAAAGGACCCCCTAAAGCCTTACGATACTGTAAACG

CTCTTTACAGGAATGGGTCTGATTTTCTTTTTTTTTTTTTTTTTTTTATTGTTGCCATTTTTTACCTCTC

ACCATCCACCTATTTCTTTTGTTCCCTCTTTATTTTTATATTCACAACTCATTTTGTGTGGTGCTAACAT

GGGGGAGCTGCGATATTTAATGTTAATATTTGTTCCAACCCCCTCCTGAGTTTCCATTCCACTGTTTCTT

GACTGTGGGGGCAAAAGAAGGAATACTGCATTTTCTGACACTTTAAAGGGTGCGTCATATTTCCTGGTGT

TCGTTTCAGATTCATTGGTCCACCTGTGTGATGCAGGGGTGATGCACAATACATATATCCCTGATAGTGT

AACACATTAATCTTTGCATCGTGTGGTTGAGCCTTCCAAGGTGCATTTCTGCGGGTCATCCTTATGCCGA

TGCTGGCAGGAGATAAATGACCCTTTTAGTTGTAATTATTCTGTAGCCCTAGCACTGTGTGGATACAGTT

GAATGTTTCTCTAGCATTTAGGATGTTGAATATTTATGCCATGCATTTTGCACTGGAGATATCCTAGGGT

GGGTGCAGATCAGCAAAGGGAGGAATACACAGACCTGAATGGGTAAAAATAGTGATTCTACCTGCACTCT

GGCTGCTCAGATATCATGAAAGGTACCTCAAAACTGGGGAATATCTAAGCCTGGCATGAAAGGGTTATGG

TGCCTTAATCACCTGTGATGATCCCCTCACATAAGAGGCCGAGTCTCGTAAAGAGCCGGGATAGAGATAT

TTTCCTTTGCGTATGGAAGGGAGAGCGTTGTTCCTGTGGAATGCTGGTATTAGTAAGTTAATAAGGAGTT

GCTTTCCTGCACTCCCTGATATTGCTCATATCGGGCTGTGCCCATGAAGTCTATGAACAACAGTCTCAGT

TTCTTCAGTCTCTGTACGTTAGATGTTCACCCGGATGTTTAAAAAAAAAAATGTGTTGTTTTTATAAGAT

TTTGTACAGTTTTAAAAAAAAAAAAAAAAAAAAAAAAAAAAAAAAAAAAAAAAAAAAAAAAAAAAAAAAA

A

**42. Xl-MMP16 cDNA (AY310397):**

GGCACAGAGACCTGGGGAACGTGTTTCACTTGGCCGCTGGTTATGAATGAGATCTCCCCCGGTGTGCGTG

CCACCGGAGCTCTCCATCGCTCACCTCGTATCGCAGCAGCGCAGCATCTCCTATGCTCAGATAATCCCGC

ACGGCTGCTCTCCTCCAGCACCGGGGACTTTGTGAAGGAAGCAGCCGCCAGTCTCCCGGAGCAGATAGAT

CAGCTCACC**ATG**GTCTGGCTCCCCTCAGGCACTGGCAGCCCGTTACATTTGTGCCGCTCCGTGGGGCTTG

TTGTGCTCACCTTGCTGGGGATCATTGTATGTTGCGTGTGCGCAGGGGAGCAGCAATTCAGCGCAGAGAT

GTGGCTTCAAAAATATGGCTACCTTCCACCAACTGACCCCAGAATGTCAGTCCTGCGATCTTCAGAAACC

ATGCAATCCGCTATAGCTGCCATGCAGCAATTTTATGGGATCAATGTTACGGGAAAGATCGACAAAAACA

CAATTGATGAAATCACATTGAGTTGGATGAAAAAACCACGGTGTGGAGTTCGTGATCAAGCAGGACCTAT

TTCCAGATTTAATGTCCGACGAAAACGATATGCCTTAACGGGACAGAAGTGGCATCACAAACATATCACC

TACAGTATAAAGAACGTTACTCCAAAAGTGGGAGATTTGGAAACCCGTAAAGCCATTCGACGTGCCTTTG

ATGTCTGGCAGAATGTAACTCCGCTGACATTTGAAGAAGTTCCATACTGTGAATTAGAAAATGGCAAACG

GGACGTAGACATCACTATTATTTTTGCATCAGGTTTTCACGGAGACAGTTCTCCTTTTGATGGAGAGGGG

GGATTTCTGGCACATGCTTACTTTCCAGGACCTGGCATTGGGGGAGACACACATTTTGACTCTGATGAAC

CGTGGACTTTAGGGAATCCTAATCACGATGGAAATGACCTATTTCTAGTTGCAGTACATGAATTGGGTCA

CGCATTAGGCCTAGAACATTCCAATGACCCCACTGCGATAATGGCTCCCTTTTATCAGTACATGGAAACA

GACAACTTCAAGCTACCTACTGATGACTTACAAGGAATTCAGAAAATTTATGGTCCACCAGAAAAGGCAC

CAGCACCAACTAAACCCCTTCCTACCGTGCCACCACACCGTTCTGTTCCTCCAGTAGACCCTCGCAAGAA

TGACAGACAACCTAAACCACCCCGGCCGCCTACTGGAGACAAGCCATCTTACCCTGGAGCCAAACCCAAC

ATCTGCGATGGGAATTTTAATACGCTTGCAATCCTACGCCGAGAGATGTTTGTTTTTAAGGATCAATGGT

TCTGGCGTGTAAGAAACAACAAGGTTATGGATGGCTACCCCATGCAGATTACCTACTTCTGGAGGGGACT

GCCTCCTGGCATTGATGCAGTTTATGAGAACGGCGAGGGAAATTTTGTCTTTTTTAAAGGTAATAAATAC

TGGGTGTTTAAAGATACAACACTTCAGCCTGGCTACCCTTATGATTTGATGCACTTAGGACACGGCATTC

CACCTCATGGTATTGATACGGCTGTTTGGTGGGAAGATGTTGGGAAAACCTACTTCTTCAAAGGTGACCG

GTATTGGCGATACAATGAGGAAATGAGAGCAATGGATCCTGGCTATCCAAAGTTAATCACAGTATGGAAA

GGAATTCCAGAGTCACCACAAGGAGCCTTTGTTGACAAAGAAAATGGCTATACATATTTTTATAAAGGAA

AAGAGTACTGGAAATTCCAGAATCTCAATCTCCGGGTAGAGCCTGGGTACCCGAGATCGATCCTTAAGGA

TTTCATGGGATGTGATAGTTCCACTGATGGAGACAAAGAAAGAACCAGCCCGCAAGATGATGTAGACATT

GTCATCAAGCTGGACAACACAGCCAGCACTGTGAAAGCCATAGCCATTGTAATCCCATGTATACTGGCCT

TGTGCCTTCTTGTATTGGTTTACACTGTGTTTCAGTTCAAAAGAAAAGGAACACCCCGCCACATACTTTA

CTGTAAACGGTCTATGCAAGAGTGGGTGTGATGTAGGGTTTCTCTCATAGAAATTACTTGAGGTTCAACA

TGAGAGCTATTACGCTGTTCCCTAGCTAGAAGCAGGCATCTGTGATCCAGGCTCATAGTCGATCTTAAAA

ACCACAAGCGGTTTGGTGTCCTGCACTTGAGTGGGGATTCAATCATCTGGGAAGCTTCCATGAAATACGG

TTTCTGCTGTTCCTCCAGTCCTTTGTATTTCTTTATCATTCACTTTTAGGCCTTTCCCTCTGCACATTGA

ATGCTCAGTTTACTCTCGGAAGTAAACGCGAAGAGGAGAACAGATCAAAAAACGATAATAAAGTTTTATT

TTCGTTCCGGGGAAAAAAAAAAAAAAAAA

**43. Xl-MMP17 cDNA (CK806816):**

GGTATGTAGGGAGTGTAGGAAGTGATATCTGGACCTGGTGTCGGCAGCAGCAGTAGCCTC

CCACGTACACAGAGGAGGGACAGGCTGATCCGCCGCCTGCAGGTACCGGATTTTTTTTTT

CTACGAGTGCAGCTAATTACGGATTGGTTGACTAAATTTGGATATCTGCCCCCGCCCAAT

CCTGTTACAGGGCAACTGCAGACACAGGAAGAACTGTCTAAAGCCATCAAGGCAATGCAA

GAGTTTGGTGGCCTGAAAGCTACTGGGATTTTAGATGAGCCAACTTTGATGCTGATGAAA

ACCCCTCGTTGTTCCCTGCCTGACCTGTCCCACTCACAGGCTTTAAGAAGGAGACGAAGT

GCCCAGCCCCTGACAAAGTGGAATAAAAGGAATTTGTCCTGGCGGGTTCGAAATTTCCCT

AAAGAGTCTTCACTTGGACATGACACTGTGCGGGCACTGATGTATTATGCCCTTAAAGTA

TGGAGTGATATTACCCCACTGAATTTTCATGAGGTGGCAGGGAATAATGCAGATATTCAA

ATAGATTTTTCAAGGGCTGATCACAATGATGGATACCCATTTGATGGCCCTGGTGGGACA

GTAGCTCATGCCTTCTTCCCAGGAGAACATCATACATCTGGAGATACTCACTTTGATGAT

GAAGAATCTTGGACCTTCCGGTCATCAGATATTCACGGCATGGATCTGTTTGC

**44. Xl-MMP18 cDNA (L76275):**

ACCTTCAAATCCTAGAAAGCCTTAGAAGA**ATG**AACAGCCTCCTGCTAAAGCTGCTACTATGTGTAGCCAT

TACTGCTGCCTTCCCAGCAGATAAACAAGATGAGCCCCCAGCAACAAAGGAAGAAATGGCAGAGAATTAC

TTGAAGAGATTTTACAGTCTTGGAACTGATGGGGGACCAGTTGGAAGAAAGAAACACATCCAACCTTTCA

CTGAAAAGCTCGAGCAGATGCAGAAGTTTTTTGGCTTAAAGGTGACAGGAACATTGGACCCCAAGACTGT

AGAAGTAATGGAGAAACCCAGATGTGGAGTCTATGATGTTGGCCAGTACAGCACAGTCGCAAAAAGTTCT

GCATGGCAGAAGAAGGATCTGACCTACAGAATTCTAAACTTCACTCCTGACCTGCCTCAGGCTGATGTGG

AGACTGCCATACAAAGAGCTTTCAAAGTCTGGAGTGACGTGACACCTTTGACCTTTACCAGAATCTACAA

TGAAGTATCAGATATAGAAATCTCCTTTACAGCTGGAGATCACAAAGACAATTCTCCTTTTGATGGATCT

GGTGGCATTTTGGCCCATGCCTTTCAGCCCGGCAATGGCATTGGTGGAGATGCCCATTTTGATGAAGATG

AAACCTGGACAAAGACCAGTGAAATATACAATCTGTTTCTTGTTGCTGCTCATGAATTTGGACACTCGCT

GGGGCTTTCTCATTCCACTGATCAGGGTGCTTTGATGTATCCAACATACTCAAATACCGACCCCAAGACA

TTTCAGCTTCCTCAAGATGATATCAATGCTATACAGTATCTATATGGAAAATCCTCCAATCCAGTCCAAC

CAACCGGACCATCCACTCCTTCCAGATGTGATCCAAATGTTGTTTTCAATGCTGTCACCACCATGAGAGG

AGAACTGATTTTCTTTGTGAAGAGGTTTTTATGGAGGAAGCATCCCCAAGCATCCGAGGCTGAACTCATG

TTTGTTCAAGCATTCTGGCCATCGCTGCCCACTAATATTGATGCGGCGTATGAAAATCCTATAACGGAGC

AGATCCTTGTGTTTAAAGGATCAAAATATACAGCTCTGGACGGCTTCGATGTAGTACAAGGTTACCCCAG

GAACATCTACAGCCTGGGATTTCCAAAGACCGTGAAAAGAATTGATGCGGCTGTTCATATTGAACAACTA

GGGAAAACATATTTCTTTGCAGCTAAGAAATACTGGAGTTATGATGAAGATAAAAAACAAATGGACAAAG

GCTTTCCAAAGCAAATAAGCAACGATTTCCCGGGAATTCCTGATAAAATTGATGCAGCTTTTTATTACAG

AGGCCGCCTGTATTTCTTCATTGGAAGGAGCCAGTTTGAATACAATATCAACTCTAAAAGAATCGTACAG

GTCTTAAGAAGCAACAGCTGGTTGGGCTGTTAATAAGACGGACCCCAAGCTCTGTAACAAGGGCAACGAT

GAATGTGATGGCAGCTCATTTCTGCACCTGCCGTGTGACCAATATTATTTAAAATCATTTCCTTTGAATG

TTTGACTTTATTTAATCTTTATGAATTAATATTTTGTACTCATGTTGTTTTAATAAAAACGTTTTGTTT

**45. Xl-MMP19 cDNA (BX847184):**

CAGGAGACAAACTGTGGGTGTACACCAATTTCAAGCTGAATCCTGGCTACCCTAAGCTGT

TAACAAGGGTTCCACCGAATATCAATGCTGCTTTGTATTGGGAAGTAAACAAGAAGATCT

TCTTGTTCAAGGGGGATGGTTACTGGCAATGGGATGAGTTGGGATGGAGCAACCTTTCCA

TGTATCCAAAGAAGATCTCCAGCCTGTTCACTGGCATTCCCTCTCAGCTGGATGCCACTG

TGACCTGGAAGAATGGCAAGATTTATTTCTTCAAGGGAGACAAGTACTGGAGAGTGAACA

AGCAGCTCAGGGTGGAGCGGGGTTATCCTCTGAGCAAGGCGGAACGCTGGATGCAGTGCT

ACTATTTTGATTAATGATGGACAAAATTAACTATTTTCAAAGTGCCTTATTGCCTGCAAG

GAGAAGTGACACTGGAGTCAGATATTATTTCTAAGCCATCAGAAAATCATGTATACAGTA

TTTGCTTTATTCCAGGGCTCTAGTCTGGGACTATTGAACAGAGCCTAGGCCAGAAGATAT

TTGCTATGTATAGTAGTTATAAGTGAATTGAATGAAGTCTTTAACACCTGAAGGGATACT

ATCTAAAGAGACACACTTTTTAGTCTGTCTCCAGC

**46. Xl-MMP20 cDNA (DQ885892):**

TTGGAAGTTGCCAGTTCATCCCCTTCAAATTTGGAAA**ATG**AGGGCCCTGGACATTTTGTGGATTTTGGAT

TTTTTGCTTTTTTGTTGGAAATTATCTTTAGCTGCGCCGTTGCGTTTTACATGGTCTCAACCTATCGACT

GGAAGGACATTTATACGGCAAAGCAATATCTTGACAAGTATTACAGTGACCGAGGGCCGGTGCGAGTGGC

TGAGATGGTGGCGGACGATGTCTCTATGTCGAGAAAGATTAGAAAAATGCAAAAGTTTTATGGCCTTCAG

GTTACCGGCAAGTTGGATCAATCTACACTCGCAGTCATGAAGAAGCCACGCTGTGGGATGCCAGACTTGG

CCAATTATCATGTCTTTCCTGGAGAGCCTAAATGGCAAAAAAGTACTTTGACTTACAGGATTACAAAATA

CACCACTAGCCTTAGTGCACAAGATGTTGACAGAGCTGTGGAGCTGGGGTTAAAAGCATGGAGTGATGCG

GCCCCTCTGAATTTTGTCAAAACAACTCAAGGAGAAGCTGACATCATGATTTCCTTTGAATCTGGAGATC

ATGGAGATTCTTATCCTTTTGATGGTCCCCGGGGAACTCTGGCTCATGCGTTTGCTCCAGGTGAAGGATT

AGGGGGCGATACTCATTTTGACAACGCTGAGAGGTGGACAACAGGAAAAAATGGGTTTAACCTTTTTACC

GTAGCTGCTCATGAATTTGGCCACGCGTTAGGTCTTGGACATTCAAGTGATCCTTCAGCTTTGATGTACC

CAACATATAGATACCAGCATCCCATTGGATTCCAGCTGCCAAAAGACGATGTCAAAGGGATCCAAGCTTT

GTACGGAACCAAGGGAACTGGGAAAGAAAAGCCTGCAGGACCTCAGCAACCATCAAATAATCCAGATCAG

TGTGACCCAAATCTATCTTTTGATGCTGTTACCGTTCTAGGAAATGAACTTTTGTTGTTCAAGGAGAGGT

CTTTCTGGAGGAGACAGGCCCAACTTGCCAACATCTGGCCAAGCCCAATCGCAAGCTCCTTCCCACAGTT

GATGTCCAACATTGATGCTGCCTATGAAGTTGCAGAACGAGGGACAGCATATTTTTTTAAAGGACCTCAT

TACTGGACAACAAGAGGATTACAAACTCAAGGACTCCCAAGGACCATTTATGACTTTGGTTTTCCAAGAC

ACGTACAGAAAATAGATGCCGCTGTTCACTTAAAGAACTCCAGAAAAACTCTGTTCTTTGTTGGTGATGA

TTATTACAGTTACGATGAAACAAGAAGGGCAATGGAGGATGATTACCCAAAGAGCATTGATGATGAATTC

CCAGGCGTGGAAGGAAATATTGATGCAGCAGTGGAAGTTAATGGATTTATTTACTTTTTCTCTGGTCCAA

AGGCTTATAAGTACGACACTGAAAAAGAGGACGTGGTTAATATTGTGAAATCTAGTTCCTGGATCGGATG

CTAAAAAAGTTCTCCAAAAATTGTTACAATCACCTCAGGATCATGAGGAAACTGGGTTTTGACATCAACG

CAAGAAGAATTAAAATCTTGAAAACACTAAAAAAAAAAAAAAAAAAAAAAAAAAAAAAAAAAAAAAA

**47. Xl-MMP21 cDNA (U82541):**

TCGTGGAGCACTACAGAATATGCCGCACAGAGTCAGCAGAACGGCAGCAGATGCATCAGCATCATATGAG

GCCACC**ATG**CCTTCTATCAAGCTTCTGGTTTGGTGCTGCTTGTGTGTGATATCCCCCAGGCTGTGCCATT

CTGAGAAGCTCTTCCACAGTCGGGATCGGTCAGACCTTCAGCCCTCAGCAATTGAACAGGCAGAACTGGT

CAAGGATATGCTCTCTGCCCAGCAATTCCTGGCAAAATATGGGTGGACACAACCAGTTATTTGGGATCCA

TCAAGTACCAATGAAAACGAACCTCTAAAAGATTTCAGTCTGATGCAAGAGGGAGTTTGTAACCCAAGGC

AAGAAGTGGCTGAGCCAACAAAAAGCCCCCAATTCATTGACGCGCTCAAAAAGTTTCAGAAGCTAAACAA

CTTGCCAGTGACAGGAACCCTTGATGATGCCACCATCAACGCCATGAACAAGCCACGGTGCGGCGTGCCA

GACAACCAAATGGCAAAGAAAGAGACGGAGAAACCGACAGCAGCACAGTCACTTGAAAACAAGACTAAGG

ATTCTGAGAATGTTACTCAACAAAACCCAGACCCCCCCAAGATTCGGAGGAAGAGGTTCTTAGATATGTT

AATGTACTCAAACAAGTACAGGGAAGAACAGGAGGCGCTTCAGAAATCCACAGGCAAAGTCTTCACCAAA

AAGCTGCTGAAATGGAGAATGATTGGAGAAGGCTACAGCAATCAGCTTTCCATCAACGAGCAAAGATATG

TCTTCAGGCTGGCTTTCCGCATGTGGAGTGAAGTCATGCCACTGGACTTTGAAGAAGATAACACCTCCCC

TCTATCCCAAATTGATATCAAACTTGGATTCGGACGAGGTCGCCATTTAGGCTGTAGCCGGGCATTCGAT

GGCTCTGGGCAGGAGTTCGCCCACGCTTGGTTTCTGGGGGACATTCACTTTGACGACGATGAACATTTTA

CTGCTCCCAGTAGTGAGCATGGGATTAGTCTGCTGAAGGTGGCAGCCCATGAAATTGGCCATGTTCTTGG

ATTATCTCACATCCACAGGGTGGGATCAATAATGCAGCCCAATTACATTCCGCAGGACTCTGGCTTTGAG

CTGGACTTGTCTGACAGGAGAGCCATACAGAACCTATACGGCTCATGTGAAGGCCCCTTTGACACAGCGT

TTGACTGGATCTATAAAGAGAAGAACCAATACGGGGAGCTTGTTGTTCGATACAACACCTACTTTTTTCG

CAACAGCTGGTACTGGATGTATGAGAATCGGAGCAACAGGACCCGATACGGGGATCCACTTGCAATTGCC

AACGGCTGGCACGGAATTCCCGTACAGAATATTGATGCTTTTGTTCATGTCTGGACCTGGACAAGAGACG

CCTCCTACTTTTTTAAAGGTACTCAGTACTGGCGCTACGATAGTGAAAATGACAAAGCCTATGCTGAAGA

TGCACAGGGAAAGAGCTACCCTCGCCTAATCTCAGAAGGGTTTCCTGGAATCCCAAGCCCCATCAATGCT

GCCTATTTTGACAGGAGAAGACAGTACATTTACTTCTTCAGGGACTCCCAGGTTTTTGCCTTTGATATCA

ACAGAAACAGAGTTGCGCCAGACTTCCCCAAAAGAATCTTGGACTTTTTCCCAGCCGTCGCAGCAAACAA

TCACCCCAAGGGCAACATAGACGTGGCCTATTATTCCTACACATACAGCTCCTTATTTCTCTTCAAAGGA

AAAGAGTTTTGGAAAGTCGTCAGCGACAAGGACAGGAGGCAAAACCCGTCTCTTCCATACAATGGATTGT

TCCCCAGAAGAGCAATATCTCAGCAGTGGTTTGATATCTGCAACGTACACCCTTCATTGCTGAAAATTTG

ATTTGCCCAAAATAGCCCAGCCTGAATAATAGTCATGCACTGAGGTTTTCTAAAGTCTTCTTCTGCCCTT

GTCCCACCTGTGCACTCACCTGTAGCATGGACAGCCCAAAATAGCCCAGCCTGAATAATAGTCATCCACT

GAGGTTTTCTAAAGTCTTCTTCTGCCCTTGTCCCACCTGTGCACTCACCTGTAGCATGGACATACTCCCA

GATGATTGACCTTTCTACAACTCTACTTCCAGAGTGTCCATGTCTAAGAATTTCTTAGTAAAAGAGTTGA

CTAGACTATTGTATTGTTTCCTAAACGCAGCCATAAAGGAGCTCCAACAGCACAAGATTCCAGGAAGCCA

TTCCCTCAATTCATAAAGGACAGGAGTGTGCGCTCCAATAATGGCTAATATATGTACATTATTAAACACA

AGAATGGGAGAAATCCATTCGAGAAAAAAGTATTTATTAGTTGAGATGAATCCCAATTTGGGAAGATTGA

GAGTTGAACAAGGATCCTTATTATGTATCTGCTTAAATGTTTTATTTATCCATTTTTGCTTGAGTTTCTG

CTTCGATTAGGAAGTATTGGAGCATGATTCATAATGATTGAACTGATGTGAACTTCTCCAGAATGCACTT

CTAAACACAAACATAATACTGATGGAGGAGGAATAATTGTATGGGGATAGAGGCTCCATAGAAGGCAAAC

TTTAAGGCTGCAATAATATCGTGGTCAAATATGTGCTTTCTCATTGGTGGTAAAGGTTTATTGAAGGCCC

TTTGCTTCTTCTGCTCAACAGTGGACCATGTGCTCAAAGTAAGCTCCCTACACAATGGTTGATGGAAATG

TGACTGGTCTGCATGAAGCCCTCAACCCAACTGAACAACTGTGGGATGAATTGGACCATGGATTAAGGGA

GCCAGGCCGGATCATTGAAGCCGGACCTCACTCATGCTTTTGTGGTTGAAGAGCAAATCCCACCACTAAT

CTTCCAAGACCTAGAGGAAAGCCTTTCCAGAAGAGCAGAGGCTGTTGCAGCAAAGGAATGTCTAACTCTA

TATCAAAACCTTCAGGCTTCAGAATTGCATGTTCTCATTAATAAATGTGTAGCATCGGGACTCTATAGCT

CTTAAAGGCCGTGCATGTTTGATTTAGAAAGATACACTTGATGTTATTACTGAGCTTTATACAGCACTGT

TATTCTGCACAAATAAAACTTTTACTTTACAAAAAAAAAAAAAAAAA

**48. Xl-MMP23 cDNA (CD302225 and CD302813):**

CTGACTGTTGGATAGAAGGTGCAAGAGGAGAAATCATCATTTGGACTGGTCAGAATGCATATCATTGTCAGGGAGACAAACTTAGCCCAAGGGATAACACACAGACAGGAGTGAGTCTAGGGAGCATGCAGTGCATTCCCCCAACAAGCTGAGACAAGGTGCCTGGTTAGTCTCTGCCAAGTCATCACCTATCACAGACTTCTGATAAAAAAAAC**ATG**GATGGCACCCAAGATATTGAGCATCGGAGGAAAAGGTACATCTGGGCATTTCTTGCCCTTTTTGCTGGAACAGTGCTGCTAGCTGGAATTTTCACTGTTTCTAAGTCAGTGCCCTTGGAGACAAAGGTAGACTTTGTTGTGGCTCCAACCCAGGCTCTTCAACTGCCTCTTCAGCTCCCTCGTCACCTGAGGAACAAGCGTTACACGTTGACTCCAGGTCTTCTCAAATGGGACCATTATAATCTAACGTACAGGATCGTTTCATTTCCACGGAATCTGATTAATGAAAGTGACACGAGGAAGGGGATGGCCCAGGCTTTCCAGATGTGGAGTGATGTGTCACCTTTTCACTTCAAGGAAGTCCCTGCAGACCAACATAGTGATCTAGAAATTGGTTTCTATGGCATTAATCATACAGACTGCTTGGAGTCCTATATTCATTATTGCTTTGATGGAACAACGGGAGAGTTGGCGCACGCATACTTCCCAAAAACAGGAGAAATACATTTTGATGACAGTGAATATTGGATTTTGGGTAACACATGGTTCAGCTGGAAGAAAAGAGTGTGGCTCACAGACTTGGTTCACGTGGCAGCCCATGAGATTGGACATGCACTGGGACTGATGCATTCACTCAATTCTAATGCTTTGATGCACATCAATGCCACTCTGACGGGGAAAAAACTGATCTCACAGGACGAGATATGGGGAATCTTCCGTCTATATGGCTGCAAGGATAAATATTTAGTGTGTGCCTCCTGGGCCCACAAGGGCTACTGTGACTCGAGAAGAAGACTTATGAAGAAGTATTGTCCTTATAGCTGTGACTTCTGTTATGACTTCCCATTTCCAACCAAACAGCCTACACCTCCTCCTCCTAGAACAAAAGTTCGATTGGTTCCTGAAGGCAGAAATGTTACTTTACGATGCGGGAAAAAAATCACGCACAAAAAAGGCAAAGTTTACTGGTACAAAGACAAAGAGCTGCTGGAATACTCTTACTCTGGTTACCTTAGCCTCAACGACGACCACATGAGTATAATCGCCAACGCCATTAATGAGGGACTGTACACCTGCATAGTGAAGAAGAGAGACCAGATTCTTACAACTTACTCATGGAAACATCCGACTGCAAATGTAA

**49. Xl-MMP24A cDNA (**assembled from CA791076 and EB480268 after comparisons with *X. tropicalis* MMP24 sequence.**):**

CTGCACCAATTATTGTACCTTCAGGGCTGAATGCAAAAGGAAAAATAATGCCATTGCTCCCGTGTTTTGCACTTTGCACCCTGCTCTGCAATGACGCTGGTTGAAAACATATGGCTATTTGCTTCCGTACGACATCAGAATATCCATCTTACAATCAGGAAAAGCTATGCAGTCGGCCGTCTCAGCAATGCAGCAGTTCTACGGGATTCCGGTGACCGGAGAGCTGGACCAAATGACCATTGAGTGGATGAAGAAGCCTCGCTGTGGAGTTCCTGACCATCCTCATTTAAGCCATAGAAGAAGGAATAAGAGATATGCACTCACAGGACAAAAGTGGAGGCAAAAGCACATTACATACAGTATACACAACTATACCCCTAAAGTAGGAGAGCTTGACACCAGGAAAGCGATCCGTCAGGCGTTTGACGTGTGGCAGAAGGTGACTCCTCTAACATTTGAAGAAATTCCCTACTCAGAGATCAAAAATGAAAGGAAAGAGGCGGATATCATGATATTTTTTGCTTCTGGTTTTCATGGAGACAGTTCTCCGTTTGATGGAGAAGGCGGATTCCTGGCACACGCTTACTTTCCTGGCCCGGGTATAGGAGGAGATACACATTTTGACTCTGATGAACCTTGGACTCTTGGGAACTCCAATCATGATGGAAATGATCTGTTCCTAGTTGCTGTCCATGAGCTTGGCCACGCCTTAGGATTGGAACATTCCAATGATCCCAGTGCAATCATGGCTCCTTTCTACCAGTACATGGAAACTCATAATTTCAAGCTTCCCCAGGATGATTTACAGGGAATCCAGAAAATCTATGGGCCCCCTGCAGAAACCATGGAGCCGACCAGACCTCTTCCCACTCTCCCACCACGACGGATCCATTCCACCTCTGAAAGGAAACATGAAAGGCAGCCAAGGCCCCCACGACCGCCTCTCGGAGACAAGCCACCCAGTACAGGCTCCAGGCCAAATATATGTGATGGAAATTTTAATACCGTGGCTCTCTTTAGAGGAGAAATGTTTGTTTTTAAGGATCGTTGGTTTTGGCGCCTTCGTAACAACAAGGTACAGGAAGGTTACCCAATGCAGATTGAACAGTTCTGGAAAGGACTCCCACCTAAAATAGATGCAGCTTACGAACGATCGGATGGCAAGTTTGTTTTTTTCAAGGAGACAAGTACTGGGTCTTTAAGAGGTCACAGCTGAGCCAGGTATCCCACAGCCTGTAGACTGGAAGCTGTCTCCGCNGGAGGAATAGACACGCTGCGCTGGAGACTTAGCAAACTACTCTTTAGGAACGATCTGAGATCATGA

**50. Xl-MMP24B cDNA (EB483310)**

AAAATNNNNCCATAGTNNNNNNGNGATCCATCGATTCGATTCGTCCCCGGACAAAAGTGGAGGCAAAAGCACATTACTTACAGTATTCACAACTACACCCCTAAAGTAGGAGAGCTTGACACCAGGAAAGCGATCCGTCAGGCGTTTGACGTGTGGCAGAAGGTGACTCCTCTAACATTTGAAGAAATCCCCTACTCAGAGATCAAAAATGAAAGGAAGGAGGCAGACATCATGATCTTTTTTGCTTCTGGTTTCCATGGAGACAGTTCTCCGTTTGATGGAGAAGGCGGATTCCTGGCTCACGCTTACTTTCCTGGCCCGGGTATAGGAGGAGATACGCACTTTGACTCTGATGAACCTTGGACTCTTGGGAACTCCAATCATGATGGAAATGATCTATTCCTAGTTGCGGTCCATGAGCTTGGCCACGCCTTAGGATTGGAACATTCCAATGATCCCAGTGCAATCATGGCTCCTTTCTACCAGTACATGGAAACTCGTAATTTCAAGCTTCCCCAGGATGATTTACAGGGTATCCAGAAAATCTATGGACCCCCTGCAGAAAACATGGAGCCAACCAGACCTCTCCCCACTCTCCCACCACGACGGATCCATTCCACCTCTGAAAGGAAACATGAAAGGCAGCCAAGGCCACCACGACCTCCTCTCGGAGAGAAGCCACCCAGTACAGGCTCCAGGCCAAATATATGTGATGGAAATTTTAATACCGTGGCTCTCTTTAGAGGAGAAATGTTCGTTTTTNAGGATCNGTGGTTTTGGCGCCNTCGT

**51. Xl-MMP25 cDNA (BC078136)**

GACCCACGCGTCCGCCCACGCGTCCGGGGAGAGGAAGGACAAGATGCTCAGGGGCAGTTCACACGGGGAG

ACTGAATTGTCGCACGAGTATTGTCGCTAAGGTGCAGAGTGAGGAGGAGAAGTACCGGCAGGAGACAATG

GATTTAGGAGAAGCGAGATAATAGCGCTGGAGGTGCAAGGAGAGTCCGCTACGCATTAATAAGAGAAACA

AGCTGAGCGCACAGAGCGCGGACACACCGGGGCGGAGCCGCAATATTCGGGAGAGAAGCGCAAGTTTAGG

CGCAGCTGAGCAGGAGAATCTGGAAGAGGCGCAAGTTTAGGCGCAGGAGAATCAGGCGCAAGTGGCGGGT

GAGAG**ATG**TTGGCCGTGTGGCTGCTGTGGGGGAGTCTGGTGATAACTGGAGGGAACCCGAGCGCAAGAGA

CATCAGCAAAGGACTGGACTGGCTGACGAGATATGGCTACCTTCCACCTCCAGACCCTTTTTCAGCTCGG

CAGCAAACACTGGAGGGGCTTCGCGAGGCAGTCAAGACTATGCAAAGGGTAGCAGGCTTGCCAGAAACCG

GGGAACTGGATGATGCAACTGTGCACATGATGAATAAGCCACGATGTTCACTTCCCGATATCATTATGAG

ACCCGAGCGCCGCTCTCGTCGCAATAAGAGATATGCCCTGAGTGGTTCTGTGTGGGATAAAAAGCTGCTG

AGTTGGAGGTTGGAAAACTCCCCCAGTACCCTGCCCCATGATGTCACCCGCACCCTGGTAAGCACTGCTC

TTGCAGTCTGGAGCCAAGAGACGCAACTGCGATTTAGAGAAACGCAAGACAAGCCTGACATACATGTAGG

ATTTGTTGCTGGGTCCCATGGAGATGGCTATCCCTTCGATGGTCAAGGAGGAACTCTGGGTCATGCTTTC

TTCCCAGGTGTGGGGGAAAGAGCTGGAGAAACTCACATGGATGCTGATGAATCTTGGTCATATAATACCG

AGGACGGTACCGACCTCTTTGCAGTGGCAGTTCATGAATTCGGACACTCCCTAGGGCTGTATCACTCATC

CAGTGAAAACTCCATCATGAAGCCATATTATCAGGGGACTGTAGGGGACCCTAGCAAATACCGACTGCCC

CCTGATGATGTGGAGGGGATCCAAATTCTTTATGGTCGGCCAGACTCTGGGTATAGACCTCCTGCTGTTA

CACCAACCAGACGCGCCCTTCCCCCACGTGGGCCCACTCCGGGACCCCGTCTCCCATTCCCAGATCGCTG

CTCAACCAACTTTGATGCAATTGCCAATATCCGAGGAGAAGTGTTCTTCTTTAAGAACAGGTATTTCTGG

AGAGTGCAGTCCTCAAGGCAGTTGGTCTCACTCAACCCTGCTCACCTCAACCGTTTTTGGCTGGGGCTCC

CTCCAGACTTGCCTAAATTGGACGCTGTGTATGAGAGAACCAATGACAGCAAAATTGTGTTTATTGCAGG

AAACTCATACTGGGTTTTCAAGGACACCTTGGTGGAACCAGGATACCCTCGGCCTTTGTCCGACTTTGGA

CTCAATACAGACGGCGTTGATGCAGCTTTTGTTTGGAAGCACAACGGGAAGACCTACTTCTTCCGCAAAA

AACTGTTCTGGAGGTTTGATGAAAAGAGAGGACAGATAGACTCGGGGTACCCGAAAGACAGCAGCTTATG

GGAAGGCGTGCCACCTGATATTGATGACATAATAAGCTGGGAGAATGGTGACACGTATTTCTTCAAGGGC

ATGCAGTATTGGAAATTCCAAGGTGGCAACGTGGCGGCAGAGCCGGGATATCCGCAGAGCACAGCACTGA

ACTGGATGTACTGTCCTTTTGAAGGCCCCGCCATTCCTACAAATGCCCCAGAAGGCAGGGGTCAGAGAGA

CTGTAGCTGCACTTGCACCGATGGAAGGAATTCAGCGACACTGATTGGCATCCTTGGGGCGGCGTGGTTG

GCTTTTGCCTTTCATTTGCTGCTGCTTCTGTGCTGACAATTGCAAAGAAAATTGCACGGACACACTCTAC

AGTGTAGCCCTGAGTGGAGTTTACTTTAAAAAGCAGCCACACAACACGCACAGCATGGACAACATTATTA

AATGATATCGACCAACTGCAGACAATGAACTGAGGGGACTCTCTCTATATGGAGCCCAGCACATTCTGCA

GTCCCAGGCAGTGACTAATACAATCTCCTGGTTTTGCCTATTCTTTTATTGACAGAAAGGATTCTCTCGC

TGTACAGATTACAAAAAAAAAAAAAAA

**52. Xl-MMP26 cDNA (BC056080)**

CTGACTTATTAACTTGCCACC**ATG**CTACAAGTAGTCTTCTTAGCTATCTTGTCCCTGTCCTGTATCCTTG

CTATGCCAGCGTCTCCCACCAACAATAACATCAGCCCGTCGGACCGCACATTTGCAGAGACTTACTTAGA

CAATTACTACTTGATGACAGCAAAATCAAAGACCACCTTTGTGGAAAAAATTAAAGAGATGCAAAAATTC

TTTGGAATGTCGGTGACAGGGAGGTTGGACTCAGATACCATGACAATGATGAAGACCCCTCGATGTGGAA

TGCCCGATGTTGCGGAATTTAGACAGTTCCCTGGGAGACCAAGATGGGCAAAAACCCAACTATCATACAG

GATTGTGAATTACACTCCTGATCTTCCCCGCCCAGTGGTAGATGAAGCAATAAGGATGGCATTTAAAGTG

TGGAGTGATGTAACGCCACTAAAATTTACAAGAGTTTCCTCCAGACGAGCAGACATATTAATCCAGTTTG

GAGCACGCTCTCATGGAGATGGTATCCCATTTGATGGCCCTAATGGAGTTTTAGCTCATGCCTATGCTCC

AGGTAATGGCATAGGAGGTGATGCCCATTTTGACGAAGATGAAAGATGGACAAGTTCTAGTGCAGGTTTC

AATCTGTTCCTTGTTGCTGCCCATGAATTTGGCCACTCACTGGGATTAGACCACTCCAGAGATGCACGAG

CTTTGATGTTTCCAAACTATCGTTATGTGAACACCAGAAACTTCCGCTTGCCGCAAGATGACGTCAATGG

AATACAGTCAATATATGGGAGAAAATAGTAGCAGTTCTACACTAGTTGGGTCTTTTATGTGAATGTGTCA

GATAAACATAGACACATGTTCAGGGCTTTAGCATGATGGTCATTGTATTGCTATATCACAGGAAAATATA

ATCAGCCAACCCTAAGCATTTGTTACATATGGTAATGTTTCCCTTAATATACCCGCAAGCAGAACCTTAG

CACTGAGGTGCAGTATTATTATGAAAGCATAAGTGAAATGAGCTGTAGATAACTAAAGTATTTTCCTAGA

ACCCTGAGGGCTGGAGAGGATATATGAGTAGAAAGATACTTAGGAAATACTAAAGAAATGCAAAAATGAG

TTGCCATCTTGGCAATATTGCTAGAGTAACAGCTTATTTCTCTTTGAGGATGCACATTGTTTCCCAACCT

GGCTCTCTAGCATTGGTTCATCCATGATTTGTCTGAAACCAGATCAATGTCTTCTTCTCACACAGCTGCC

AAAACCCCAGAACAATATTATTTTGCAGGACAAAAAAAAACACCTTTATTTTGTGTGCGCCTATAGCATT

GTCTGGAGAGTTCTACAGGACCTGTGAGCAACATCTCATTGTGAATGTAGCTATGAATACCTGGAAGGGA

TAGACAGTTTGTCTTTAGAAATGGTGGCTGAGAATAAGTCCCATCCATCAGCCAAAATTGCTGCATAATT

ACAGTAGCATGCAATATGAAAAAGCTCCAGTAGGTTGTGTACCAATCACGTTTATACTATGCAGGAATGA

ATGATGCACAATACATAATAAAGACATTTGTATCGCTGTGTAAAAAAAAAAAAAAAAAAAAAAA

**53. Xl-MMP28A cDNA (EF187277)**

GCACACTTGCAAGTGGTGAGCAGCTACAGGTCTCAATCCAAGGACTATACCATGCCTGGCTGAGAAGAACAGCAGAAGTCTCTGATAACTCTGCCAAACTCTACACTTGGGAAGTAATATGACAACTGCTTGTCCAGAAACAGTGCAGAACAGCACTCTAAAGAATTCAATTCACTTGTAGAAGATGGACTGGAGATAAGTACGATACAAGTACGGCATGGAGCAAGATTTTTATAAACCATCAAATTCAACAAGAGCTTTATTCTGACCATATGAAAATGACTTTGCTTCAGACTCTGTAGCTGATCAGCATGTAAGCATCACTGCATTGTTTAATGGATTAGTAACTTCTCTAGTGCCTGCAAAATTCCCTTGGAA**ATG**GAAGCTGATATTCCATCCCTGTTCCTTCTGCTTGTGATAACTGGTCTGTGCTTGTGCAATGGCTACATATCTGAAGAAACCTTACAAACTGCACAGGTGTTTTTGGAAAAATATGGCTACCTTGAAGAAACAACAAAACAACATAGTGGAAAACAGCTTGCATCAGCAGTCAGAGAGTTTCAATGGCTGTCCCACCTGTCAGTCAGTGGAGAGCTGGACACCTCTACAGTACAACAGATGATCCAGCCTCGATGTGGCGTGAAGGATATTGAATCACTAAAACTGGTAAAAAGTCACCATCACGGTCGACAACGCAAGAAACGTTATATATCCAAAAGTAAGAAATGGTACAAACAACATTTAACTTATCAGATTGTCAACTGGCCCTGGTACCTATCCCAGCATCAGGTCAGGCAGGCGGTTAAAGCAGCGTTCCAGTTGTGGAGCAACGTTTCCTCTCTGACATTCTCGGAGGCTCTAAGAGACCCTGCAGACATCCGTTTAGCATTCTTTGATGGGGATCACAATGATGGGGCTGGAAATGCTTTTGATGGACCAGGTGGTGCCCTGGCACATGCTTTTTTCCCCAGAAGGGGAGAGGCTCATTTTGACAGTGCCGAGCACTGGTCATTGAATGGTAAGGGCAGAAACCTGTTTGTTGTACTGGCACATGAGATCGGACATACTTTGGGGCTTCCGCACTCCTCCTTCAAAAATGCTCTGATGTCCCCTTACTACAAGAAACTGAATAAAGACTATGTACTGAACTTCGATGATGTCCTGGCCATCCAAAATTTGTACGGAGCTCCCCCTAGTGGAAATCTGGTGCAACTCCCTGGAAAACAATTTGCCTTTTTTCAGGATTGGAGTCCAGAATCACATGAAGATTCTGGAATGAAACCTTCCTACTGCCACTCTATCTTCGATGCCATCACGTGGGATTTGAAAAAAACATTATACATCTTTAAAGGCCGTCACTTTTGGATGGTCTCATTGGGTGGTAAAATCTCACCACCCCAATCTCTTCAGAAGCGCTGGAAGAAACTCCCTTCTTACATTGAAGCTGCAGTGGTATCAGGTTTAGATGGAAAATTCTACTTCTTTAAAGGTGGAAGGTGTTGGAGGTACAAAGATTCCATATTAGAAGAAGGCTTTCCTCAAAAATGTAGCATGAATGGTTTGCCTCGTCGTCCTGACACTGCACTGTACTTTCAGCCTCTTGGTCACTTGGTCATCTTCAAGGGCTCTAAATATTATGTGGTGAATGAAGAGTCTCTAACAGTGGAACCTTACTACCCACGCAGCTTGCATGACTGGAAAGGTGTGCCTGCCAACAGTCACAGTGTTCTGACCCATCCTGATGGAGCCATCTACTTTTTCAAAGGTCACCAATACTGGATATTTGACCAAAAGAAGCTGAAAGTAACTACTTCTGGGAAGTGGGCTGAAGATTTGTCTTGGATTGGTTGTAAAAATGATGTGACTTGATTTTTCTTCAGTGTTAAAATCACTTCACTTGTACTACATAATCTTGTATATAAGACCTACCAAGGAATAAAATGTAGGACAACTTCAAAGGAAAAAAAAAAAAAAAAAA

**54. Xl-MMP28B cDNA (BC061659)**

AAAGATTTTATTTTTACTTGTAGAAGATGGACTGGAGATTAGTATGATTGACTAGTAATGCAGAACTGCTGTCTGTATGGACTAATGACACCAAGTTGCTACTGCCTGGAAGTATTAAAAAGGCACAATGATTTTCATAACCCATCAAAATCAGAGAGCTTTGTTCTGACTGTATGGAAATGACTTTACTTCATACTCTGTAGCTGACCAGCATGTATGCTTTACTGCAGATGGCTGTGCATTACTGTATTGTTTAATGGATGAGTAACTTATCTGGTGCTTGCAAAATTAACTTGAAA**ATG**GAAGCTGCTATTCCATCCCTGTTCTTTCTGCTTGTGATTGCTGGTTTGTGCTTGTGCAATGACTACATATCTGAAGAGACCCTACAAACTGCACAGGTGTTTTTGGAAAAATATGGCTACCTTGAAGAAACGACTAAACAACATAATGGAAAACAACTTGCATCAGCAGTCAGAGAGTTTCAATGGCTGTCTCACCTGCCAGTCAGTGGACAGCTGGACACCATAACAGTACAACAGATGGTACAACCTCGATGTGGCATGAAGGATATTGAATCACTAGAACTGGTCAAAAGTCACCATCATGGTCAGCAACGCAGGAAACGTTATACATCCAAAAATAAGAAATGGTATAAACGACATTTAACTTATCAGATTGTCAACTGGCCCTGGTACCTATCCAAGCATCAGGTCAGGCAGGCGGTGAAAGCAGCGTTCCAGTTGTGGAGCAACGTTTCCTCTCTGACATTCTCTGAGGCTCTGCGAGACCCTGCAGACATCCGTTTAGCGTTCTATGAAGGGGATCACAATGATGGTGCAGGGAATGCTTTTGATGGACCAGGTGGAGCCTTGGCACATGCTTTTTTCCCCAGAAGGGGGGAGGCCCATTTTGACAGTGCCGAACACTGGTCATTAGATGGTAAGGGCAGAAACCTATTTGTTGTACTGGCACATGAGATTGGACATACTTTGGGGCTGCAGCACTCCTCCTTCAAAAATGCCCTGATGTCCCCTTACTACAAGAAACTGAATAAAGACTATGTACTGAACTTCGATGACGTCCTGGCCATCCAAAATTTGTACGGAGCACCCCCTAGTGGAAATCTGGTGCAACTCCCTGGAAAACAATTTGTGTTTTTTCAGGACTGGAGTCCTGAATCACATGAAGATTCAGGAATAAAACAGTTTTCCTACTGCCACTCCATCTTTGATGCCATCACATGGGATTTGAAACAGACGTTATACATCTTTAAAGGCCGTCACTTTTGGACAGTCTCATTGGATTGTAAAATCTCACCTCCCCAAACTCTTCAGAAGCGCTGGAAGAAACTCCCTTCTTATGTTGAAGCTGCAGTGGTATCGGGTTTAGATGGAAAATTCTACTTCTTCAAAGGTGGAAGGTGTTGGAGGTACAACGATTCCATGTTAGAAGAAGGCTTTCCTCAAAAATGTAGTATGAATGGTTTGCCTCATCGTCCTGACACTGCACTTTACTTTCAGCCTCTTGGTCACTTGGTCATCTTCAAGGGCTCTAAATATTATGTGGTTAATGAAGAGTCTCTAACAGTGGAACCTTACTACCCACGCAGCTTGCAGGACTGGAAAGGTGTGCCAGCCAACAGTCACAGTGTTCTGACCCATCACAATGGAGCGGTCTATTTTTTCAAAGGTGACAAATACTGGTTATTTGACCAAAACAAGCTGAAAGTAACTACTTCTGGGAAGTGGGCTGAAGACTTGTCTTGGATTGGTTGCAAAAAGGATGCAACTTGATTTGTCCTTCAATGTTAAAATGGCTTAATTTTTTTCTGCATAGCCCTAAGAATCTCAATCCTGTATTGCCATAAAGATATACCAAAGAATAAAACCTTGGACAGCTTCAAAAAAAAAAAAAAA

**55. Xl-MMP N1 cDNA** (assembled from BJ032306, BE509380, EC276067, BX852582, BJ047339 and BG578455)**:**

TTGGACAGTTTGGCTGATTATTTTGCCACTAGGATCCACTAGACCAATTGAAGAAGAATCTGATAGTGTGGAATTTAGAGCTACCCCTTCCCCAGAGGTCAACGTCCCCCCAAATTTGGAACCCCGTGGCACAAAACCAGAAGGACCAGTCCAGATAGACACAGAACAATTTAAGTTGGCAGCGGACTATCTGATAGAGTTCGGGTATCTCCCAGATGAGAACCTTACAGAATACTCTGTGCCCAACACTCCGTTTATCTCTGAAGATGAGCTGCCAGAGGAGTTTATCAGCGGCTTGGAGTGGTTCCAGAGACAGAATGGGCTGAAAGTGACTGGTAAATTAGACCCTGACACGGCTGAGGCGATGAAGCTTCCCCGCTGTGGGAAACACGAGCAACGCATGTCATACAATGTGGGCTCTAAGTGGAAGAAAGATACGCTGACCTACAAAATCCTTAATACAACAGCCCAGCTGCCTGAAAAGTTAGTCAAGGATGAGTTAAGCAAAGCCTTGAAGGTCTGGCAAGATGTATCTTCTCTGAAATTTGTAGAAGTTGGCACCAATGAAACAGCAGACATTGATATGTTCTTCGTCTCCGGCCTGCATAACGATGGCGCTAAAAATGCCTTTGATGGTCCTGGTCGTGTTTTGGGCCACGCGTTCATGCCTCCGTTCGGTAAAACTAAAAGGGACATTGATGGAGACCTTCATCTGGATAATGATGAGAAATGGACAATTAATGAGAAGAAAGGGGTAAACCTTTTGCAGGCAGCTGCACATGAACTTGGACATGCTTTGGGGCTAGATCACTCAACTATACCAGGAGCTCTCATGGCCCCAACTTACAAAGGCTACAACCCCAAATTCCAGCTTCACCAGGATGATATTCAAGCCATCCAGGTTCTTTATGGCAAACCAAAGCTTAATCAGACCACAGCAACTAACACCACGGACATCAAGGCTGGAGATGTGAAAAAAGACCCAAAACCCACCACGAAACCCAAAGCAGAAGATAAGGATGCCAAAAAGCAATCAGTGGTGAAGATGTGTGGGGAACAACCAATTGATACATTTTTATCCATCAAGAATGGCTCCATCTATTTATTTAAAGGTGAATACTTCTGGGAGATGAGTCACGGGAAGCTGCCATTAACAACCAAGAAGAAATACCCTCAGCTTATTTCAACAAAGTGGAAATCCTTGCCCTCTTCGATTGACGCAGCCATAAGAATGCAGAACCCAACTGCAGATCAGGATGGAAAGATCTTCTTCTTTAAGGGACGTAATTACTGGAAGTTTGACAATGGTCAAATGGAACCTGGATACCCAAAGTTGATCAGTGAAGGGTTCCCAGGTGTTCCAGACCATCTGGACGCTGCATTCACGCAACCTGCTATCATAGCAAAAGGAGGGAAAGTTATAAGAGAAGAGAGAATTTTCTTCATAAAAGGGAAAAACTTTATCGTGTACAACCCTGGTACTGGGAATAGCACCAGCGCTCAGTCGTTGCAAGATGATTGGGTGGGAGTCAAGCTGCCAATCAGTGCAGCCCTCAGTCTGAAGAATGAGATGTTCCTCATCAGCAAGAAGAAGTTCCAGAAGGTCCTCATGCTGACTTATACCCAGGATCACGTGTACGGCAACATACACCAGCCCAAGAATCTAAATCAGTTGCTTCAATGCGAGTGAAGCTATGGAACTGTACAGATATATGTAAATAATTGTAGCTTCCTAGTAGTCGTCCACTGACTGTAAGCTCTTCTCTAGTCTACTAATGACATTCAGCAGATTGTTTTAACTCCTTGATTATCACATATACGTAAAATCTTAGAACACACTAAATTCCAAAG

**56. Xl-MMP N3 cDNA** (assembled from BG234242, BU905338, CB558404 and CF547511):

TGATTTCAGAAGGAGAACCCGAGGAGCATCCTAGAGTCAGTAATTCAAATCAAACTCAGATGAATCTATCCTTCGTAGACTCCTTGAAAAAATTCCAGGAGGCAAACGGGCTAAATATTACAGGAGTCTTGGACAGTGCTACAAAGATAGCCATGAACAAGCCACGCTGTGGTGTTCCAGATTTCAAAGTTGCATCAAGACGCAGGAATGCAACTCTCGGCTCAATGGATGCAGATAGTAACTCTCACCACAATAGCTCACAAATGCAACGTAGAAAGAGAAGTTTCCTTTCTAAAATGGTTGAACAATACAGGCAAAATCGAGAATCCAAAGACAACATGGGAAGTGGCAATTCCAAGAGGTTTTCCAGAAATACGCTGAAGTGGAGGCTGATGGGAGAAGGGTACAGCATGCAGTTGACCATACAACAACAGAGGGCAATTCTTGCCTTGGCTTTCCGTATGTGGAGTGAGGTTGTCCCTCTGCAGTTTGTGGAGGACCTCACAGGAGATGATATAGATATTCGAATTGGCTTTGGAACAGGACAGCACCTTGGCTGCTCACAGGCATTCGATGGGGTGGGGCAGCAGTTTGCTCACGCCTGGTACCTCGGAGATATTCATTTTGATGATGACGAACATTTTGTTGGACCATCTAGTGAGCATGGAATCAACCTTTTGAAGGTTGCAGTTCATGAGATTGGTCACGCTCTCGGACTGAGCCATATAAACCGTGAAGGATCAGTCATGCAGCCTAATTATATACCTCAGGAACGCCATTTTGAGCTTGACTGGGAAGACAGAAAAGCAGTGCAGGAAAAATATGGTGCCTGTGATGGCAGCTTCAGCACAGTTTTTGATTGGATAAGGAGAGAACGAACTCCACGTGGAGACACAGGGTTTNGATTTAACACGTATTTCTTCAAGACAAGCTGGTACTGGATGTATGAGAACAAGGGCAATCGGACACGATTTGGGGACCCACTGCCTATCAAAGGAGGCTGGAATGGTATTCCGGATAAAGACATTGATGCCTATGTTCATGTGTGGACTTGGAACATCGATGCACAGTACTTTTTCAAAGGTACCCTAGTCTGGCGCTATGACCCTGACAAAGACAGGGCATTTACAGAGGACTGGAGGAAAGCCAAGTATCCTCAGCCTATTGTAGAAATATTTCCAGGAGTCCCAAGTCCAGTGGATGCGGCTTTTTTCAACAAAAAGGAGCGCTTTATCTACTTCTTCAGAGGAAATAACGTTACTGCCTTTAGTGTTGATAAAAATCAAAAAGTGGAAAAGTTCCCAAAACCCATCATTGACATATTCCCTCCTGTGGATCCCAATGACCACCCCATAGGGAATATCGATGCCGTTTATTTCTCATACTCATACCAAACAACGTTCTTCATCAAAGACAAATATTTCTGGAAGGTGGTTAGTGATCGAGAAAGACAAAGCAACTCATCTCTGCCTGTGAATGGATTATGGCCCCGGAAGAAAATCAACTCCCAGTGGTTTGATATTTGTGATGTCCATCCTTCTGTACTCTTGCTGTCAACATAAGGAGGAACTGGTAATGCTTCCACGAGAAGGACTGTATGGAGTCAATGTCTATGAATGAATTTCACCTTCAACCATAACCTGTGGACAACCGACAGACTTCTGCTGTGCAAAGAATACTAGAATGTTGGGCAATCATTGGTTCTTTAATAAAGTACCATCTTATTATTATTAGATTAGTGTAGCT

**Additional file 1:** The nucleotide sequences of *X. tropicalis* and *X. laevis* MMPs used in this study. The coding region for each MMP is underlined, and if applicable, the start codon was in bold and the stop codon was shadowed.
