# Supplementary figures and images for: Genome-wide identification of Xenopus matrix metalloproteinases: conservation and unique duplications in amphibians
Source: BMC Genomics. 2009 Feb 17;10:81. doi: 10.1186/1471-2164-10-81 (PMC2656525; doi:10.1186/1471-2164-10-81)

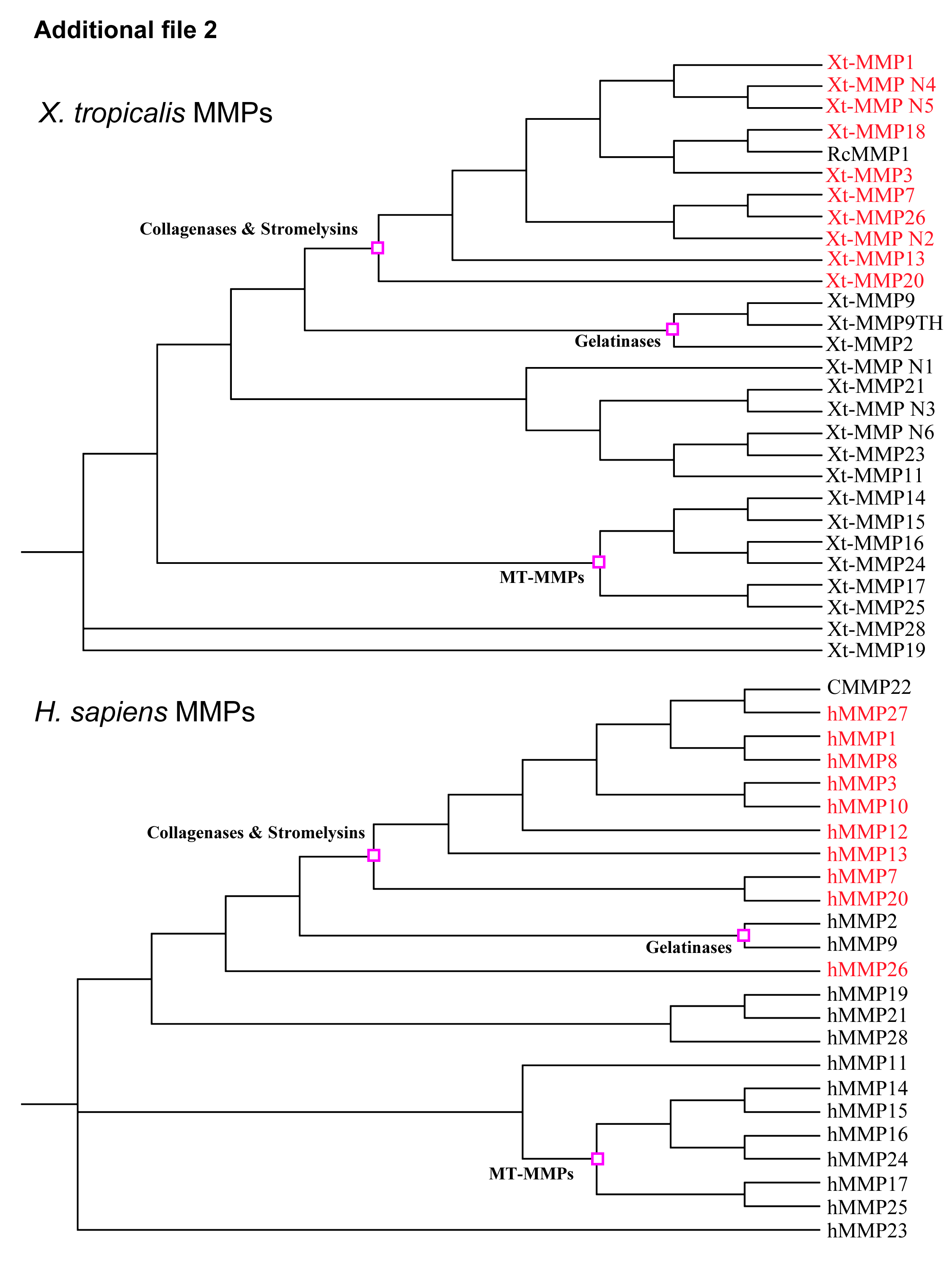

Supplement: Additional file 2 — Phylogenetic trees of X. tropicalis and human MMPs. X. tropicalis MMPs along with Rana catesbeiana MMP1 (RcMMP1) or human MMPs along with chicken MMP22 (CMMP22) were analyzed using the multiple sequence alignment program CLUSTALW to generate the corresponding phylogenetic trees with defined ancestral nodes marked by purple square. The MMPs located on human Chromosome 11 and those located on X. tropicalis Scaffold_119 are in red. [file 1471-2164-10-81-S2.tiff]
